# Optimal neuromuscular performance requires motor neuron phosphagen kinases

**DOI:** 10.1113/JP288916

**Published:** 2025-11-22

**Authors:** Karlis A. Justs, Danielle V. Latner (nee Riboul), Carlos D. Oliva, Yosuf Arab, Gabriel G. Bonassi, Olena Mahneva, Sarah Crill, Sergio Sempertegui, Paul A. Kirchman, Yaouen Fily, Gregory T. Macleod

**Affiliations:** 1 Biology and Neuroscience Graduate Program, Department of Biological Sciences, Florida Atlantic University, Boca Raton, Florida, USA; 2 Wilkes Honors College, Florida Atlantic University, Jupiter, Florida, USA; 3 Institute for Human Health and Disease Intervention, Florida Atlantic University, Jupiter, Florida, USA; 4 Department of Physics, Florida Atlantic University, Boca Raton, Florida, USA; 5 College of Arts and Sciences, University of South Florida, Sarasota, Florida, USA; 6 Stiles-Nicholson Brain Institute, Florida Atlantic University, Jupiter, Florida, USA; 7 Tulane Brain Institute, New Orleans, Louisiana, USA; 8 Department of Physiology, Tulane University School of Medicine, New Orleans, Louisiana, USA

**Keywords:** mitochondria, phosphagen kinase, physiology, presynaptic

## Abstract

Phosphagen systems are crucial for muscle bioenergetics by rapidly regenerating ATP to support the high metabolic demands of intense musculoskeletal activity. However their role in motor neurons (MNs) that drive muscle contraction has received little attention. Here we knocked down expression of the primary phosphagen kinase (arginine kinase 1 (ArgK1)) in *Drosophila* larval MNs and assessed the impact on presynaptic energy metabolism and neurotransmission in situ. Fluorescent metabolic probes showed a deficit in presynaptic energy metabolism and some glycolytic compensation. Glycolytic compensation was revealed through a faster elevation in lactate at high firing frequencies and the accumulation of pyruvate subsequent to firing. Our performance assays included two tests of endurance: enforced cycles of presynaptic calcium pumping and, separately, enforced body-wall contractions for extended periods. Neither test of endurance revealed deficits when ArgK1 was knocked down. The only performance deficits were detected at firing frequencies that approached, or exceeded, twice the firing frequencies recorded during fictive locomotion, where both electrophysiology and SynaptopHluorin imaging showed an inability to sustain neurotransmitter release. Our computational modelling of presynaptic bioenergetics indicates that the phosphagen system’s contribution to MN performance is likely through the removal of ADP in microdomains close to sites of ATP hydrolysis rather than the provision of a deeper reservoir of ATP. Taken together these data demonstrate that, as in muscle fibres, MNs rely on phosphagen systems during activity that imposes intense energetic demands.

## Graphical Abstract

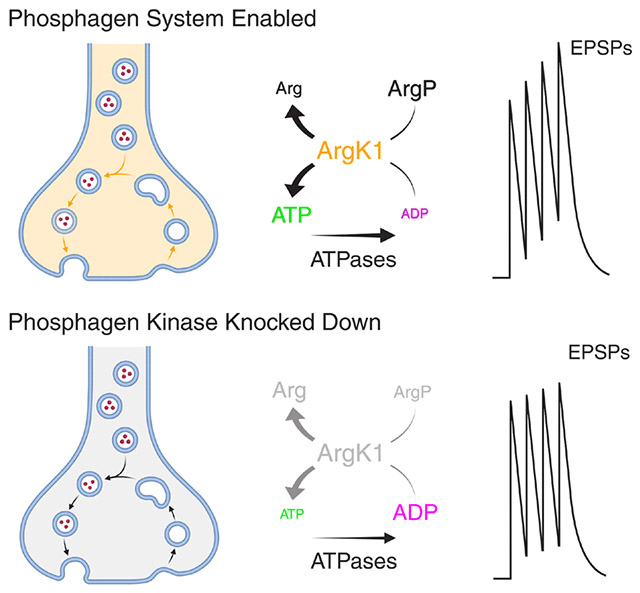

Motor neuron (MN) terminals in fruit flies, mice and humans contain phosphagen kinases, a key enzyme in their energy storage and buffering system. Here we knocked down the primary phosphagen kinase in fruit fly larvae (arginine kinase 1 (ArgK1)) but were surprised to find that MN endurance was unaffected. In contrast these MNs were unable to sustain release during high-frequency bursts that register as excitatory postsynaptic potentials (EPSPs) in the muscle fibre. We concluded that the deficit resulted from slower clearance of ADP, and replenishment of ATP, in microdomains surrounding the ATPases that power several steps in the synaptic vesicle cycle.

## Introduction

Phosphagen kinases play an integral role in the bioenergetics of skeletal and cardiac muscle where they catalyse the conversion of phosphagens to ATP and vice versa (Wallimann et al., 1992). Phosphagen systems maintain deep reservoirs of phosphagens, such as phosphoarginine in fruit flies and phosphocreatine in mammals. These reservoirs make ATP available at times of intense metabolic load acting as ‘temporal’ buffers between energy supply and demand (Newsholme et al., 1978). ATP and ultimately phosphagens are subsequently regenerated by glycolysis and oxidative phosphorylation at a more leisurely pace (Hird, 1986). Phosphagen systems also play ‘spatial’ buffering roles which ameliorate the limitations caused by the relatively slow rates of ATP and ADP diffusion in the cytosol (Meyer et al., 1984). Benefits accrue through a close physical association between phosphagen kinases and the sites of both ATP production and ATP consumption. The colocalization of phosphagen kinases with the machinery of glycolysis (Kraft et al., 2000; Maughan et al., 2005) and oxidative phosphorylation (mitochondria; Rojo et al., 1991; Wyss et al., 1992) improves the efficiency of ATP synthesis as the kinases remove ATP as it is synthesized and regenerate ADP – the substrate for further ATP synthesis (Schlattner et al., 2016; Wallimann et al., 2011). Similarly the colocalization of phosphagen kinases with pumps and other ATPases (Schlattner et al., 2016) provides benefits as the rapid removal of ADP and regeneration of ATP maintain the free energy available from ATP hydrolysis (Ellington, 1989; Iyengar, 1984; Wallimann et al., 1992). This phenomenon is especially evident in studies demonstrating that phosphagens can provide a more effective energy substrate than ATP itself (Korge et al., 1993; Minajeva et al., 1996; Xu et al., 1996). Strategic colocalization with phosphagen kinases dictates which ATPases get privileged access to ATP from the phosphagen reservoir (Linton et al., 2010; Meyer et al., 1984).

Phosphagen systems play an important role in nervous tissue, including central neurons (Andres et al., 2008; Lipton & Whittingham, 1982), and removal of different creatine kinase (CK) isoforms leads to various behavioural and spatial learning deficits in mice (Jost et al., 2002; Streijger et al., 2004; Streijger et al., 2005). However their role in neuronal bioenergetics and synaptic performance *per se* remains unclear. Previously we established that mitochondrial-associated phosphagen kinases can be found in *Drosophila* motor neurons (MNs) and the lower MNs of mice (Justs et al., 2023), as reported for humans (Lowe et al., 2013). Furthermore they are found in the MN axon terminals (Justs et al., 2023). In mammals phosphagen kinases are represented by five CK genes, the products of which form isoenzymes that either reside in the cytosol or target mitochondria (Bertin et al., 2007; Wallimann et al., 2011). Knock-out (KO) of mitochondrially targeted isoenzymes exhibits little impact on muscle performance phenotype (Steeghs et al., 1998; Steeghs, Heerschap, et al., 1997), whereas KO of cytosolic isoenzymes results in a nuanced phenotype where tetanus force is diminished but initial twitch force and endurance are relatively unaffected (Steeghs, Benders, et al., 1997; van Deursen et al., 1993). In *Drosophila* phosphagen kinases are represented by three arginine kinase (ArgK) genes (*CG4929*, *CG5144* and *CG4546*), with only one (CG4929, ArgK1) showing significant expression in the larval nervous system (flybase.org). Although we previously demonstrated that knock-down (KD) of *Drosophila* ArgK1 compromises presynaptic energy metabolism (Justs et al., 2023), we did not investigate the extent to which neuromuscular performance relies upon an intact phosphagen system.

Here we developed a number of imaging techniques to determine the impact of ArgK1 KD on presynaptic energy metabolism, the ability of MN terminals to pump calcium ions (Ca^2+^) during burst firing and the ability of larvae to sustain body-wall contractions. Using a new suite of fluorescent probes (ATeam^YEMK^, LiLac and Pyronic) we demonstrated the impact of ArgK1 KD on ATP, lactate and pyruvate levels and found the data to be consistent with previous observations (Justs et al., 2023). Our electrophysiological assays revealed only deficits in neurotransmission during high-frequency nerve stimulation. To address the difficulty in studying neurotransmission at neuromuscular junctions (NMJs) under physiological conditions – such as repeated high-frequency bursts, where muscle contractions displace electrodes – we developed two MN performance assays to simulate *in vivo* firing. Our findings suggest that the ArgK1-enabled phosphagen system in MNs has minimal effect on endurance but may influence the power exerted at the start of body-wall contractions.

## Methods

### Fly stocks

*Drosophila* stocks were raised on standard cornmeal food (Bloomington Drosophila Stock Centre (BDSC) recipe) at 22 ± 1°C. Experiments were performed on female third-instar larvae with transgenes expressed on a *w^1118^* isogenized strain background. BDSC (Bloomington, IN) provided the following fly lines: OK371-GAL4 (26160), OK6-GAL4 (64199), nSyb-GAL4 (51635), UAS-mito-Green Fluorescent Protein (GFP) (8442), UAS-myr-tdTomato (32222), ArgK1-GFP (51522), UAS-ArgK1 dsRNA (41697), UAS-ArgK1 dsRNA (35221), P{CaryP}attP40 (36304), P{CaryP}attP2 (36 303), UAS-luciferase (35788), UAS-Dak1 dsRNA (65107), UAS-Adk2 dsRNA (55320), UAS-Adk3 dsRNA (57407) and UAS-ChR2(H134R) (28995). UAS-Tag Blue Fluorescent Protein (TagBFP) and UAS-SynaptopHluorin were provided by Dr Kenneth Irvine and Dr Gero Meisenbock, respectively. UAS-mito-mKate was prepared in this laboratory and described previously (Justs et al., 2023). Lines enabling the expression of ATeam1.03^YEMK^, LiLac, Pyronic and oxidation-resistant BFP (oxBFP)-tagged ArgK1 exons were made as described later, along with Crisp/Cas9-modified ArgK1 alleles allowing pHusionRed tagging at either the 5′ or 3′ end of the ArgK1 catalytic subunit.

### Genetics

#### Transgenics.

GenScript (Piscataway, NJ, USA) biosynthesized ATeam1.03^YEMK^ DNA (Imamura et al., 2009), which was then subcloned into the Multiple Cloning Site (MCS) of pJFRC14 producing the UAS-ATeam1.03^YEMK^ plasmid. GenScript biosynthesized the DNA of LiLac (Koveal et al., 2020) 5′ to the DNA of a viral P2A peptide which was 5′ to the DNA of Tag-RFP-T, which was then subcloned into the MCS of pJFRC14 producing the UAS-LiLac-P2A-Tag-RFP-T plasmid. Pyronic DNA (San Martin et al., 2014) was obtained from Addgene (51308) and subcloned into the MCS of pJFPC14 producing the UAS-Pyronic plasmid.

UAS-ATeam1.03^YEMK^ and UAS-LiLac plasmids were injected into *w^1118^* embryos containing the attP40 landing site by Rainbow Transgenic Flies (Newbury Park, CA, USA). UAS-Pyronic was injected into *w^1118^* embryos containing the attP2 landing site. Strains with transformed germ line cells were balanced and other chromosomes outcrossed to an in-house *w^1118^* line.

**Figure A1.**
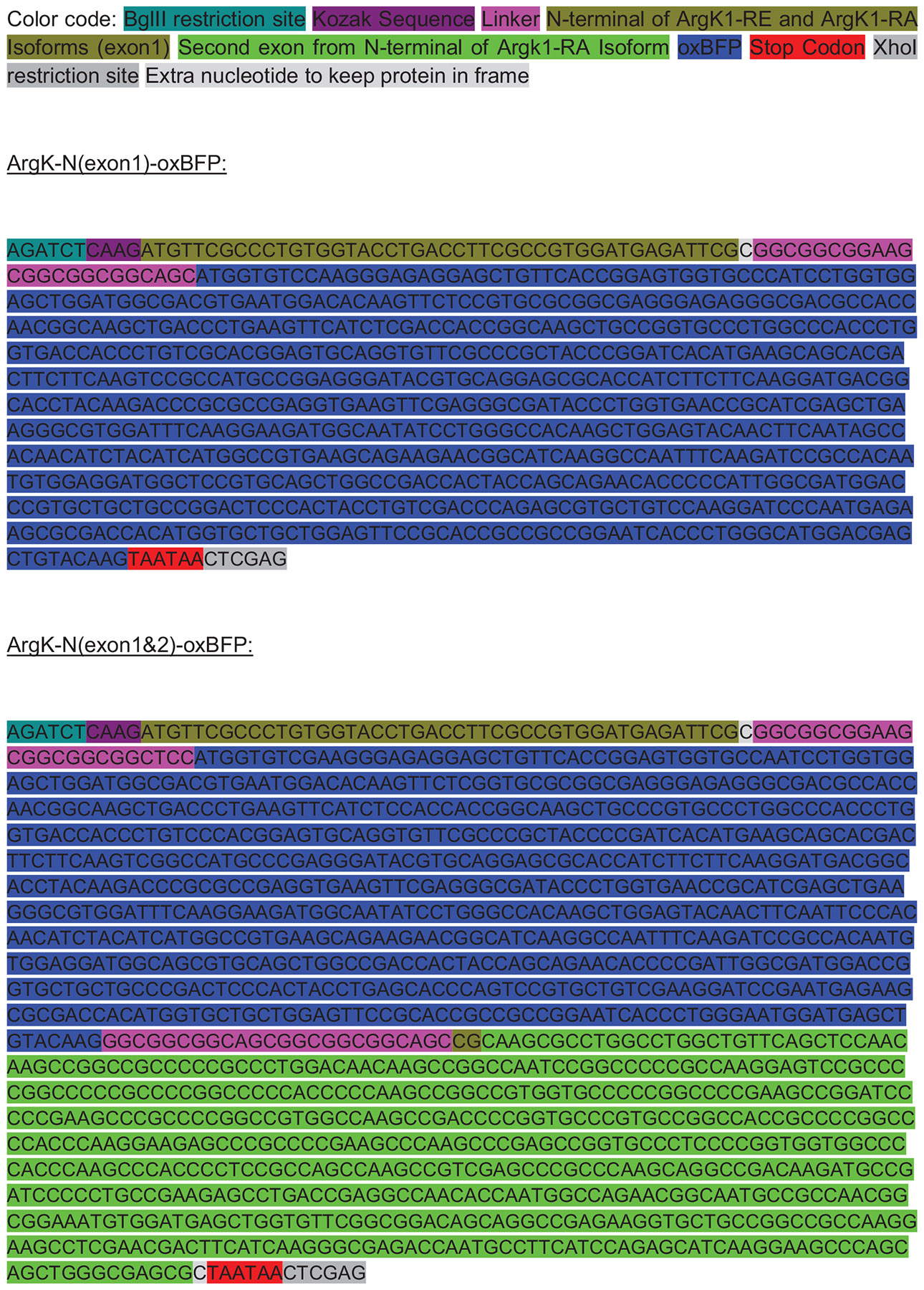
Annotated cDNA sequences for ArgK-N(exon1)-oxBFP (oxidation-resistant BFP) and ArgK-N(exon1&2)-oxBFP constructs prior to cloning into pJFRC14 plasmids and the production of transgenic animals. Colour code: BglII restriction site Kozak Sequence Linker N-terminal of ArgK1-RE and ArgK1-RA Isoforms (exon1) Second exon from N-terminal of Argk1-RA Isoform oxBFP Stop Codon XhoI restriction site Extra nucleotide to keep protein in frame.

UAS-ArgK-N(exon1)-oxBFP and UAS-ArgK-N(exon1&2)-oxBFP cDNA were synthesized by GenScript and codon optimized for *Drosophila melanogaster*. Both synthesized genes contained BglII and XhoI restriction sites and were cloned into pJFRC14 plasmids. The UAS-ArgK-N(exon1)-oxBFP construct was designed to have the first exon from ArgK1-RE and ArgK1-RA isoforms (sequence obtained from FlyBase; 3L: 9064464–9064517) on the N-terminus of oxBFP (Costantini et al., 2015). The UAS-ArgK-N(exon1&2)-oxBFP construct was designed to have the first two exons of the ArgK1-RA isoform (sequence obtained from FlyBase; 3L: 9061907–9062467) flanking oxBFP, with the first exon at the N-terminus and the second exon at the C-terminus (sequence in Fig. A1). Both constructs were injected into *w^1118^* embryos containing the attP2 landing site by Rainbow Transgenic Flies; then transformants were isolated, balanced and outcrossed as described earlier.

#### Targeted genome modification.

ArgK1 endogenously tagged FusionRed lines were generated by GenetiVision (Houston, TX, USA) utilizing the two-step CRISPR/Cas9-catalysed homology-directed repair (HDR) method described by Gratz et al. (2019). The first step replaced part of the endogenous gene with a 3XP3_GFP cassette (which allows easy selection of transformants with eye-specific GFP). The second step replaced the 3XP3-GFP cassette, with a donor plasmid carrying one of two mutated ArgK1 alleles.

**Figure A2.**
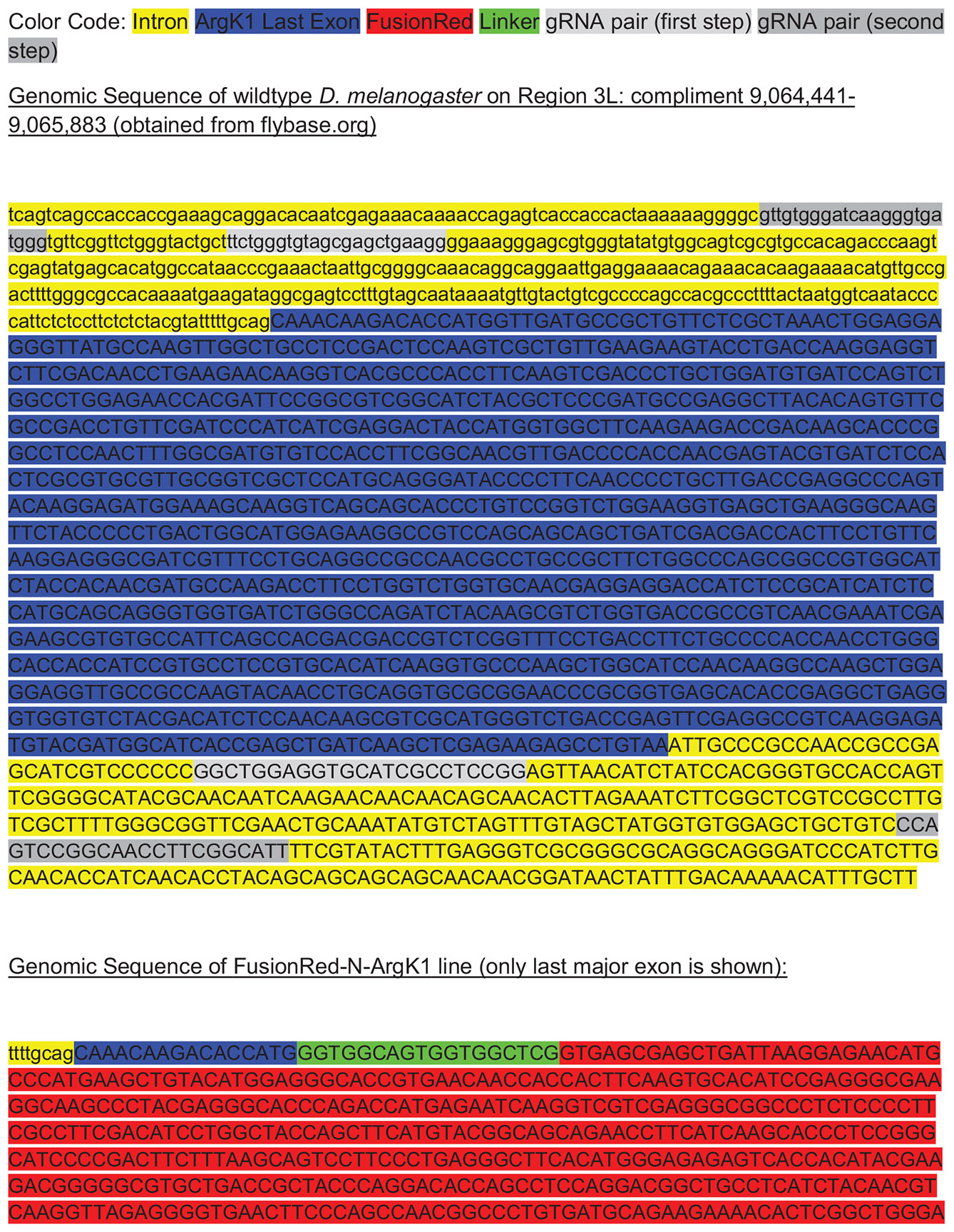

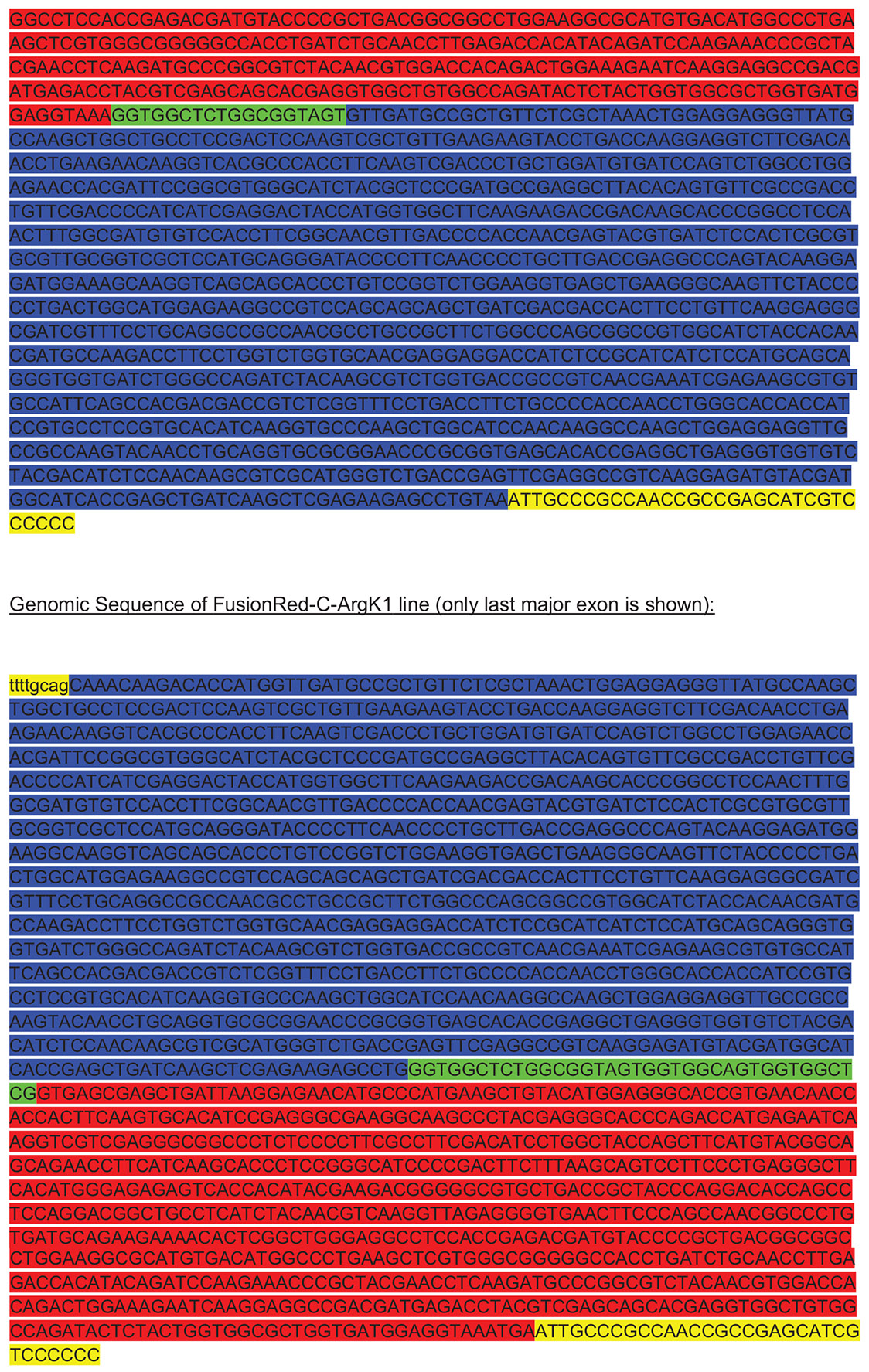
Genomic map sequences for wild-type, FusionRed-N-ArgK1 and FusionRed-C-ArgK1 fly lines (only in region around last major exon): Colour code: Intron ArgK1 Last Exon FusionRed Linker gRNA pair (first step) gRNA pair (second step). background. The average fluorescent intensity for each ROI was measured in the GFP and tdTomato channel. The relative amount of ArgK1 in control *versus* ArgK1 RNAi terminals was assessed by calculating and plotting: [average ROI GFP intensity (terminal) – average ROI GFP intensity (background)]/[average ROI tdTomato intensity (terminal) – average ROI tdTomato intensity (background)] (Fig. S3B). The ArgK1-GFP signal was knocked down by ~98% in both RNAi lines (from 0.0990 ± 0.0435 SD (control) to 0.0025 ± 0.0009 SD (ArgK1 RNAi 35221) and 0.0020 ± 0.0018 SD (ArgK1 RNAi 41697)). *P* = 0.00919 using one-way ANOVA on ranks. Part of panel B reproduced from Fig. 5*L* of Justs et al. (2023).

Step one: nos-Cas9.P embryos (BDSC 54591) were co-injected with a pCFD4 vector (containing gRNA sequences ttctgggtgtagcgagctgaagg and ggctggaggtgcatcgcctccgg) and a donor plasmid. The donor plasmid contained the 3XP3_GFP+ cassette flanked by two 1Kb homologous arms adjacent to a gRNA cleavage site (maps showing the fly genome cleavage sites are shown Fig. A2). The gRNAs were designed to excise the last exon of all ArgK1 isoforms (which is also the catalytic domain), and the cell’s own endogenous HDR mechanism inserted the 3XP3_GFP+ cassette into the fly’s genome. Flies with the GFP eye marker were balanced with TM3. This fly line is an ArgK1 KO line as the catalytic domain is deleted, and it is homozygous lethal.

Step two: embryos of the nos-Cas9.P;ArgK1 KO(3XP3_GFP+ cassette)/TM3 line were co-injected with pCFD4 vector (containing gRNA sequences gttgtgggatcaagggtgatggg and aatgccgaaggttgccggactgg) and donor plasmid. In this second step the donor plasmid contained a mutated ArgK1 allele (last exon of ArgK1 endogenously tagged with FusionRed at either the N-terminal or C-terminal; sequence shown in Fig. A2) flanked by two 1Kb homologous arms adjacent to the gRNA cleavage site. The cell’s own HDR mechanism inserted the mutated allele into the fly’s genome. Individual flies were selected based on loss of GFP eye marker and were balanced with TM3. These lines were then sequenced to determine if the HDR mechanism had integrated ArgK1-FusionRed allele into fly genome. The sequencing results for the FusionRed-N-ArgK1 and FusionRed-C-ArgK1 lines are found in the appendix, confirming that ArgK1 was endogenously tagged by FusionRed on the last exon.

#### KD of ArgK1.

**Figure A3.**
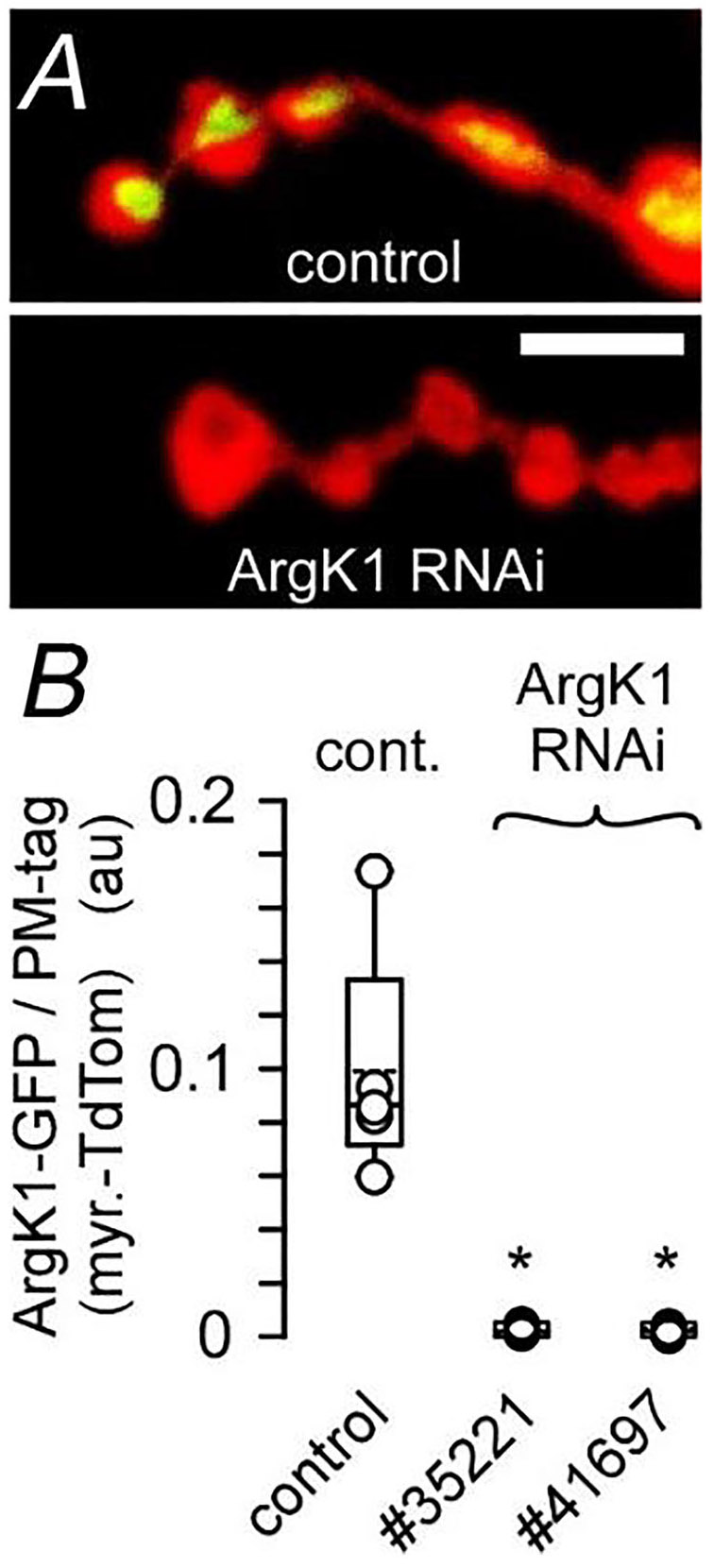
To test the extent to which ArgK1 (arginine kinase 1) RNAi can knock down ArgK1 using the GAL4/UAS system, we expressed two different ArgK1 RNAi transgenes (BDSC (Bloomington Drosophila Stock Centre) 41697 and 35221; both dsRNA) using the OK6-Gal4 driver that drives into both type-Ib and type-Is neurons. A recombined OK6-Gal4;UAS-ArgK1 RNAi stock was created and crossed to a second stock that had been created by recombining UAS-myr-tdTomato (BDSC 32 222) with the endogenously tagged ArgK1 GFP line (BDSC 51522). To create the control line the latter stock above was crossed to OK6-GAL4 without a UAS-ArgK1 RNAi transgene. The inner leaflet of the plasma membrane becomes ‘decorated’ with tdTomato when the fluorophore is myristoylated (Fig. S3A). Larvae were dissected and imaged in Schneider’s insect media. Images of type-Ib motor neuron terminals on muscle fibre 6 (MN6/7-Ib) in segment 4 were obtained on a Nikon A1R confocal laser-scanning microscope using a water-immersion 100×1.10NA objective. Lasers (488 and 561 nm) were used sequentially, and emission filters were optimized for GFP and tdTomato, respectively. The pinhole size was 190 μm, pixel size was 0.04 μm/pixel and Z-stacks of 0.5 μm steps were taken through the entire depth of the terminals for each genotype. Analysis was conducted using ImageJ. A region of interest (ROI) was drawn around the type-Ib terminals (minimum of 10 μm length), and an ROI was drawn in the background. The average fluorescent intensity for each ROI was measured in the GFP and tdTomato channel. The relative amount of ArgK1 in control *versus* ArgK1 RNAi terminals was assessed by calculating and plotting: [average ROI GFP intensity (terminal) – average ROI GFP intensity (background)]/[average ROI tdTomato intensity (terminal) – average ROI tdTomato intensity (background)] (Fig. S3B). The ArgK1-GFP signal was knocked down by ~98% in both RNAi lines (from 0.0990 ± 0.0435 SD (control) to 0.0025 ± 0.0009 SD (ArgK1 RNAi 35221) and 0.0020 ± 0.0018 SD (ArgK1 RNAi 41697)). *P* = 0.00919 using one-way ANOVA on ranks. Part of panel B reproduced from Fig. 5L of Justs et al. (2023).

The effectiveness of ArgK1 KD, through presynaptic expression of a dsRNA (BDSC, 41697) directed against ArgK1 message, was established previously (Justs et al., 2023), showing near-complete KD of the ArgK1-GFP signal (~98% reduction) (*P* = 0.00919, one-way ANOVA; Fig. A3).

### Live imaging of ArgK1-GFP, ArgK1-oxBFP and ArgK1-FusionRed

Images of ArgK1-GFP, ArgK1-oxBFP and ArgK1-FusionRed in female third-instar larval fillet were obtained; the fillet was dissected on a Sylgard tablet in cold haemolymph-like solution 6 (HL6) (Macleod et al., 2002) with 2 mM CaCl_2_. The preparations were then rinsed thrice in 0.1 mm CaCl_2_ HL6 and covered with a glass coverslip; images were obtained using a Nikon 60×, 1.20 NA, Plan Apochromat VC water-immersion objective on a Nikon A1R CLSM fitted with GaAsP detectors. Sequentially scanning was used, starting with the longest laser wavelengths and progressing to the shortest (560, 488, 405nm). Images represent a collapsed Z-series encompassing a limited depth of the ventral ganglion or the full depth of terminal boutons in the periphery (3 and 1 μm step sizes, respectively).

### Metabolite, calcium and SynaptopHluorin imaging

Five different genetically encoded functional probes were used in the presynaptic compartment. ATeam1.03^YEMK^ was used to monitor cytosolic ATP levels ([ATP]_c_), LiLac to monitor lactate levels ([lactate]_c_), Pyronic to monitor pyruvate levels ([pyruvate]_c_), mScar8f to monitor Ca^2+^ levels ([Ca^2+^]_c_) and SynaptopHluorin to monitor exocytosis.

Experiments were conducted on female third-instar larvae. Fillet dissections were performed on a Sylgard (Dow Silicones corp., Midland, MI, USA) tablet in cold HL6 containing 2 mM CaCl_2_ and 7 mM l-glutamic acid (Sigma-Aldrich, St. Louis, MO, USA; to prevent muscle contraction at 80 Hz stimulation; Macleod et al., 2004). Additionally the cerebral lobes were cut from the ventral ganglia, and all nerves connecting the brain to the body muscles were cut, except those to body segment 4. The nerves attached to the ventral ganglion leading to segment 4 were drawn into the lumen of a glass pipette as a loop for subsequent electrical stimulation. Images of type-Ib MN terminals on muscle 13 of body segment 4 were obtained. High-speed fluorescence microscopy was performed using a Nikon Eclipse FN1 microscope fitted with a Nikon 100× water-immersion (1.1 NA) objective. Probes were excited sequentially by a Lumencor SPECTRA-X light source. Emitted light was captured by two Andor iXon3 EMCCD cameras (DU-897) mounted on an Andor TuCam beam splitter (Andor Technology, South Windsor, CT, USA). Filters and dichroic mirrors were obtained from Chroma Technology (Bellows Falls, VT, USA) or Semrock (Lake Forest, IL, USA). ATeam1.03^YEMK^, LiLac with TagRFP-T and Pyronic were all excited by reflection off a triple-edge dichroic mirror (Chroma, 69008bs), with emitted light passing back through the dichroic and a 470/24, 535/30 and 632/60 nm triple-band emission filter (Chroma, 69008bs). mScar8f and SynaptopHluorin were excited by reflection off a quadruple-edge dichroic mirror (Chroma, 89100bs), with emitted light passing back through the dichroic and a 455/50, 525/36, 605/52 and 705 nm/72 nm quad-band emission filter. Nikon Elements software controlled the illumination sequence and camera image acquisition. A Master 8 (AMPI, Israel) controlled the timing of nerve stimulation via an A-M Systems Model 2100 Isolated Pulse Stimulator. Terminals were allowed to equilibrate for at least 2 min after any nerve stimulus train.

To monitor changes in [ATP]_c_ we compared the fluorescence emitted from Cyan Fluorescent Protein (CFP) with mVenus of the ATeam1.03^YEMK^ construct. CFP of ATeam1.03^YEMK^ was excited at 434/17 nm, and emitted light was reflected by a second dichroic mirror (509 nm edge) before being received by a camera through a 475/28 nm filter. ATeam1.03^YEMK^ was also excited by 434/17 nm light, and mVenus emission was received by a second camera after it passed through the second dichroic and a 525/30 nm filter.

To monitor changes in [lactate]_c_ we compared the fluorescence emitted from mTurquoise2 of LiLac with TagRFP-T. LiLac was excited at 436/20 nm, and a camera received the emitted light, whereas TagRFP-T was excited by 510/25 nm light, and its emission was received by the same camera.

To monitor changes in [pyruvate]_c_ we compared the fluorescence emitted from monomeric Teal Fluorescent Protein (mTFP) using Venus, both within the Pyronic construct. mTFP was excited at 434/17 nm, and emitted light was reflected by a second dichroic mirror (509 nm edge) before being received by one of two cameras through a 475/28 nm filter. Pyronic was also excited by 434/17 nm light, and Venus emission was received by the second camera after it passed through the second dichroic and a 525/30 nm filter.

To monitor changes in [Ca^2+^]_c_ we compared the fluorescence emitted from Ca^2+^ -sensitive GCaMP8f with Ca^2+^ -insensitive mScarlet, within the UAS-Syt::mScarlet::GCaMP8f (mScar8f) construct (Fig. 4B). mScarlet fluorescence reports mScar8f expression levels and thus GCaMP8f expression levels; therefore the division of GCaMP8f fluorescence by mScarlet fluorescence removes the confound of expression levels and allows for the comparison of Ca^2+^ levels between different neurons and preparations. GCaMP8f was excited at 483/32 nm, and emitted light was reflected by a second dichroic mirror (580 nm edge) before being received by one of two cameras through a 525/50 nm filter. mScarlet was excited by 550/25 nm light, and its emission was received by the second camera after passing through the second dichroic and a 617/73 nm filter.

For each of ATeam1.03^YEMK^, LiLac and Pyronic, four images pairs were collected every second, yielding four fluorescence ratio estimates. Ten (10) image pairs were collected every second for mScar8f. The ratio of intensities, relative to the ratio prior to nerve stimulation, was plotted against time as Δ*R/R*. The ATeam1.03^YEMK^ fluorescence ratio was corrected for bleaching by fitting a monoexponential to 20 s of pre-stimulus ratio estimates and using this fit to correct the ratio between time −10 and 40 in Fig. 2B (e.g. Macleod, 2012).

SynaptopHluorin was used to monitor changes in exocytosis. It uses synaptic vesicle (SV) membrane protein synaptobrevin to target SuperEcliptic-pHluorin (Sankaranarayanan et al., 2000) to the lumen of SVs (Ng et al., 2002). SynaptopHluorin was the only functional reporter not used in a ratiometric mode. It was excited by 483/32 nm light reflected by a quad-band dichroic mirror, and emitted light passed back through the dichroic and a 525/50 nm emission filter and was received by a single camera. Fluorescence intensity estimates (*F*), relative to the fluorescence prior to nerve stimulation, was plotted against time.

Fluorescence intensity measurements were performed using ImageJ. Movement during imaging was corrected using the Template Matching plugin by aligning consecutive frames. Regions of interest (ROIs) covered non-distal boutons along a 20 μm length of the MN terminal, with two background ROIs for reference. The average pixel intensity values were calculated in each channel for each ROI for each frame and exported to Microsoft Excel. The average of the two background ROIs was subtracted from the average bouton ROI in each channel for each frame. Preparations were not used if the boutons exhibited movement out of frame or out of focus. Outliers were assessed using the median absolute deviation (MAD; Leys et al., 2013) applied to the baseline and delta measurements, where an outlier was any value beyond either the median +3 × MAD or −3 × MAD.

### Electrophysiology

Electrophysiology was exclusively conducted on female third-instar larvae in either haemolymph-like solution 3 (HL3; Stewart et al., 1994) or HL6 containing MgCl_2_ and CaCl_2_ as described in Fig. 3. The fillet was dissected in cold HL6 on Sylgard tablets, and recordings were made 20–60 min after the segmental nerves were transected. Signals were detected, digitized and recorded using an Axoclamp 900A amplifier (Molecular Devices, Sunnyvale, CA, USA) connected to a 4/35 PowerLab (ADInstruments, Colorado Springs, CO, USA) and a PC running LabChart version 8.0. Micropipettes were filled with a mixture of 3 m KCl and 3 m K-acetate (1:1). Measurements were performed on segment 4 under a 20× water-dipping objective of a BX50WI Olympus microscope to allow unequivocal identification of muscle fibres. A two-electrode voltage clamp (TEVC, clamped to –70 mV) was used to quantify the combined release from MN6/7-Ib and MNSNb/d-Is onto body-wall muscle fibre 6. A suction pipette applied 0.3 ms electrical impulses to the transected nerve to evoke release from both MN terminals. The voltage used was 20%–50% above the threshold needed to initiate an action potential (AP) in both MNs. Preparations were discarded if we observed a failure to evoke release from both MNs in response to any AP in a stimulus train or if the unclamped resting membrane potential (RMP) decreased below –55 mV. Where quantal content (QC) was calculated (Fig. 3H and N), a minimum of 10 excitatory junctional potentials (EJPs) were recorded during a 0.2 Hz stimulation, along with 30 miniature EJPs (mEJPs), prior to implementing a TEVC. QC was calculated by dividing the corrected mean EJP amplitude by the mean mEJP amplitude. Mean EJP amplitude, but not mean mEJP amplitude, was corrected for non-linear summation (McLachlan and Martin, 1981). An outlier was any values beyond either the median +3 × MAD or −3 × MAD.

### *In vivo* MN performance assay

We have described the assembly of the apparatus to evoke body-wall contractions in *Drosophila* larvae, preparation of larvae for this assay and data analysis in a separate ‘Methods’ article (Arab et al., 2025). Briefly we expressed a light-activated opsin (H134R-ChR2) in MNs using OK371-GAL4, raised the larvae in the dark on food supplemented with all-trans-retinal (ATR; Sigma-Aldrich, St. Louis, MO) (500 μM ATR and 0.5% ethanol, final concentration) and drove musculoskeletal contractions using blue light in an illumination cycle of 2 s on and 1 s off. Images of the larvae were obtained using a CCD camera at a rate of 10 frames per second, and custom-built software calculated the larval perimeter in each frame (offline).

### Simulation of ATP dynamics

The simulation model is described in Justs et al. (2023). The only differences are the timing of APs, described in the main text, and the initial values of [P_i_] and [ADP], discussed in the ‘Parameter values’ section. The model tracks the concentrations of six metabolites ([ATP], [ADP], [AMP], [ArgP], [Arg] and [P_i_]) that change according to three processes: ATP consumption, ATP production and phosphagen equilibration.

#### ATP consumption.

ATP consumption is represented by the time rate of [ATP] reduction, in exchange for [ADP] + [P_i_]:

(1) $$\text{Consumption}\;:\;[\text{ATP}]\to [\text{ADP}]+[P_{i}]$$

AP-associated ATP consumption processes (signalling processes) are modelled as an instantaneous spike, followed by an exponential decay over the time associated with each of the ATP consumption processes; Na^+^ extrusion, Ca^2+^ extrusion and SV recycling/refilling are associated with neurotransmitter (NT) release. That is each process j will consume $N_{j}$ ATP molecules, decay with a time constant $\tau_{j}$ and manifest a spike of height $N_{j}∕\tau_{j}$ at the time of firing. Meanwhile a static base rate of ATP consumption due to non-signalling activities, unrelated to APs, is applied throughout the time of simulation. The time of evolution of ATP consumption from a single AP occurring at time $t_{0}$ is then

Consumption=base rate for t<t0

$\text{Consumption}=\text{base rate}+\sum_{j\in \{{\text{Na}}^{+},\;{\text{Ca}}^{2+},\;NT_{1},\;NT_{2},\;NT_{3}\}}$

(2) $$\frac{N_{j}}{\tau_{j}}\;e^{\frac{t_{0}-t}{\tau_{j}}}\;\text{for}\;t\geq t_{0}$$

During a train with a firing rate (FR, Hz), a new spike occurs every 1/FR seconds. Each new spike summates upon the transients of previous spikes. A *full cycle* is the span of time from the beginning of one train of spikes to the beginning of the next train, which corresponds to a contraction cycle during locomotion. The *duty cycle* is the amount of time during a *full cycle* when a train is firing. For example for a train of APs firing at 10 Hz, starting at $t=t_{0}$, with a *full cycle* of 1 s and a *duty cycle* of 0.6 s, seven spikes will occur, 100 ms apart, from time $t_{0}$ to $t_{0}+0.6\;s$. The remaining time of the full cycle will not incur another spike, and another train with the same parameters will start at $t_{0}+1\;s$.

#### ATP production.

ATP production is represented by the time rate of the decrease in [ADP] and [P_i_] in exchange for an increase in [ATP]:

(3) $$\text{Production}\;:\;[\text{ADP}]+[P_{i}]\to [\text{ATP}]$$

As in Justs et al. (2023), we first compute the energy state (ES) = [ATP]/([ADP][P_i_]). Then we use the model and parameters described in Wilson (2017) to compute the cytochrome c turnover rate. The ATP production rate is equal to this turnover rate times the cytochrome c concentration. The latter is inferred by requiring the values of [ADP], [P_i_] and [ATP] found in the literature (see the section ‘Parameter values’) to yield an ATP production rate equal to the terminal’s rest consumption rate:

(4) $$p\;(\text{ES})=(\text{base rate})\frac{p_{\text{cc}}\;(\text{ES})}{p_{\text{cc}}\;(\text{base ES})}$$

where p is the ATP production rate, $p_{\text{cc}}$ is the cytochrome c turnover rate, ‘base rate’ is the rest-level ATP consumption rate and (base ES) is the value of the ES computed from the [ADP], [P_i_] and [ATP] values from the literature. Finally we impose a maximum ATP production as follows:

(5) $$p_{\text{capped}}={\left(p^{-5}+{\left(\text{max rate}\right)}^{-5}\right)}^{-\frac{1}{5}}$$

where p is the uncapped rate from eqn (4). This ensures a smooth transition from the Wilson model from when the inverse ES is small to (max rate) when the ES is large. The value of (max rate) is discussed in the main text.

#### Equilibration.

Adenylate kinase (AK) and ArgK maintain the following equilibria at all times:

Equilibration:

(6) AMP+ATP↔2ADP,ADP+ArgP↔ATP+Arg

#### Numerical integration.

At each time step [ADP] and [ATP] are updated first using the production and consumption rates. Then [AMP], [ADP], [ATP], [Arg] and [ArgP] are updated according to eqn (6) until their equilibrium relations are satisfied:

(7) $$K_{\text{Ad}}[\text{ATP}]\;[\text{AMP}]={\left[\text{ADP}\right]}^{2},\;K_{\text{Ph}}[\text{ADP}]\;[\text{Arg}P]=[\text{ATP}]\;[\text{Arg}]$$

where $K_{\text{Ad}}$ and $K_{\text{Ph}}$ are the equilibrium constants for AK and ArgK, respectively. When simulation is done without phosphagen, the second equation in eqn (7) is not enforced.

#### Parameter values.

Following Justs et al. (2023) we use the most complete set of model parameter values from the nervous system of a single invertebrate we know of: the giant squid axon. Initial concentrations are set to 2.16 mm [ATP] (Caldwell, 1960; Mullins & Brinley, 1967; Requena et al., 1979), 7.5 mm [ArgP] (DiPolo & Beauge, 1996) and 3.3 mm [Arg] (Deffner, 1961; Deffner & Hafter, 1959). *K*_Ph_ is set to 39.6 (Teague & Dobson, 1999). *K*_Ad_ is set to 1 (Wilson, 2017). [AMP] and [ADP] initial concentrations are derived from eqn (7) using the aforementioned values for [ATP], [ArgP], [Arg], *K*_Ph_ and *K*_Ad_. Squid axon [P_i_] measurements range from 3.8 to 17.8 mm (Caldwell, 1960; Deffner, 1961). To ensure the fairest comparison between systems with and without a phosphagen system, the resulting values (24.2 μ_m_ [ADP] and 0.27 μ_m_ [AMP]) are used irrespective of whether a phosphagen system is present. Because ionic concentrations in terrestrial insect nerve tissue are uniformly lower than those in their marine invertebrate counterparts (*Periplaneta* and *Romalea*; Potts and Parry, 1964), we now use the lower end of the squid axon range: [P_i_] = 3.8 mM. This choice is further supported by comparison with the terrestrial *Manduca Sexta P* concentrations, where tissue [P_i_] ranges from 1.2 to 3.8 mm (Woods et al., 2002).

#### Implementation.

We implemented the aforementioned model using the Python programming language. The full simulation code is available at https://github.com/yffily/paper_phosphagen2/.

### Statistical analysis

Tests were performed using SigmaStat version 3.5, and each test is described where used. Student’s *t* tests were performed to determine the significance in individual comparisons. For multiple comparisons ANOVA was applied, and an overall *α* of 0.05 was required to claim significance. ANOVAs were run on ranks when tests for data normalcy failed. *N* represents the number of preparations, or larvae, throughout as data from no more than one NMJ or muscle fibre were collected in any preparation.

## Results

### A small portion of ArgK1 localizes to presynaptic mitochondria via an N-terminal signal sequence

**Figure 1.**
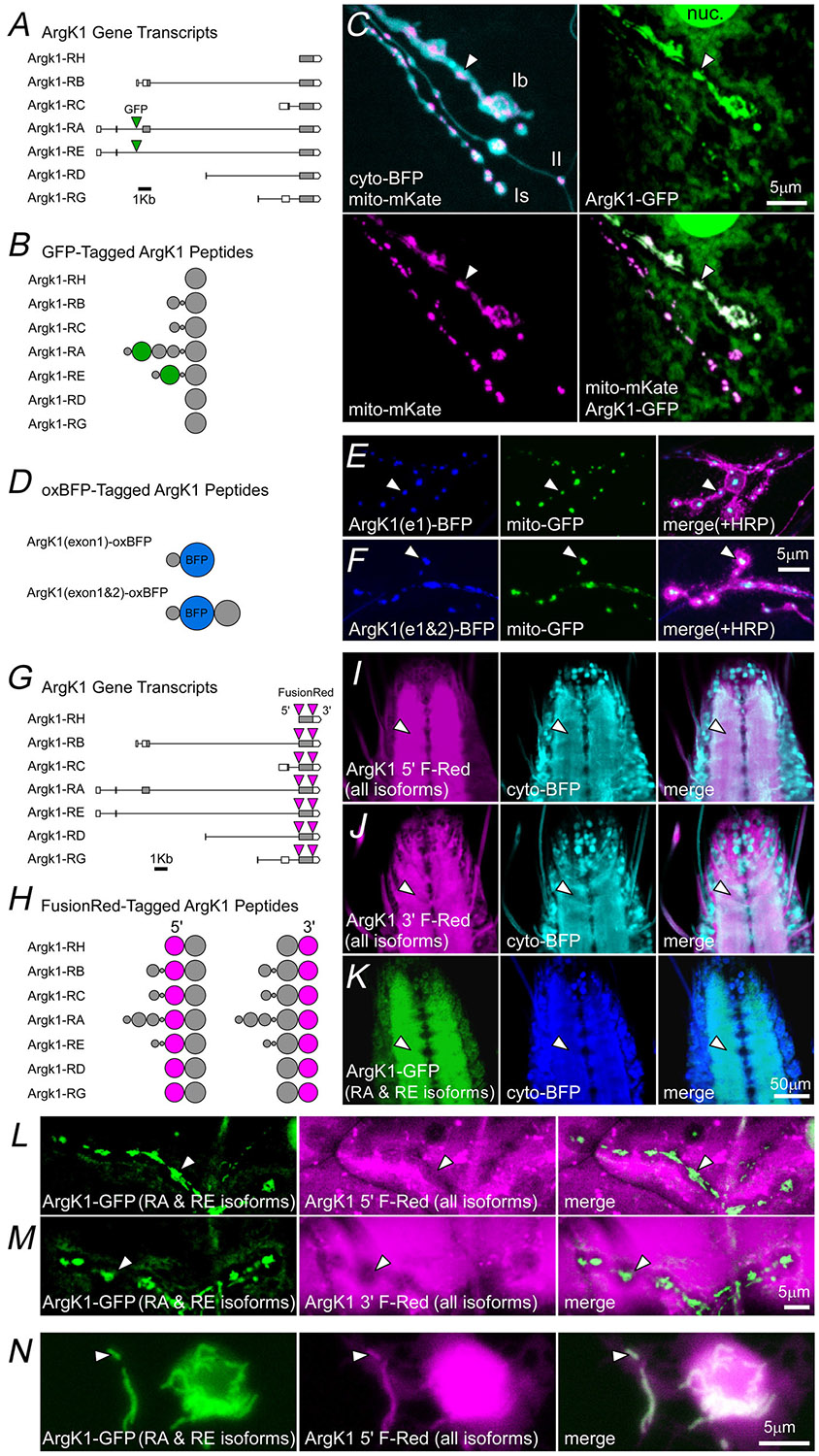
ArgK1 (arginine kinase 1) is targeted to presynaptic mitochondria by an N-terminal sequence *A*, predicted splice isoforms of ArgK1 (FlyBase) showing intronic sites of transposon-mediated GFP (green fluorescent protein) DNA insertion via MiMIC cassette. Seven unique mRNA transcripts have been identified and annotated; for example Argk1-RH refers to splice isoform RH. *B*, representation of the proteins generated from ArgK1 splice isoforms, where the volume of each ‘sphere’ represents the relative length of peptide corresponding to each exon. Green spheres represent the likely inclusion of GFP in the ArgK1 polypeptide behind exon 1. *C*, an image of a short confocal stack projection through live motor neuron (MN) terminals of MN13-Ib and MNSNb/d-Is and MNSNb/d-II on muscle fibre 13, demonstrating the relative localization of ArgK1-GFP (1 copy) and neuronal expression of mitochondrial-targeted mKate and cytosolic TagBFP (Tag Blue Fluorescent Protein). *D*, representation of the proteins generated from two different transgenic constructs; ArgK1(exon1)-oxBFP (oxidation-resistant BFP), where oxBFP DNA was C-terminally appended to DNA coding the first ArgK1 exon, and ArgK1(exon1)-oxBFP where oxBFP was flanked by DNA of the first two exons of ArgK1. *E*, an image (single confocal plane) of live MN terminals showing ArgK1(exon1)-oxBFP localization relative to neuronal expression of mitochondrial-targeted GFP (mito-GFP) and a Cy3-conjugated HRP antibody (HRP-Cy3). *F*, an image (single confocal plane) of live MN terminals showing ArgK1(exon1&2)-oxBFP localization relative to mito-GFP and an HRP-Cy3 antibody. *G*, predicted splice isoforms of ArgK1 showing FusionRed DNA insertion sites (5′ or 3′) in two different CRISPR/Cas9-edited fly lines. One fly line contains FusionRed DNA inserted at the N-terminus of the last exon (ArgK1 5′ FusionRed), whereas the other has FusionRed DNA inserted at the C-terminus of the last exon (ArgK1 3′ FusionRed). *H*, representation of the proteins generated in each of the two fly lines (ArgK1 5′ FusionRed and ArgK1 3′ FusionRed) with endogenously-tagged ArgK1. *I–M*, in panels I, L and N, FusionRed was inserted in the 5′ position of the catalytic subunit in all isoforms (corresponding to the first column in panel H), whereas in panels J and M FusionRed was inserted in the 3′ position (corresponding to the second column). *I*, an image (single confocal plane) of the live ventral ganglion (VG) showing the neuropil localization of ArgK1 5′ FusionRed relative to pan-neuronal expression of cytosolic TagBFP. *J*, an image of the VG showing ArgK1 3′ FusionRed relative to cytosolic TagBFP. *K*, an image of the VG showing ArgK1-GFP expression relative to cytosolic TagBFP. *L*, an image of a short confocal stack projection through live terminals of MN13-Ib and MNSNb/d-Is, showing a strong presence of ArgK1 5′ FusionRed (one copy) in muscle fibre 13, but little sign of FusionRed in the mitochondria of MN terminals revealed by the presence ArgK1-GFP (one copy). *M*, As in panel L, but in the ArgK1 3′ FusionRed line. *N*, ArgK1 5′ FusionRed colocalized with ArgK1-GFP revealed mitochondria (e.g. arrowhead) in live neurons cultured from the VG of larvae bearing one copy of the ArgK1 5′ FusionRed-edited allele and one copy of the ArgK1-GFP allele (as in panel L). Arrowheads provide registration points in each image panel. nSyb-GAL4 was used to drive the expression of transgenes in panels C–K. All fluorophores in panels L–N result from editing the ArgK1 endogenous locus.

In *Drosophila* ArgK1 is alternatively spliced, and transposon-mediated insertion of GFP DNA into an intron of ArgK1 via MiMIC cassette (stock 51522, BDSC; Fig. 1A) revealed that at least one ArgK1 isoform targets mitochondria (either RA or both RA and RE, Fig. 1B and C; Justs et al., 2023). To test whether one or both N-terminal exons are required for mitochondrial targeting, we produced two transgenic animals: one in which oxBFP DNA was C-terminally appended to cDNA coding the first exon and one in which oxBFP was flanked by cDNA of the first two exons (Fig. 1D). Both UAS constructs targeted the fluorophores to mitochondria, demonstrating that the first exon was sufficient (Fig. 1D–F). Unexpectedly the first exon in the absence of the second resulted in mitochondria that appeared to ‘round up’ (Fig. 1E), indicating a dominant-negative effect of the exogenous first exon peptide (MFALWYLTFAVDEIRK). This analysis indicated that splice isoforms RA and RE of ArgK1 (those including the first exon) localize to mitochondria.

To reveal the expression pattern of all ArgK1 splice isoforms, we used the CRISPR/Cas9 gene editing technique to insert FusionRed DNA at either the 5′ or 3′ end of the catalytic portion of ArgK1 (Fig. 1G and H). FusionRed revealed an endogenous ArgK1 expression pattern that filled the neuropil of the larval ventral ganglion (Fig. 1I and J), similar to the expression pattern of ArgK1-GFP (Fig. 1K). However the expression pattern was difficult to interpret by looking at the NMJ as FusionRed in the muscle overwhelmed any FusionRed that might have been detected in MN terminals (Fig. 1L and M). To determine whether FusionRed also localized to mitochondria, we examined primary cells cultured from larval ventral ganglia. Here we found that neurites containing mitochondria did not exclude FusionRed at the site of mitochondria, indicating that FusionRed-tagged isoforms of endogenous ArgK1 were not just in the cytosol but also in mitochondria, as expected (Fig. 1N, arrowheads).

### ArgK1 KD leads to deficits in presynaptic energy metabolism

Our functional analysis of MNs focused on those with type-Ib ‘big’ terminal boutons that innervate all body-wall muscle fibres and which drive contractions with high-frequency bursts. A second, less well-studied, MN with type-Is ‘small’ terminal boutons innervates most of the same muscle fibres (Hoang & Chiba, 2001). Type-Is terminals fire more intermittently than type-Ib MNs, at a lower frequency (Chouhan et al., 2010; Lu et al., 2016), and impose a lower power demand serviced by a lower mitochondrial volume density (Justs et al., 2022, 2023). Although functional differentiation also occurs between type-Ib terminals examined here (MN13-Ib and MN6/7-Ib; Chouhan et al., 2012; Justs et al., 2022), our experimental protocols were designed to challenge the specific MN being investigated relative to its endogenous firing frequency to allow subsequent comparisons between data sets.

**Figure 2.**
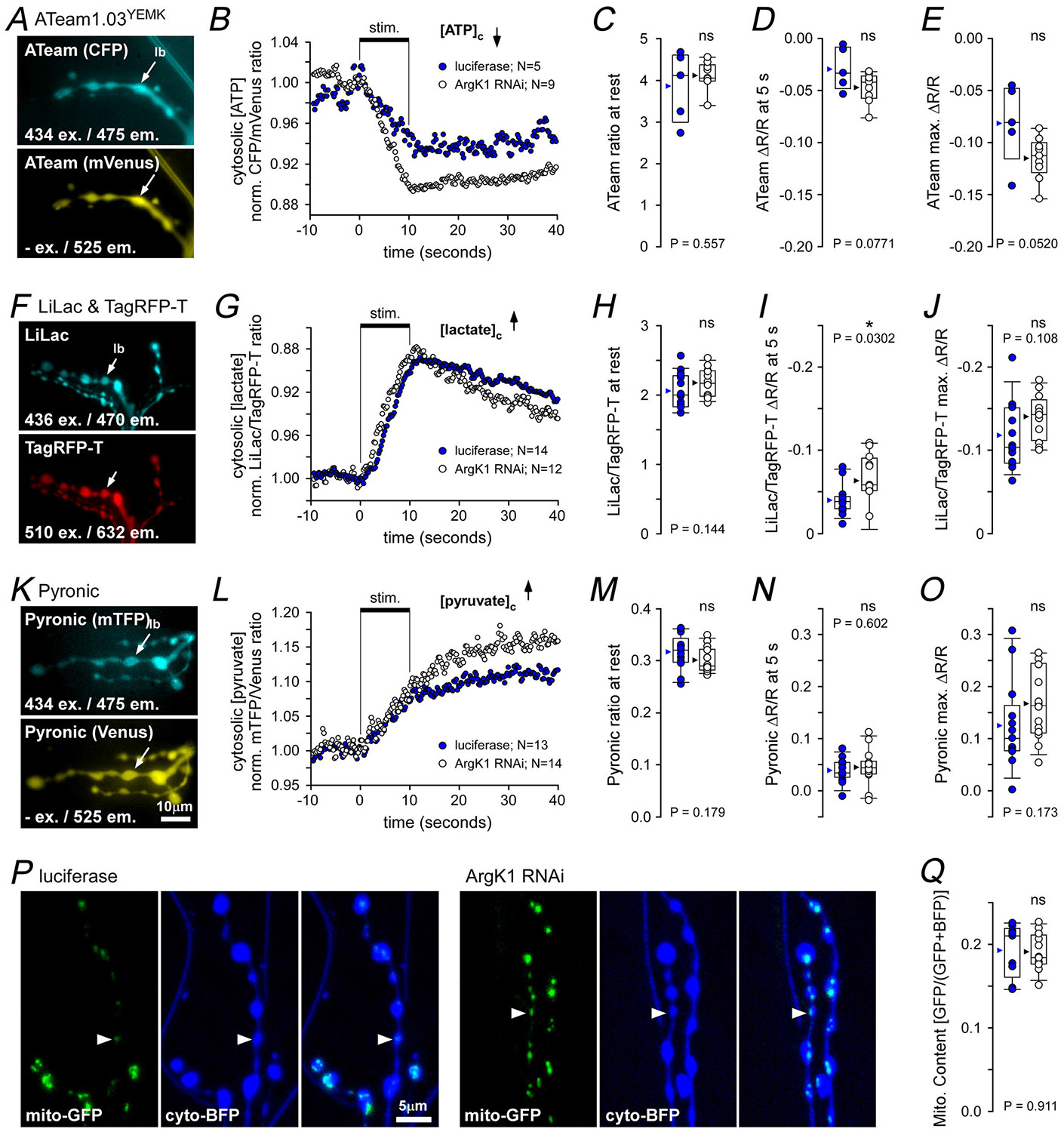
Presynaptic metabolic imaging and mitochondrial content after ArgK1 (arginine kinase 1) KD (knock-down) *A*, images of motor neuron (MN) terminals on muscle fibre 13 expressing ATP reporter ATeam1.03^YEMK^ captured prior to stimulation using excitation and emission wavelengths as shown (mVenus is excited by CFP emission). Pan-neuronal nSyb-GAL4 was used to drive UAS-ATeam1.03^YEMK^ expression along with either the UAS-ArgK1 dsRNA (41697) RNAi line, to knock down ArgK1 (*N* = 9), or the UAS-luciferase as a control (*N* = 5). *B*, a plot showing the normalized average ATeam1.03^YEMK^ fluorescence ratio measured from MN13-Ib terminals only while stimulated at 80 Hz for 10 s, as shown. A ratio decrease is interpreted as a decrease in the cytosolic ATP concentration ([ATP]_c_). *C*, box plots of ATeam1.03^YEMK^ ratio prior to nerve stimulation (at rest) for each preparation. *D*, change in ATeam1.03^YEMK^ ratio (ΔR/R_rest_) 5 s after start of stimulus train. *E*, greatest change in the ATeam1.03^YEMK^ ratio (max ΔR/R_rest_); minimum detected between 8 and 12 s. *F*, images of MN terminals expressing lactate reporter LiLac::TagRFP-T captured prior to nerve stimulation. nSyb-GAL4 drove the expression of UAS-LiLac::TagRFP-T along with either UAS-ArgK1 dsRNA (*N* = 12) or UAS-luciferase (*N* = 14). *G*, the normalized average LiLac-to-TagRPF-T ratio shown responding to nerve stimulation. Scale is inverted, and lower numbers are interpreted as higher concentrations of lactate ([lactate]_c_). *H*, LiLac-to-TagRFP-T ratio at rest. *I*, change in LiLac-to-TagRFP-T ratio (ΔR/R_rest_) 5 s after start of stimulus train. *J*, greatest change in the LiLac-to-TagRFP-T ratio (max ΔR/R_rest_); maximum detected between 8 and 12 s. *K*, images of MN terminals expressing pyruvate reporter Pyronic, captured prior to stimulation. Venus is excited by mTFP emission. Scale bar applies to images in panels A, F and K. nSyb-GAL4 drove the expression of UAS-Pyronic along with either UAS-ArgK1 dsRNA (*N* = 14) or UAS-luciferase (*N* = 13). *L*, the normalized average Pyronic ratio shown responding to nerve stimulation. An increase in the ratio is interpreted as an increase in pyruvate concentration ([pyruvate]_c_). *M*, pyronic ratio at rest. *N*, change in Pyronic ratio (ΔR/R_rest_) 5 s after start of stimulus train. *O*, greatest change in the Pyronic ratio (max ΔR/R_rest_); measured at 40 s. All metabolic imaging was performed on the terminal of MN13-Ib in segment 4. Average traces shown. A three-point moving average was used on ATeam1.03^YEMK^ and Pyronic traces. ATeam1.03^YEMK^ fluorescence ratio (but not LiLac or Pyronic) corrected for bleaching as described in the ‘Methods’ section. Metabolic imaging experiments were discarded as outliers if the initial ratio of ΔR/R was beyond 3 × median absolute deviations of the median in panels C–E, H–J and M–O. Arrows and arrowheads provide registration points in image panels. The numbers of independent preparations (larvae; N) are shown in panels B, G and L. *P*, images from short confocal stack projection through live terminals on muscle fibre 6. OK6-GAL4 was used to drive UAS-mito-GFP and UAS-cyto-BFP expression in MNs, along with either UAS-ArgK1 dsRNA to knock down ArgK1 (*N* = 12) or UAS-luciferase (*N* = 9). *Q*, box plots of mitochondrial content of MN6/7-Ib terminals. Mitochondrial content was quantified as the amount of mito-GFP fluorescence divided by the sum of mito-GFP and cyto-BFP fluorescence. Units are arbitrary. Box plots show values from all preparations; mean (arrowhead), median (line), 25–75 percentile box and 10%–90% whiskers. B–E, G–J, L–O and Q: black symbols used for ArgK1 RNAi, blue for control. Mann–Whitney rank-sum test in panels C and H; unpaired Student’s *t* tests used on all other two-sample comparisons.

Using a fluorescent reporter of the cytosolic ATP-to-ADP ratio (Perceval high range (PercevalHR)), we previously demonstrated that ATP-to-ADP levels decrease further in ArgK1 KD terminals than in controls under intense metabolic load (Justs et al., 2023). Here, as previously, we used an RNAi approach to knock down the neuronal expression of ArgK1 by expressing a dsRNA (BDSC 41697) that reduces the expression of GFP-tagged ArgK1 isoforms by ~98% (Fig. A3). We followed up on our previous finding using a different reporter of adenine nucleotides, as PercevalHR is pH sensitive, and these MN terminals acidify under load (Rossano et al., 2013). We adopted the use of ATeam1.03^YEMK^ (Imamura et al., 2009), a reporter of ATP levels less susceptible to pH change near neutral, with an intracellular dissociation constant (*K*_D_) of ~2.7 mm at room temperature *in situ* (Lerchundi et al., 2020). As in our previous study we examined the terminals of MN13-Ib, in preference to MN6/7-Ib, on muscle 6, as the larger boutons of MN13-Ib provide a more robust signal for dynamic fluorescence imaging. To impose a substantial metabolic load, hemisegment nerves containing MN axons were stimulated for 10 s at 80 Hz; a frequency nearly double the firing frequency of MN13-Ib recorded during fictive locomotion (42 Hz; Chouhan et al., 2010). The presence of the physiological levels of extracellular Ca^2+^ requires the physiological levels of Ca^2+^ pumping and NT release, but muscle contraction can be stifled by adding glutamate (7 mm) to desensitize postsynaptic glutamate receptors (Macleod et al., 2004). ATeam1.03^YEMK^ revealed a rapid reduction in ATP levels in control terminals during nerve stimulation, and ATP levels fell by an even greater extent in ArgK1 KD terminals (Fig. 2A–E; Table 1), but the impact of ArgK1 KD did not reach statistical significance (*P* = 0.052). An important consideration for interpreting ATP level data is that the ATP levels measured here are expected to decrease less sharply than the ATP-to-ADP ratio reported previously (Justs et al., 2023).

Previously we found that lactate levels increase at a faster rate in ArgK1 KD terminals than in controls under an intense metabolic load, and we speculated that this is a result of either a compensatory increase in glycolysis or a reduced mitochondrial ability to metabolize pyruvate, leading to a faster rate of pyruvate conversion to lactate (Justs et al., 2023). Here we used an improved fluorescent cytosolic reporter of lactate (LiLac; Koveal et al., 2020) to re-examine changes in lactate levels (Fig. 2F–J; Table 1), and we used cytosolic pyruvate reporter Pyronic (Gonzalez-Gutierrez et al., 2020; San Martin et al., 2014) to perform complementary measurements of presynaptic pyruvate levels (Fig. 2K–O). LiLac measurements, like previous Laconic measurements, revealed faster rates of lactate accumulation in ArgK1 KD terminals than in controls (*P* = 0.0302) (Fig. 2I; Table 1), and although Pyronic suggested that pyruvate levels exceeded control levels after nerve stimulation, it did not reach statistical significance (*P* = 0.173) (Fig. 2O; Table 1). An increase in pyruvate would be consistent with mitochondria being unable to keep pace with the rate of pyruvate production in ArgK1 KD terminals, but ultimately, the data did not provide the statistical basis for rejecting the alternate hypothesis that there was a compensatory increase in glycolysis and pyruvate production.

In mammals KD of various isoforms of CK leads to an increase in the glycogen content and glycolytic capacity of skeletal muscle fibres, as well as an increase in mitochondrial content and the expression and activity of respiration chain proteins (de Groof et al., 2001; Dzeja et al., 2011; Kaasik et al., 2003; Steeghs, Benders, et al., 1997; Veksler et al., 1995). MN6/7-Ib terminals on muscle fibre 6 were examined for an increase in mitochondrial content in response to ArgK1 KD. These terminals are very similar to other terminals with type-Ib terminals, such as MN13-Ib with a high energy demand and a high mitochondrial volume density (MN6/7-Ib: 6.29%, MN13-Ib: 6.42%; Justs et al., 2022). Fluorescence microscopy, although poorly suited for making quantitative estimates of mitochondrial content, is well suited for estimating *differences* or *changes* in content. Our measure of content was calculated as the mitochondrial fluorescence intensity (mito-GFP) divided by the sum of mitochondrial and cytosolic fluorescence intensity (mito-GFP and cyto-BFP) (Fig. 2P and Q). ArgK1 KD did not result in a significant change in the mitochondrial content reported by this ratio, irrespective of whether fluorophore expression was driven by OK6-GAL4 (*P* = 0.911; Fig. 2Q; Table 1) or a stronger pan-neuronal driver (nSyb-GAL4, *P* = 0.577; data not shown).

### Deficits in neurotransmission are revealed by burst firing after ArgK1 KD

**Figure 3.**
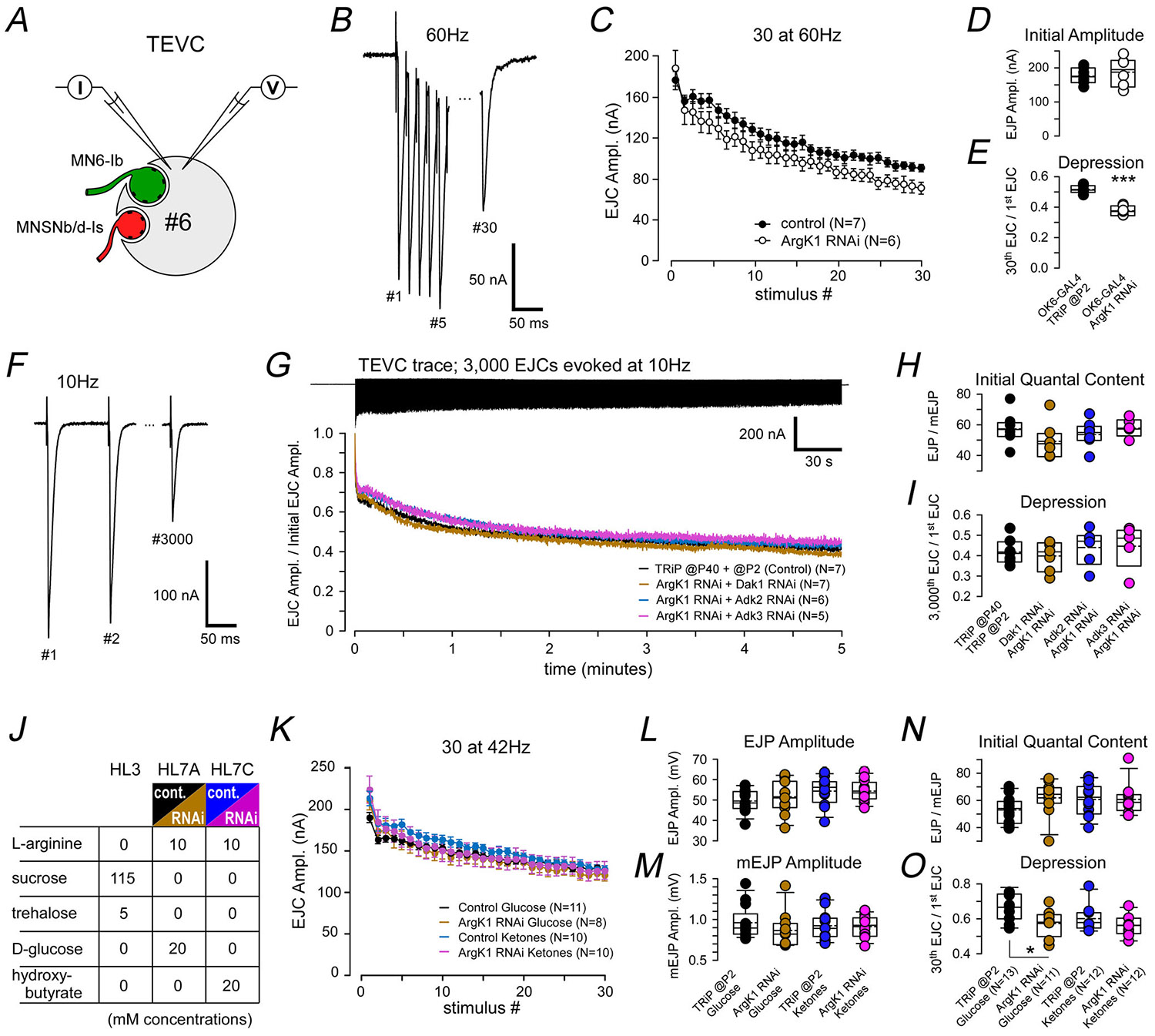
Electrophysiological analysis of the impact of ArgK1 (arginine kinase 1) KD (knock-down) on neurotransmission *A*, a diagram of a transverse section through muscle fibre 6, innervated by motor neurons (MNs) MN6/7-Ib (green) and MNSNb/d-Is (red), and subject to a two-electrode voltage clamp (TEVC) by current-passing (*I*) and voltage-monitoring (*V*) electrodes. *B*, example trace showing compound excitatory junctional currents (EJCs). The hemisegment nerve was stimulated at a supramaximal rate (60 Hz) well above their individual endogenous firing rates. *C*, plots of average compound EJC amplitude when the nerve was stimulated for 500 ms. ArgK1 expression was knocked down in MNs by OK6-GAL4-driven expression of the UAS-ArgK1 dsRNA (41697) RNAi line (*N* = 6), with OK6-GAL4 driving P{CaryP}attP2 (BL36303) as a control (*N* = 7). Recordings in panels B–E were conducted in HL3 (hemolymph-like solution 6) saline; 3 mm [Ca^2+^] and 20 mm [Mg^2+^]. Experiments were discarded as outliers if the initial amplitude or depression was beyond 3 × median absolute deviations of the median. *D*, plots of compound EJC amplitude in response to the first stimulus in the train in panel C. Student’s *t* test (not significant, *P* = 0.537). *E*, depression ratio box plots (ratio of amplitudes between the 30th and 1st EJC). Student’s *t* test (***P < 0.001). *F*, example trace showing compound EJCs in muscle 6 while stimulating the hemisegment nerve at 10 Hz. *G*, top, example trace showing compound EJCs in muscle 6 while stimulating the nerve at 10 Hz for 5 min (3000 impulses). Bottom, plots of average EJC amplitude normalized to the amplitude of the first EJC in each train. In each case ArgK1 expression was knocked down by nSyb-GAL4-driven expression of ArgK1 RNAi in the MNs, combined with dsRNA for knock-down of Dak1 (*N* = 7), Adk2 (*N* = 6) or Adk3 (*N* = 5). As a control nSyb-GAL4 drove both P{CaryP}attP40 (BL36304) and P{CaryP}attP2 (BL36303) (*N* = 7). Recordings in panels F–I were conducted in HL6 saline to best maintain the preparation during prolonged stimulation; 2 mm [Ca^2+^] and 15 mm [Mg^2+^]. *H*, single-electrode current-clamp recordings determined the quantal content (QC) of release evoked from both terminals, prior to the insertion of a second electrode to implement a TEVC. Box plots of the average QC, calculated by dividing the average corrected EJP (excitatory junctional potential) amplitude (≥10 EJPs evoked at 0.2 Hz) by the average amplitude of ≥30 miniature EJPs (mEJPs). *I*, depression ratio box plots (ratio of amplitudes between the 3000th and 1st EJC). No significant differences were found using one-way ANOVA applied in panel H (*P* = 0.336) or panel I (*P* = 0.725). *J*, a table highlighting differences in salines used to test the susceptibility of neurotransmission to metabolic substrates that reduce the opportunity for glycolytic compensation. l-Arginine was increased to accommodate the possibility of a greater ArgK1 substrate requirement for ATP buffering. *K*, average compound EJC amplitude when the nerve was stimulated at 42 Hz for 700 ms (30 impulses). The stimulus duration reflects a combination of the typical firing periods for MN6/7-Ib (830 ms) and MNSNb/d-Is (210 ms) during a 1-s peristaltic contraction (Justs et al., 2022). ArgK1 expression was knocked down by nSyb-GAL4-driven expression of ArgK1 RNAi, with P{CaryP}attP2 (BL36303) driven as a control. Values of *N* shown on plot. Recordings were conducted in one of the salines listed in panel J, each a derivative of HL3 (hemolymph-like solution 3) that also included (in mM) 5 KCl, 80 NaCl, 16 NaHCO_3_, 2 CaCl_2_ and 15 MgCl_2_. *L*, box plots showing the average amplitude of compound EJPs evoked at 0.2 Hz. A one-way ANOVA detected no significant differences (*P* = 0.201). *M*, box plots of the average amplitude of mEJPs collected during the same recordings used to collect EJPs (N). No significant differences were found using Kruskal–Wallis one-way ANOVA on ranks (*P* = 0.511). *N*, Box plots of the average QC calculated as in panel H, using the corrected EJP and mEJP data shown in panels L and M (current clamp). A one-way ANOVA detected no significant differences (*P* = 0.185). *O*, depression ratio box plots (ratio of the 30th to 1st EJC). An asterisk indicates significantly greater depression when ArgK1 was knocked down (*P* < 0.014); one-way ANOVA with Holm–Sidak *post hoc* tests. Box plots show mean (dotted line) and median, with 25%–75% boxes and 5%–95% whiskers. OK6-GAL4 was used to express transgenes in panels B–E, whereas nSyb-GAL4 was used in F–O.

As an initial test of the impact of knocking down ArgK1 on NT release, we used single muscle fibre voltage clamp and current clamp techniques to analyse NT release from two MNs that synapse on muscle fibre 6 (Fig. 3A). Electrophysiology was performed on muscle fibre 6 as it is the largest and provides the best outcome when trying to maintain TEVC, especially when stimulating at high frequencies. During fictive locomotion MN6/7-Ib fires at 21.3 Hz, whereas MNSNb/d-Is fires at 7.8 Hz (Justs et al., 2022). We chose a supramaximal stimulus frequency for the two MNs (60 Hz; Fig. 3B), one that might reasonably test the capacity of the MNs to sustain NT release if energy metabolism is impaired, and we maintained stimulation for approximately half the period expected for a contraction cycle during unrestrained locomotion (Fig. 3C). The amplitude of the *compound* excitatory junctional current (EJC) was not significantly different at the start of the stimulus train (Fig. 3D; *P* = 0.537; Table 1), but neurotransmission decreased more rapidly in ArgK1 KD terminals (Fig. 3E; *P*<0.001; Table 1).

We then sought to determine whether the phosphagen system plays a role in sustaining release over longer time periods. Physiological assays testing a reliance on energy metabolism require physiologically relevant loads, and these can be induced only using high levels of extracellular Ca^2+^. However high Ca^2+^ levels evoke massive muscle contraction at high stimulus frequencies, and recordings cannot be maintained beyond fractions of a second. Glutamate, added to the bath to stifle muscle contraction during metabolic imaging, cannot be used, as this extinguishes the electrophysiological signs of NT release. We therefore chose a lower stimulus frequency (10 Hz; Fig. 3F), one that would allow us to maintain a TEVC over several minutes (Fig. 3G). To guard against the possibility that other ATP regeneration mechanisms compensate when ArgK1 is knocked down, we knocked down AKs in parallel. In mammals inhibition of CKs has been shown to increase the phosphotransfer flux through AKs (Dzeja et al., 1996). There are three AK isozymes in mammals (Dzeja & Terzic, 2009), and three have been identified in *Drosophila* (Dak1, Adk2 and Adk3; flybase.org) for which RNAi lines are available. Our analysis of QC, gleaned from current-clamp recordings at 0.2 Hz prior to TEVC recordings, showed that the initial evoked release was similar between conditions when knocking down Argk1 and alternate AKs (Fig. 3H; *P* = 0.336; Table 1). Furthermore we were unable to detect any deficit in neurotransmission over a sustained period of 10 Hz stimulation (Fig. 3I; *P* = 0.725; Table 1).

In a preliminary test of the impact of ArgK1 KD on neurotransmission under conditions in which glycolytic activity cannot compensate, we replaced carbohydrates (glucose) in the saline with ketone bodies (hydroxybutyrate) (Fig. 3J). Ketone bodies such as hydroxybutyrate are available for oxidative phosphorylation through fatty acid metabolism but not glycolysis. A stimulus frequency of 42 Hz was adopted as a supramaximal frequency (double the endogenous firing frequency of MN6/7-Ib; Fig. 3K) as the experimental failure rate at 60 Hz (Fig. 3C) proved to be too high to obtain enough data for each of the four treatments. We found no difference in the amplitude of evoked EJPs, mEJPs or the calculated QC prior to implementation of a TEVC or train stimulation (Fig. 3L, *P* = 0.201; Fig. 3M, *P* = 0.511; Fig. 3N, *P* = 0.185, respectively; Table 1). Furthermore although we still observed more rapid depression in neurotransmission when ArgK1 was knocked down (Fig. 3O; *P* = 0.0137; Table 1), we saw no greater depression in the absence of glucose that limited glycolytic compensation; that is it does not appear that, in the absence of a phosphagen system, recourse to glycolysis rescues terminals from a greater deficit.

### Ca^2+^ pumping during burst firing is not impaired by ArgK1 KD

**Figure 4.**
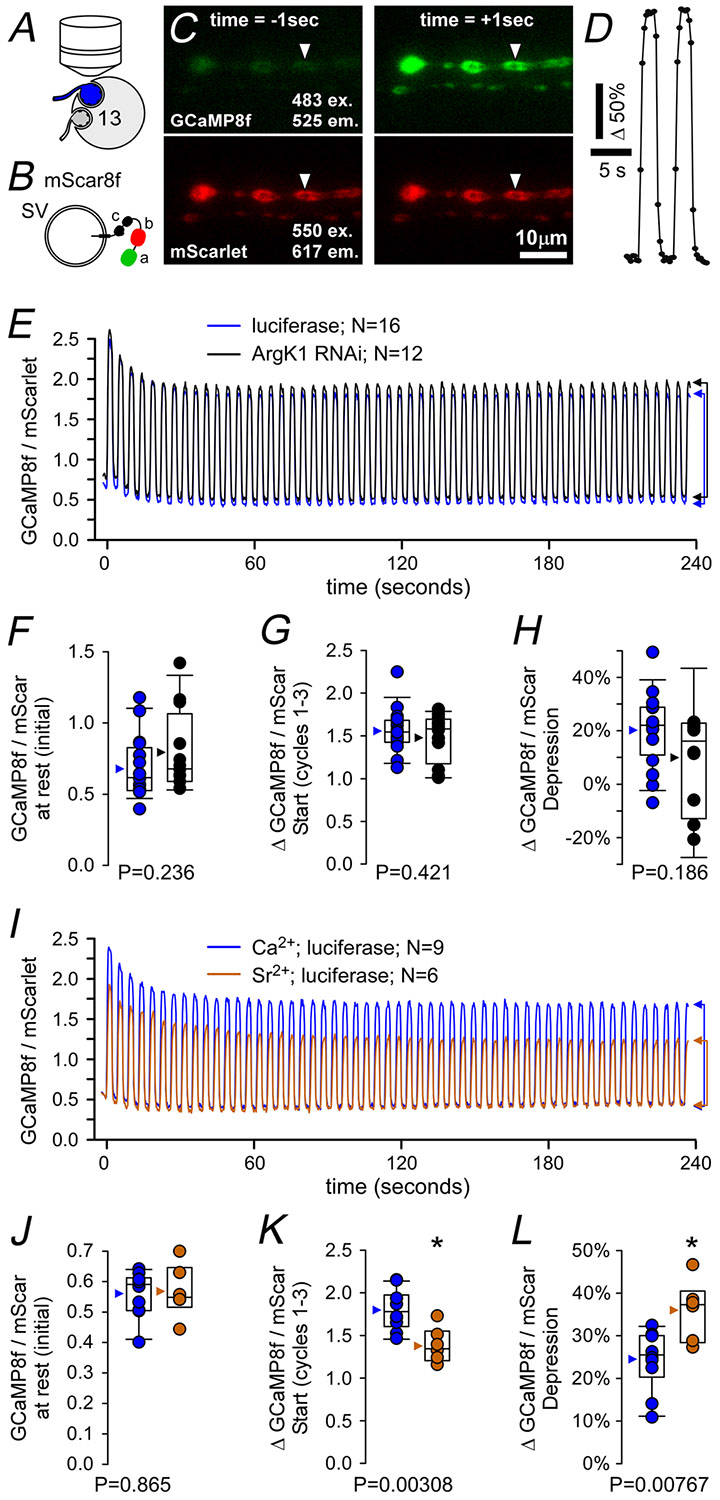
Imaging of presynaptic Ca^2+^ levels and Ca^2+^ pumping capacity after ArgK1 (arginine kinase 1) KD (knock-down) *A*, a diagram of a transverse section through muscle fibre 13, innervated by motor neurons (MNs) MN13-Ib (blue) and MNSNb/d-Is (grey), where fluorescence is recorded from MN13-Ib but not MNSNb/d-Is. *B*, representation of the pseudoratiometric Ca^2+^ indicator mScar8f tethered to a synaptic vesicle (SV). The mScar8f polypeptide is made up of GCaMP8f (a) tethered to the C-terminus of mScarlet and (b) tethered to the C-terminus of the truncated vesicular synaptic protein synaptotagmin (Syt; c). *C*, images of MN terminals on muscle fibre 13 expressing mScar8f. Images were taken before and after 50 Hz nerve stimulation, using the specified excitation and emission wavelengths. Arrowheads provide registration points in image panels. The pattern of mScar8f is reminiscent of cysteine string protein (CSP) staining at the NMJ (neuromuscular junction), which might be expected as mScar8f is targeted to SVs. *D*, an example trace of the GCaMP8f-to-mScarlet fluorescence intensity ratio during two cycles of nerve stimulation, taken from a single trial (*N* = 1) without signal averaging. Image pairs were collected every 250 ms to generate a single ratio measurement (point). An increase in the ratio indicates an increase in the cytosolic Ca^2+^ concentration ([Ca^2+^]_c_). *E*, plots showing the average GCaMP8f-to-mScarlet ratio over 55 cycles from larvae in which ArgK1 has been knocked down (UAS-ArgK1 dsRNA (41697) RNAi line; *N* = 12) and control larvae in which the same MN driver is used to express luciferase (UAS-luciferase; *N* = 16). *F*, plots of the ratio prior to nerve stimulation (at rest) from each preparation. *G*, plots of the average amplitude of the ratio response over the first three cycles of the 55-cycle protocol. *H*, plots of the change (depression) in the amplitude of the ratio between the first and last three cycles of the 55-cycle protocol. Data in panels A–F collected in HL6 with 2 mM CaCl_2_, 15 mM MgCl_2_ and 7 mM l-glutamate. E–H: black lines/symbols used for ArgK1 RNAi, blue for control. *I*, plots of the ratio during cycles of nerve stimulation in the HL6 described earlier (*N* = 9), and with 4 mM SrCl_2_ and 2 mM EGTA instead of 2 mM CaCl_2_ (*N* = 6). Control larvae expressing luciferase used for both Ca^2+^ and Sr^2+^ conditions. *J–L*, plots as in panels F–H. Asterisks denote significant differences as determined by Student’s *t* test. Box plots show mean (dotted line) and median, with 25%–75% boxes and 5%–95% whiskers. nSyb-GAL4 was used to drive the expression of transgenes. Mann–Whitney rank-sum test in panels F and H; unpaired Student’s *t* tests used on all other two-sample comparisons. I–L: brown lines/symbols used for Sr^2+^, blue for Ca^2+^.

Ultimately the metabolic loads we can impose while monitoring neurotransmission are moderate at best, due to the muscle contraction that occurs when nerves are stimulated at high frequency in the presence of physiological levels of extracellular Ca^2+^. Using Ca^2+^ imaging techniques we tested the capacity of ArgK1 KD terminals to pump Ca^2+^ and function under conditions of a metabolic load beyond that we could impose during electrophysiological recordings. In the case of fluorescent metabolic reporters, we used MN13-Ib terminals to obtain a robust signal (Fig. 4A). MNs, expressing the genetically encoded pseudoratiometric Ca^2+^ indicator mScar8f (Fig. 4B and C) (Li et al., 2021), were stimulated at 50 Hz (slightly above their 42 Hz endogenous firing frequency) for 2 s at 4-s intervals for 4 min (Fig. 4C–E). Importantly although it was only Ca^2+^ cycling that was assessed, these stimulus trains initiate APs and trigger NT release imposing a substantial metabolic burden as it requires the recycling and refilling of SVs in addition to resetting Na^+^, K^+^ and Ca^2+^ gradients across the plasma membrane. Our readout of the terminals’ ability to function was assessed through its ability to remove Ca^2+^ in response to each stimulus cycle (Fig. 4C). Our expectation was that, beyond a certain metabolic load in ArgK1 KD terminals, ATP levels would fall below a particular threshold and Ca^2+^ levels would become dysregulated. Despite a robust metabolic load Ca^2+^ cycling appeared to be as efficient in ArgK1 KD terminals as in control terminals (Fig. 4F–H; Table 1).

To confirm that this assay has the power to detect a deficit in energy metabolism, we repeated the assay in the presence of extracellular Sr^2+^ rather than Ca^2+^, as a positive control (Fig. 4I–L). Like Ca^2+^ Sr^2+^ permeates *Drosophila* voltage-gated Ca^2+^ channels (VGCCs) (Chouhan et al., 2012) where it triggers NT release (Jan & Jan, 1976), supporting the different aspects of presynaptic activity that impose a greater metabolic energy demand. Unlike Ca^2+^, however, Sr^2+^ is far less effective at activating the Krebs cycle enzymes of oxidative phosphorylation (McCormack & Osbaldeston, 1990) to keep pace with any increase in presynaptic energy demands. Here in this positive control we observed greater depression during Sr^2+^ cycling than during Ca^2+^ cycling (Fig. 4L; *P* = 0.00767; Table 1), indicating that the absence of a Ca^2+^ cycling deficit in ArgK1 KD terminals (Fig. 4H; *P* = 0.186) was due to the absence of a deficit, rather than the absence of a capacity to detect a deficit.

### ArgK1 KD results in minimal impairment of musculoskeletal performance

Although the cyclical Ca^2+^ pumping protocol demonstrated in Fig. 4 imposed a metabolic load beyond levels we could impose during electrophysiology, the load was still only moderate in the context of what might be expected *in vivo*. If MN13-Ib fires at 42 Hz, with a duty cycle of 0.8 (Klose et al., 2005), then 8160 APs will occur over a period of 4 min. However we imposed only 6060 impulses in the Ca^2+^ cycling protocol (50 Hz × 2 s × 240/4). Longer Ca^2+^ cycling protocols were problematic, leading to fluorophore bleaching, phototoxicity and difficulties in maintaining focus. We were also unable to impose higher FRs or more closely spaced cycles as it diminished our ability to quantify the cycles. Therefore to challenge a MN’s capacity to maintain neurotransmission, not just Ca^2+^ cycling, over longer periods of time, we used opsins to excite MNs and we monitored larval contraction. Significantly this *in vivo* assay tests MN performance although sidestepping the confound of motivation to perform.

**Figure 5.**
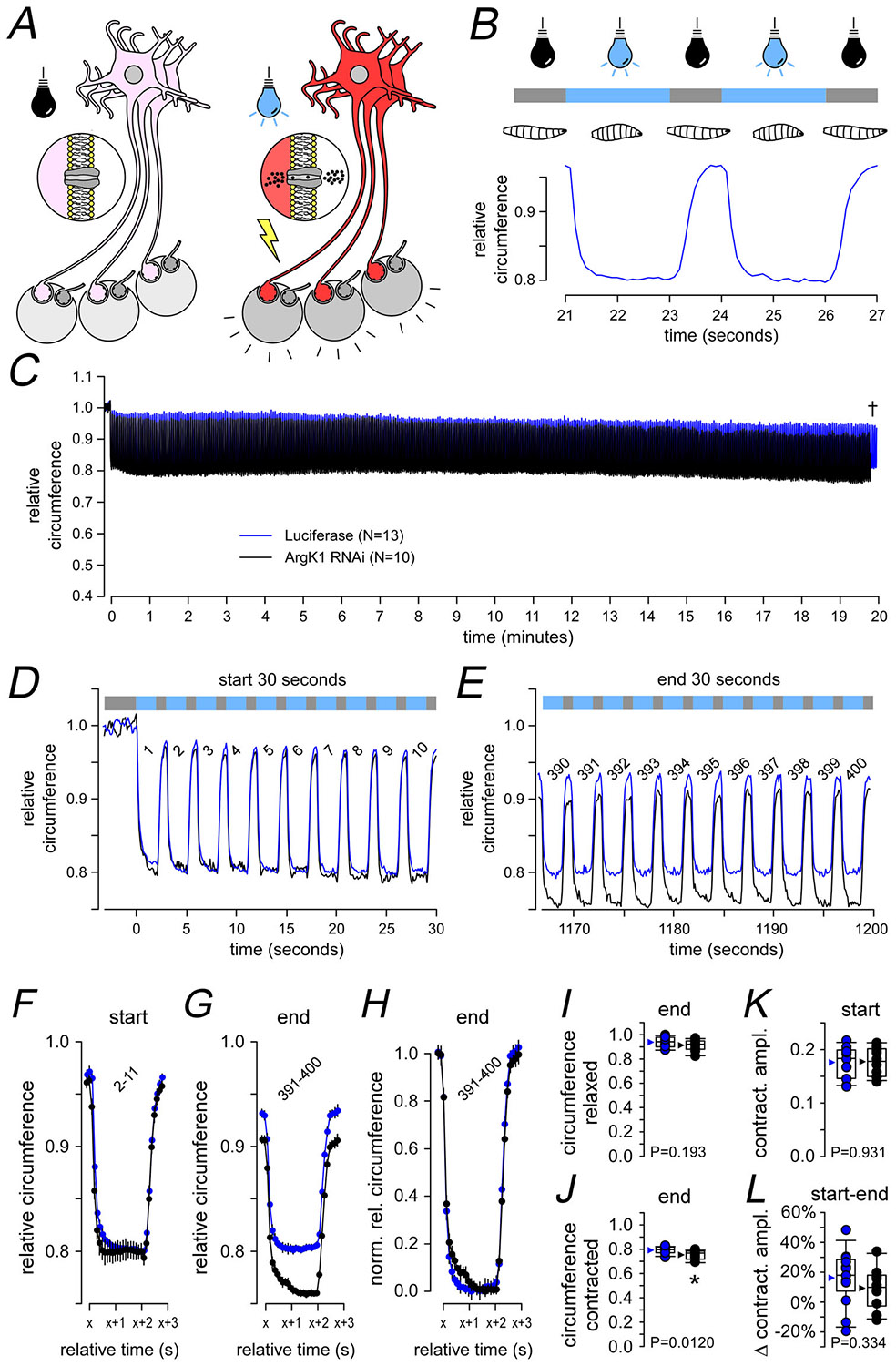
Analysis of musculoskeletal performance after ArgK1 (arginine kinase 1) KD (knock-down) *A*, channelrhodopsin-2 (ChR2) expressed in MNs (motor neurons) can be light activated to cause MN firing, neurotransmitter release and muscle contraction. OK371-GAL4 drives strongly in all MNs with type-Ib terminals (22 in each hemi-segment; Hoang & Chiba, 2001) but only weakly in those MNs with type-Is terminals. *B*, cyclical bouts of blue-light illumination are effective at causing immediate and sustained larval contraction that ceases upon cessation of illumination. *C*, a normalized plot of cyclical larval contraction showing the average perimeter measurement of individual larvae in which ArgK1 KD (knock-down) has been knocked down (UAS-ArgK1 dsRNA (41697) RNAi line; *N* = 10; black trace), and control in which the same MN driver (OK371-GAL4) is used to express luciferase (UAS-luciferase; *N* = 13; blue trace). The dagger indicates that the final 10 s of the ArgK1 KD trace is omitted to allow a comparison between larvae. *D*, a plot of the detail of the first 10 cycles (numbered) of larval contraction taken from panel C. *E*, detail of the last 10 cycles (numbered) of larval contraction taken from panel C. *F*, a plot of the average of contraction cycles 2–11 in panel C. Error bars show standard deviation in panel F*. G*, the average of the last 10 contraction cycles (391–400) in panel C. *H*, the plots in panel G normalized to the average of the first two data points in each trace. *I* and *J*, box plots of the relative circumference of the larvae at maximum rest between cycles (I) and maximum contraction during cycles (J) over the last 10 contraction cycles. *K*, box plots of the change in the normalized perimeter between rest and maximum contraction, for each larva, over contraction cycles 2–11. *L*, box plots of change in the contraction amplitude over the last 10 contraction cycles compared to cycles 2–11. Values are shown from all preparations; mean (arrowhead), median (line), 25–75 percentile box and 10%–90% whiskers. Student’s *t* tests are used to test for significance in plots I–L. OK371-GAL4 (two copies) were used to drive the expression of transgenes. C–L: black lines/symbols used for ArgK1 RNAi, blue for control.

A mutated form of channelrhodopsin-2 (H134R-ChR2; Nagel et al., 2005; Pulver et al., 2009), which is activated by blue light (Fig. 5A), was expressed using a MN-specific driver (OK371-GAL4). Consistent with our focus on MNs with type-Ib terminals, OK371 exhibits much higher expression in type-Ib terminals relative to type-Is terminals. The larvae were illuminated for 2 s at 3-s intervals, causing immediate, robust and consistent cycles of larval contraction (Fig. 5B). Rest periods between cycles of illumination were brief (1 s) to allow recovery but without providing larvae the opportunity to locomote out of the field of view. Individual larvae were subjected to cyclical illumination for 20 min before data were analysed and pooled (Fig. 5C). During the development of this assay, we demonstrated that larvae with a reduced volume of mitochondria in their MN terminals (*dmiro* KD) were unable to sustain contractions, confirming the capacity of this assay to detect contraction deficits resulting from impaired energy metabolism in MNs (Arab et al., 2025). After 20 min ArgK1 KD larvae appeared to be cycling between states that were marginally more contracted than the control larvae (Figs 5E and I, relaxed, *P* = 0.193; Fig. 5J, contracted, *P* = 0.0120), yet we were at a loss to explain how such a phenotype might arise. Ultimately however the extent of ArgK1 KD larval contraction was indistinguishable from control over the first 10 cycles (Fig. 5D, F and K; *P* = 0.931), and ArgK1 KD larval contraction was no more depressed than in control larvae after 20 min (Fig. 5E, G, H and L; *P* = 0.334; Table 1).

### Simulations of [ATP] during endurance assays show little depletion in the absence of a phosphagen system

**Figure 6.**
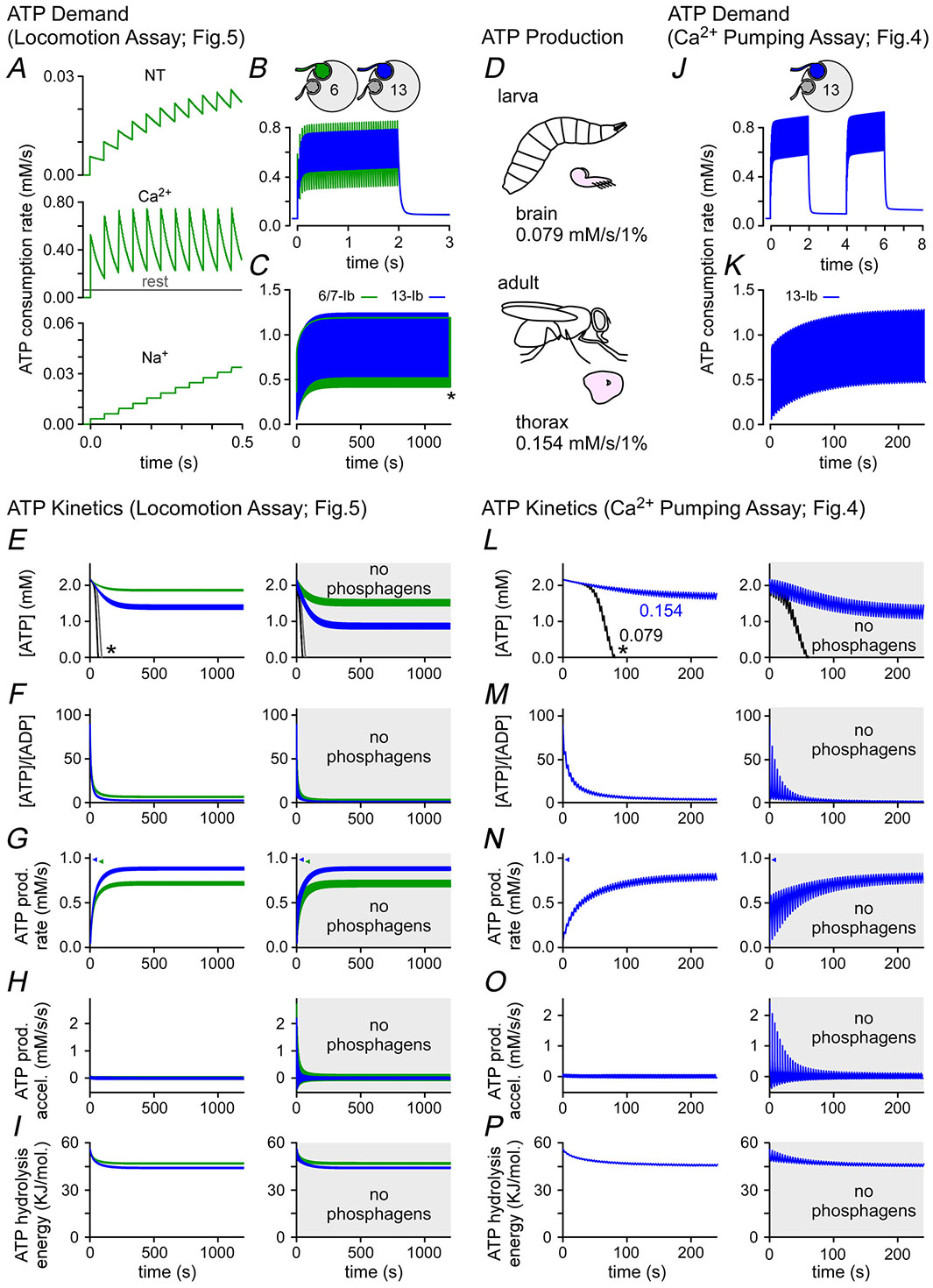
Simulation of ATP levels during prolonged nerve stimulation after ArgK1 (arginine kinase 1) KD (knock-down) Consumption rates (panels A, B, C, J, K) are obtained from eqn (2) using the respective terminals’ parameters and specified firing cycle. Production rates (panels G, H, N, O) are obtained from eqns (4) and (5). Concentrations (panels E, F, I, L, M, P) are obtained using the calculation scheme integrating production, consumption and then equilibrating, described in the ‘Simulation of ATP dynamics’ section. Free energy (I, P) is calculated using Δ*G*_0_ + *RT* ln ([ADP][P_i_]/[ATP]), where Δ*G*_0_ = −30.6 kJ/mol, *R* = 8.31445×10^−3^ kJ/mol/K, T = 300 K. *A*, plots of the ATP consumption rate (green) in response to individual action potentials (APs) in MN6/7-Ib terminals at the onset of a peristaltic cycle. Three signalling components were considered; SV recycling/refilling associated with neurotransmitter (NT) release, Ca^2+^ extrusion and Na^+^ extrusion. The time of elevation of each ‘tooth’ in a saw-tooth profile represents the time of each AP. A constant value was calculated for non-signalling activities, and this value, representing all activities, is plotted as a flat line in grey on the Ca^2+^ scale (as the Ca^2+^ scale has the most appropriate range). Units (mM/s) are obtained by dividing the terminal-specific ATP consumption rate estimates (ATP molecules/s) by the volume of the terminal. *B*, plots of the total ATP consumption rate resulting from the summation of each aspect of signalling, along with the non-signalling rate, for two terminals (green, MN6/7-Ib (green) and MN13-Ib (blue)), representing just two of all (~22) motor neurons (MNs), with type-Ib terminals driven by opsins during the locomotion assay. OK371-GAL4 drives only weakly in MNs with type-Is terminals (grey), so their ATP levels have not been simulated. *C*, total ATP consumption rate over the 20-min locomotion assay (Fig. 5). The asterisk represents the omission of the final 20 s of the MN13-Ib trace to allow a comparison between terminals. A green line registers the top of the MN6/7-Ib trace, otherwise obscured below the MN13-Ib trace. *D*, estimates of ATP production rates derived from respirometry measurements of isolated larval brains (Neville et al., 2018) and adult thoraces (Jorgensen et al., 2021; Menail et al., 2022). Brains were not otherwise stimulated, but thoraces were permeabilized and stimulated to their maximum rate of O_2_ consumption. Units (mM/s/1%) were obtained by dividing brain or thorax ATP production rates (mM/s) by tissue-specific mitochondrial volume density, yielding mM ATP per second per 1% mitochondrial density. *E–I*, plots of the cytosolic ATP concentration (E), [ATP]/[ADP] (F), ATP production rate with adult thorax rate caps (0.154 mM/s/1%) denoted on the ordinate (G), ATP production rate acceleration (H) and the free energy available from ATP hydrolysis (I) corresponding to the activity in panel C, in the presence of a phosphagen system (left panels) and in its absence (right panels). A maximum rate of 0.154 mM/s/1% is used throughout. However asterisks in panels E and L indicate where maximum ATP production rate was reduced from 0.154 mM/s/1% to 0.079 (E: MN13-Ib (black line) MN6/7-Ib (grey line), L: MN13-Ib (black line)). *J*, plots of the total ATP consumption rate for MN13-Ib terminals, cyclically driven at 50 Hz for 2 s at 4-s intervals during the Ca^2+^ pumping assay (Fig. 4). *K*, plots of the total ATP consumption rate over the 4-min period of the Ca^2+^ pumping assay protocol. *L–P*, plots of the cytosolic ATP concentration (L), [ATP]/[ADP] (M), ATP production rate (N), ATP production rate acceleration (O) and the free energy available from ATP hydrolysis (P) corresponding to the activity in K, in the presence of a phosphagen system (left panels) and in its absence (right panels). The following starting concentrations and rates were used in panels E–I and L–P; [ATP] = 2.16 mm, [ADP] = 0.024 mm, [P_i_] = 3.8, ATP production limit = 0.154 mM/s/1% (reduced to 0.079 mM/s/1% where indicated by asterisks), K_Ph_ = 39.6, [Arg] = 3.3, [ArgP] = 7.5; sources in the ‘Methods’ section.

The earlier experiments (Figs 3–5) proceeded under the assumption that a neuronal phosphagen system is essential for sustaining presynaptic activity. However to the extent that we could quantify presynaptic performance while driving MNs at high frequencies, we detected little evidence of deficits. To better understand the bioenergetic capacity of these MNs, we simulated various parameters of energy metabolism in different MN terminals using a previously described computation model (Justs et al., 2023) (Fig. 6). The model simulates the same FRs and patterns as those imposed by the experimental protocols that yielded empirical data in Figs 3–5. The model calculates total energy *demand* during presynaptic activity based on previous empirical estimates of NT release and Ca^2+^ entry and based on theoretical estimates of Na^+^ entry (Justs et al., 2022) (Fig. 6A). It calculates ATP production according to the stimulatory influence of the ES ([[ATP]/([ADP][P_i_])]; Wilson, 2017) while limited by production maxima drawn from empirical estimates of oxidative phosphorylation from larval (0.079 mM/s/1%) and adult tissues (0.154 mM/s/1%) (Fig. 6D; Menail et al., 2022; Neville et al., 2018). The model is therefore able to simulate the levels of adenine nucleotides (ATP, ADP and AMP) and inorganic phosphate (P_i_) net of demand and production, and their rate of change in the presence of a phosphagen system or in its absence (Fig. 6E–I and L–P).

The simulations indicate that ATP levels could be maintained if the MNs had an ATP production limit as high as 0.154 mM/s/1% but that ATP levels would exhaust in approximately a minute if the limit was limited to 0.079 mM/s/1% (see asterisks in Fig. 6E and L). When the phosphagen system was removed from the model (panels on right in Fig. 6E–I and L–P), the *average* levels of ATP, [ATP]/[ADP], ATP production rate, ATP production acceleration rate and ATP hydrolysis free energy were largely unaffected, but the *volatility* of each measure increased greatly. Consistently we detected no deficit in the empirical data from the protracted locomotion assay (Fig. 5) or the Ca^2+^ pumping assay (Fig. 4). Our fluorescent reporters show no indication of an increase in volatility that might betray any increase in volatility in ATP levels.

### Simulations of [ATP] at high firing rates show depletion in the absence of a phosphagen system

**Figure 7.**
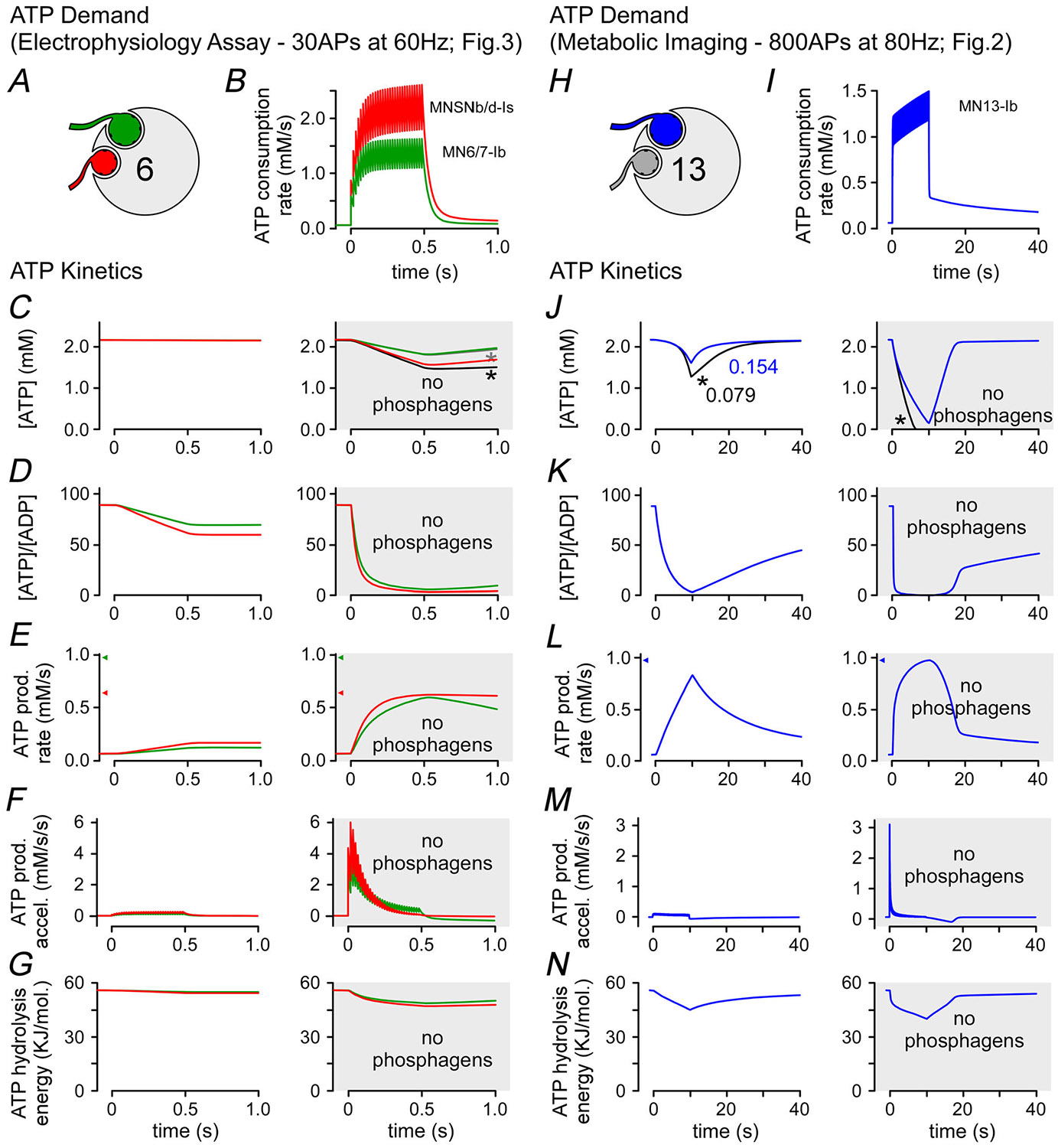
Simulation of ATP levels during high-frequency stimulation after ArgK1 (arginine kinase 1) KD (knock-down) Consumption rates (panels B, I), production rates (panels E, F, L, M), concentrations (panels C, D, G, J, K, N) and free energies (panels G, N) are computed as in Fig. 6. *A*, diagram of the two motor neuron (MN) terminals that release neurotransmitter onto muscle fibre 6 during electrophysiological assays (Fig. 3). *B*, plots of MN terminal ATP consumption rates during a 60 Hz train of 30 action potentials (APs). *C–G*, plots of the cytosolic ATP concentration (C), [ATP]/[ADP] (D), ATP production rate with adult thorax rate caps (0.154 mM/s/1%) denoted on the ordinate (E), ATP production rate acceleration (F) and the free energy available from ATP hydrolysis (G) corresponding to the activity in panel B, in the presence of a phosphagen system (panels on left) and in its absence (panels on right). A maximum rate of 0.154 mM/s/1% is used throughout. However asterisks in panel C indicate where the maximum ATP production rate was reduced from 0.154 mM/s/1% to 0.079 (–Ib: black line; –Is grey line). *H*, diagram of the type-Ib MN terminal (MN13-Ib, blue) on muscle fibre 13 that is imaged during metabolic assays (Fig. 2). *I*, plot of MN terminal ATP consumption rate during an 80 Hz train of 800 APs. *J–N*, plots of the cytosolic ATP concentration (J), [ATP]/[ADP] (K), ATP production rate (L), ATP production rate acceleration (M) and the free energy available from ATP hydrolysis (N) corresponding to the activity in panel I, in the presence of a phosphagen system (panels on left) and in its absence (panels on right). The asterisks (and black line) in panel J indicate where maximum ATP production rate was reduced from 0.154 mM/s/1% to 0.079. The concentrations and rates used in panels C–G and J–N are the same as those listed in Fig. 6, with sources given in the ‘Methods’ section.

The only notable phenotypes in this study occurred when driving MN terminals at FRs well beyond their endogenous rates. During electrophysiological analyses we drove firing at 42 Hz (Fig. 3K–O) and 60 Hz (Fig. 3B–E), representing multiples of the MN endogenous rates on muscle fibre 6: 2.0× and 2.8× of MN6/7-Ib which fires at an average of 21.3 Hz during fictive locomotion, and 5.4× and 7.7× of MNSNb/d-Is which usually fires at 7.8 Hz (Justs et al., 2022). We detected a mildly greater depression of neurotransmission over a period of 0.5 s at both 60 Hz (Fig. 3C and E) and 42 Hz (Fig. 3K and O). Simulation of the 60 Hz stimulus protocols (Fig. 7A–G) revealed little difference in ATP levels when the phosphagen system was removed from the model (Fig. 7C). However a stark difference was observed in the ADP/ATP drawdown (Fig. 7D), and this was reflected in the reduction in the free energy available from ATP hydrolysis, decreasing from 38 to 31 kJ/mol (Fig. 7G). An 80 Hz stimulation protocol was used in the metabolic imaging protocols (Fig. 2), and when the phosphagen system was removed from the model (Fig. 7H–N), the simulation revealed a substantially greater drawdown in ATP levels (Fig. 7J), ADP-to-ATP ratio (Fig. 7K) and the free energy available from ATP hydrolysis (Fig. 7N).

### ArgK1 KD leads to deficits in SV exocytosis and recycling

**Figure 8.**
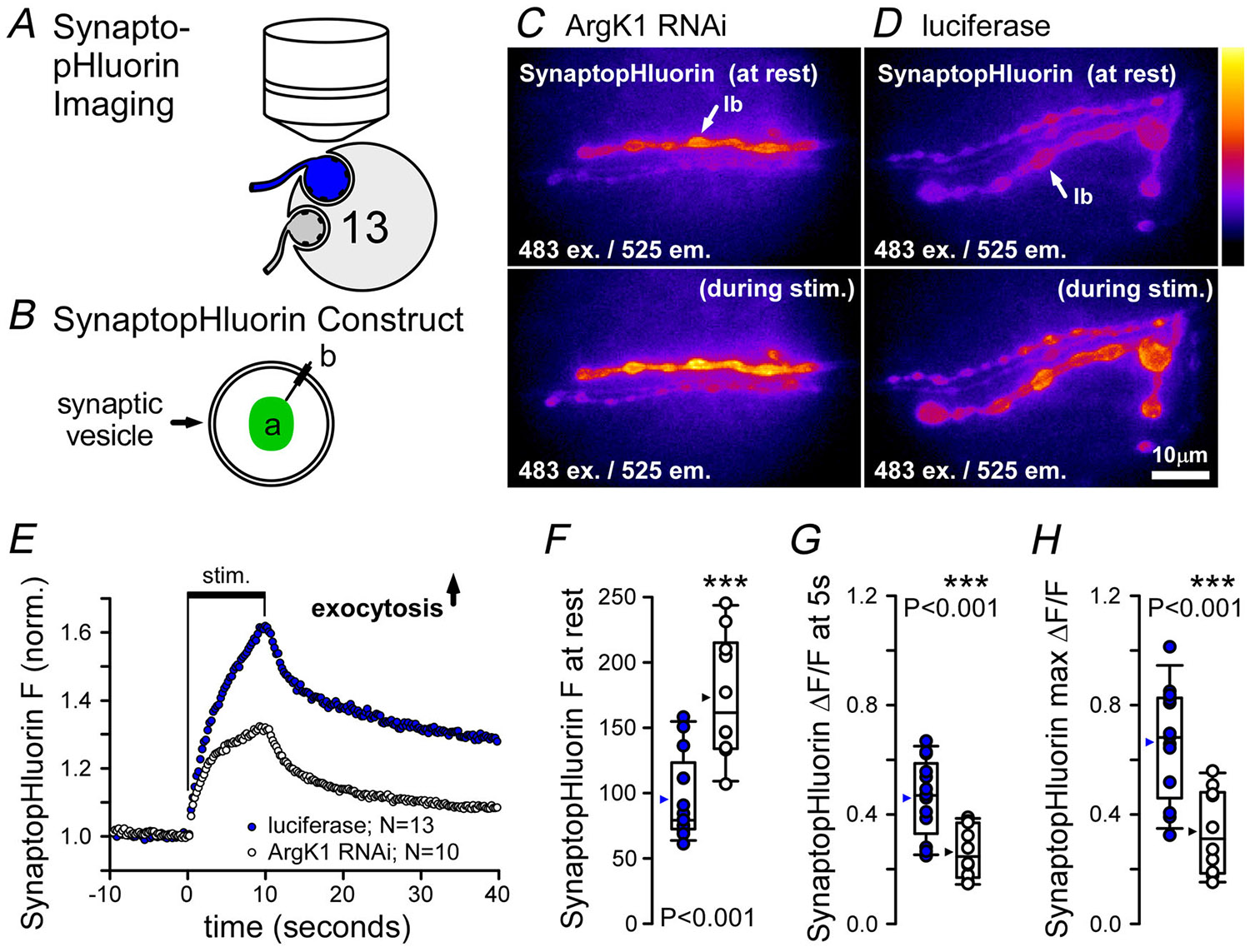
ArgK1 (arginine kinase 1) KD (knock-down) leads to deficits in SV (synaptic vesicle) exocytosis and recycling *A*, a diagram of a transverse section through muscle fibre 13, showing an image focus on one of the two motor neurons (MN13-Ib, blue) that synapse on the fibre. *B*, a diagram of a transverse section through a SV to show the SynaptopHluorin construct used to monitor changes in lumenal pH. The N-terminus of superecliptic pHluorin (a) has been fused to the C-terminus (lumenal domain) of synaptobrevin (Syb, b). *C–D*, images of MN terminals on muscle fibre 13 showing OK6-GAL4-driven expression of SynaptopHluorin, captured prior to stimulation (top panels) and during nerve stimulation (bottom panels) using excitation and emission wavelengths as shown. OK6-GAL4 also drove either the UAS-ArgK1 dsRNA (41697) RNAi line (*N* = 13), to knock down ArgK1 (C), or UAS-luciferase (*N* = 10) as a control (D). *E*, plots of the normalized average SynaptopHluorin fluorescence in MN13-Ib during nerve stimulation at 80 Hz. An increase in the ratio indicates an increase in exocytosis. A measurement was made every 250 ms. *F*, plots of SynaptopHluorin fluorescence at rest. Each point represents a different preparation. *G*, change in SynaptopHluorin fluorescence (ΔF/F_rest_) after 5 s of nerve stimulation. *H*, greatest change in SynaptopHluorin fluorescence (ΔF/F_rest_ after 10 s of nerve stimulation). Images and data in panels C–G were collected from terminals of MN13-Ib in segment 4 in HL6 (2 mM CaCl_2_, 15 mm MgCl_2_, 7 mm l-glutamate). *N* = 13 UAS-ArgK1 dsRNA, and *N* = 10 control, preparations analysed in panels C–H. E–H: black symbols used for ArgK1 RNAi, blue for control. Average traces shown in panel E. Box plots show values from all preparations; mean (arrowhead), median (line), 25–75 percentile box and 10%–90% whiskers. Unpaired Student’s *t* tests.

Although we are unable to use electrophysiology to examine the impact of ArgK1 KD on neurotransmission during 80 Hz stimulation, we can use an optical method if glutamate is added to the bath to stop muscle contraction (Fig. 8A). SynaptopHluorin is a reporter of SV lumenal pH (Fig. 8B) and thus reports SV exocytosis as its fluorescence increases when exposed to an extracellular pH near neutral after being quenched at an acidic pH inside SVs (Ng et al., 2002). As chosen for previous dynamic imaging assays, the large terminal boutons of MN13-Ib were used for this assay. SynaptopHluorin exhibited bright fluorescence associated with clusters of SVs when expressed in MNs (Fig. 8C and D), and it revealed that net SV exocytosis increased at a slower rate during nerve stimulation when ArgK1 is knocked down (Fig. 8E and G; *P*<0.001; Table 1) and peaked at a lower value (Fig. 8H; *P*<0.001; Table 1).

## Discussion

Here we characterized the functional impact of a MN KD of a phosphagen kinase (ArgK1). We observed subtle but measurable phenotypes, with impairments in neurotransmission during high-frequency MN firing that exceeded endogenous rates, although endurance-related challenges remained largely unaffected (Table 1). This is reminiscent of phenotypes in muscle-type cytosolic phosphagen kinase (MM-CK) KO mice where contraction force is reduced during tetanus but endurance is relatively unaffected (Dahlstedt et al., 2000; Steeghs, Benders, et al., 1997; van Deursen et al., 1993). Unfortunately we could find no reports of neurotransmission at the NMJ of CK KO mice that might allow for a comparison with our data or determine whether CK KO mice have deficits in neurotransmission concurrent with deficits in muscle physiology. Whether such deficits in neurotransmission would contribute to deficits in musculoskeletal function is unknown as the NMJ safety factor is high in both *Drosophila* and mammals. Nevertheless the impairments observed here would be expected to have disruptive consequences at central synapses that code information in high firing frequencies. Indeed mouse KO models of brain-specific CK reveal behavioural and spatial learning deficits (Jost et al., 2002; Streijger et al., 2004; Streijger et al., 2005), and ArgK1 KD flies show deficits in short-term memory (Bozzato et al., 2020).

The phenotypes observed here mirror findings in MM-CK mice where nerve-evoked contraction force was reduced during tetanus but endurance was relatively unaffected (Steeghs, Benders, et al., 1997). However as both muscle and nerves were MM-CK deficient, and as the contraction force assays relied on MN function, ambiguity regarding the cellular locus of the deficit remained. This ambiguity was addressed in subsequent experiments, where intact single fibres receiving direct electrical stimulation revealed a deficit in tetanus force during the 70-Hz stimulation (Dahlstedt et al., 2000). These findings are consistent with deficits we observed in neurotransmission (60 Hz, Fig. 3E; 42 Hz, Fig. 3O) and exocytosis (80 Hz, Fig. 8H). The single-fibre studies also used protocols to test endurance but revealed little regarding deficits stemming from CK KO – similar to our observations of Ca^2+^ handling in MNs (Fig. 4H) and sustained body-wall contractions (Fig. 5L) in ArgK1 KD *Drosophila* larvae. Although a detailed examination of mouse MNs and NMJs would be desirable, the literature leaves no doubt that postsynaptic muscle deficits exist in CK KO mice. Due to the significant functional differences between MNs and muscle fibres, the parallels in phenotypes between these cell types are striking.

Tests for concurrent deficits in MN function in CK KO mice were reasonably omitted due to the high ‘safety factor’ at the NMJ, meaning that NT release exceeds the threshold required for muscle contraction (Wood & Slater, 2001). With a safety factor of approximately three- to fivefold in mammals and five- to ninefold in *Drosophila* (Marrus & DiAntonio, 2005), it seems unlikely that anything but a profound deficit in MN performance would result in a deficit in contractile force. Ultimately a muscle contraction force assay (e.g. Ormerod et al., 2022) might resolve the capacity of ArgK1 KD MN deficits to impact larval musculoskeletal performance. Beyond relay synapses like NMJs the role of phosphagen systems in neural circuits with high FRs warrants consideration. Many central neurons in vertebrates – including pyramidal neurons, fast-spiking interneurons and cerebellar mossy fibres – fire at 500 Hz or more (Delvendahl & Hallermann, 2016). Although the cellular locus of the deficit for behavioural and spatial learning deficits has not been elucidated in CK KO mice (Jost et al., 2002; Streijger et al., 2004, 2005), it was concluded that a lack of CK ‘rendered the synaptic circuitry in adult brain less efficient in coping with sensory or cognitive activity related challenges’ (Streijger et al., 2005). ArgK1 likely plays a similar role in *Drosophila*, as ArgK1 KD in mushroom bodies leads to short-term memory impairments in a courtship conditioning paradigm (Bozzato et al., 2020).

The ArgK1 KD phenotypes observed at high stimulus frequencies correspond to deficits in ATP levels (Figs 2A–E and 7C and J), as well as deficits in the ATP-to-ADP ratio (Fig. 7D and K; Justs et al., 2023) and deficits in the free energy available from ATP hydrolysis (Fig. 7G and N). The concurrence of these deficits offers little leverage in elucidating whether the phosphagen system primarily acts as a temporal or spatial buffer of adenine nucleotides. However as we can detect a deficit in the 60 Hz frequency train within four impulses (three intervals at 60 Hz = 50 ms; Fig. 3C), which is prior to a significant deficit in the simulated measures (Fig. 7), we are led to propose that the deficits are most pronounced in microdomains that our simulations cannot capture. That is it is the phosphagen system’s failure to spatially buffer adenine nucleotides that contributes to the ArgK1 KD phenotype. Our simulations represent volume-averaged values; that is in the absence of detail regarding the subcellular distribution of ArgK1, we could not build a spatial component into our simulation. In the cytosolic environment ADP diffusion is greatly impeded, more so than ATP (Jacobus, 1985; Yoshizaki et al., 1990), and, under conditions of intense ATP hydrolysis, ADP builds up faster than can be cleared by diffusion. In its spatial buffering role ArgK1 will clear ADP at sites of ATP hydrolysis, offsetting changes in the ATP-to-ADP ratio and maintaining the free energy of ATP hydrolysis (Mainwood & Rakusan, 1982).

The phenotypes revealed by ArgK1 KD, although mild, can yield some insight into the mechanisms that rely on a presynaptic phosphagen system (Table 1). The greater rate of depression revealed by TEVC (Fig. 3E and O) indicates a reduced capacity to sustain exocytosis at high FRs. Whether both terminal types innervating muscle fibre 6 manifest greater depression, or just one, cannot be discerned from the electrophysiological data. Previous computational modelling indicated the ATP-to-ADP ratio decreased rapidly in type-Ib terminals when the two terminal types fired at their respective endogenous frequencies and when ArgK1 was knocked down (Justs et al., 2023). Here computational modelling that mirrors the electrophysiology protocol shows that the ATP-to-ADP ratio decreases extremely quickly in both terminal types in the absence of a phosphagen system but rapidly in type-Is terminals that are driven at more multiples of their endogenous frequency (Fig. 7D). Our SynaptopHluorin data support our conclusion that exocytosis is the primary deficit and that type-Ib terminals do indeed manifest a deficit as the type-Ib SynaptopHluorin signal is reduced by half when ArgK1 is knocked down (Fig. 8H). Although changes in SynaptopHluorin fluorescence reflect exocytosis net of endocytosis, we consider accelerated endocytosis unlikely and conclude instead that exocytosis itself is impaired. That said the possibility that endocytosis contributes cannot be excluded. The rapid onset of impairment (≤50 ms) would suggest a role for ultrafast endocytosis (Chanaday & Kavalali, 2018; Watanabe et al., 2013). Deficits in endocytosis might be further investigated through the application of a styryl dye such as FM1-43 which can be washed off the plasma membrane if not retrieved from the cell surface by endocytosis (Verstreken et al., 2008). However the use of FM1-43 at the *Drosophila* larval NMJ yields a low signal-to-noise ratio due to the large amount of FM1-43 that loads into the muscle sub-synaptic reticulum that surrounds presynaptic boutons and cannot discriminate deficits on a subsecond time scale.

Phosphagen kinases colocalize with various ATPases (Schlattner et al., 2016), highlighting mechanisms that might be affected by ArgK1 KD. CK associates with both the endoplasmic reticulum and the plasma membrane (Lim et al., 1983; Rossi et al., 1990). Ca^2+^ pumps on both membranes rely on ATP, and there is clear evidence of functional coupling between CK and Ca^2+^ pumps on the endoplasmic reticulum (Korge et al., 1993; Minajeva et al., 1996). However our previous Ca^2+^ imaging data during high FRs (800 at 80 Hz; Justs et al., 2023) show no deficits in Ca^2+^ handling that could explain the exocytosis impairment. Similarly current data (Fig. 4E–H) provide no indication of Ca^2+^ handling deficits. CK has also been isolated with cholinergic and glutamatergic SVs (Friedhoff & Lerner, 1977; Takamori et al., 2006; Wallimann et al., 1985), where it plays a role in SV filling (Xu et al., 1996), but our mEJP amplitude data (Fig. 3M) show no evidence of a filling deficit. Drawing a parallel to CK’s association with myosin in myofibrillar bundles (Krause & Jacobus, 1992), ArgK1 may functionally couple with myosin to transport SVs along presynaptic actin filaments. Actin-myosin-based SV mobilization has been demonstrated at glutamatergic and cholinergic synapses (Mochida, 2020), but whether this process is supported by phosphagen kinases remains unclear. Verstreken et al. (2005) reported that actin-myosin-based mobilization of reserve SVs in *Drosophila* larval MN terminals is vulnerable to low ATP levels, although no acute deficits in exocytosis or endocytosis were detected. Similarly studies on isolated goldfish retinal bipolar cells (Heidelberger, 2001) and cultured mouse hippocampal neurons (Pathak et al., 2015) found that endocytosis was the first process to fail when ATP levels decreased, failing during or before scission. Despite the rapid onset of the deficit (≤50 ms) observed in the current study, we cannot exclude actin-myosin-based SV mobilization as the primary mechanism affected by ArgK1 KD as KD of myosin VI in rat superior cervical ganglion synapses resulted in a deficit within 50 ms (Hayashida et al., 2015).

It is possible that the deficit in exocytosis we have observed is due to acidification of the presynaptic compartment rather than a decrease in ATP levels. We previously reported increased activity-dependent acidification of the MN terminal cytosol after ArgK1 KD (*P*<0.001; Justs et al., 2023), leading to further analysis of presynaptic metabolism with less pH-sensitive reporters used here. Several intracellular presynaptic mechanisms are thought to be sensitive to cytosolic acidification, and foremost among these are VGCCs (Chesler, 2003). It is known that Ca^2+^ current is inhibited by cytosolic acidification in vertebrate neurons (Mironov & Lux, 1991; Tombaugh & Somjen, 1997), although we observed no deficit in Ca^2+^ handling under the same conditions that evoked greater ArgK1 KD-associated acidification (*P* = 0.751; Justs et al., 2023). Recent work by Zefirov et al. (2020) showed that intracellular acidification of MN terminals reduced exocytosis possibly due to inhibition of actin-myosin mobilization of SVs to release sites. Acidification is known to affect the kinetics of actin-myosin interactions (Jarvis et al., 2018; Kohler et al., 2012), and the reduction in tetanus force in CK-deficient muscle may partially result from the greater rate of acidification in CK-deficient muscle (Meyer et al., 1986).

Evidence for glycolytic compensation comes from metabolic imaging. First the pronounced pH decrease after ArgK1 KD (He et al., 2023) is consistent with increased lactic acid production. Second lactate reporters (Laconic, He et al., 2023; LiLac, Fig. 2I) confirm enhanced lactate generation. Third pyruvate accumulation after activity (Fig. 2T) further supports this view, though reduced ADP/ATP exchange at mitochondria could also explain pyruvate buildup. Nevertheless the most parsimonious explanation is that glycolysis is upregulated in compensation, consistent with the general principle that neurons rely on glycolysis to buffer acute energy stress even though it is less efficient than oxidative phosphorylation. Our electrophysiology data provide little corroboration: MN terminals performed similarly when glycolysis was bypassed using ketone bodies or trehalose (Fig. 3J–O). Still due to the brevity (<1 s) of these assays, their ability to discriminate compensatory ATP production is limited. Thus although glycolysis appears to be recruited, its ability to sustain high-frequency neurotransmission remains uncertain, highlighting a potential mismatch between metabolic adaptation and functional demand.

Clarification of the role of phosphagens in MNs could open therapeutic avenues for neurodegenerative diseases involving MN loss and age-related loss of muscle mass and weakness (sarcopenia). Creatine supplementation for these conditions has shown mixed results (Andres et al., 2008; Beal, 2011; Candow et al., 2019; Kreider & Stout, 2021), but there are reasons to be optimistic. First both neurodegenerative diseases and sarcopenia are heterogeneous groups, and we should expect some subgroups to be responsive to treatment, whereas others would be refractory. These subgroups may reveal themselves as the utility of personalized genetic testing evolves. Secondly strategies for phosphagen system repair, maintenance or enhancement should not be limited to creatine supplements but extended to stimulation of the activity and/or expression of CKs and transporters, and the biogenesis of creatine itself. The endogenous regulation of each of these alternative targets is specific to cell type, and a successful strategy for targeting muscle cells might diverge significantly for MNs. For example creatine biosynthesis relies on several biosynthetic enzymes and a membrane transporter, and some but not all elements are found in any given cell type, resulting in brain and muscle tissues with different levels of autonomy with regard to creatine biosynthesis (Ensenauer et al., 2004; Joncquel-Chevalier Curt et al., 2015). Similarly mutations in the creatine transporter gene (*SLC6A8*) cause different pathologies in brain and muscle (Schoch et al., 2006), highlighting that MNs might respond differently to interventions targeting creatine biogenesis or transporter activity.

## Supplementary Material

Supporting Information

Additional supporting information can be found online in the Supporting Information section at the end of the HTML view of the article. Supporting information files available:

## Figures and Tables

**Table 1.** Summary of ArgK1 KD phenotypes.

Presynaptic energy metabolism		
ATP decreases to lower levels during 80 Hz nerve stimulation	Fig. 2E	*P* = 0.0520
ATP/ADP decreases to lower levels during 80 Hz stimulation	Justs ’23*	*P*<0.001
Lactate levels increase faster during 80 Hz stimulation	Fig. 2I and Justs ’23*	*P* = 0.0302 & *P*<0.001
Pyruvate levels increase further after 80 Hz stimulation	Fig. 2O	*P* = 0.173
pH decreases to lower levels during 80 Hz stimulation	Justs ’23*	P<0.001
No differences in pre-stim levels of measures above (as immediately above)	Fig. 2C, H and M Justs ’23*	P = 0.557, 0.144, 0.179 0.237, 0.878, 0.871
**Presynaptic mitochondrial volume density**		
No difference in density	Fig. 2Q	*P* = 0.911
**Presynaptic mitochondrial function**		
No difference in matrix Ca^2+^ levels prior to nerve stimulation	Justs ’23*	*P* = 0.632
No difference in Ca^2+^ uptake during 80 Hz stimulation	Justs ’23*	*P* = 0.223
Matrix pH less alkaline prior to nerve stimulation	Justs ’23*	*P* = 0.002
No difference in pH change during 80 Hz stimulation	Justs ’23*	*P* = 0.124
**Postsynaptic signs of neurotransmission**		
No difference in isolated AP-triggered event amplitude	Fig. 3D, H and N	*P* = 0.537, 0.336, 0.185
No difference in spontaneous uniquantal release amplitude	Fig. 3M	*P* = 0.511
Greater frequency depression at 60 and 42 Hz nerve stimulation	Fig. 3E and O	*P*<0.001, 0.0137
No difference in frequency depression at 10 Hz nerve stimulation	Fig. 3I	*P* = 0.725
**Presynaptic Ca^2+^ handling (net result of influx and extrusion)**		
No difference in pre-stimulus cytosolic Ca^2+^ levels	Fig. 4F and Justs ‘23*	*P* = 0.237 & 0.603
No difference in handling at 50 Hz nerve stimulation (55 2-s cycles)	Fig. 4G and H	*P* = 0.421, 0.186
No difference in handling at 80 Hz nerve stimulation	Justs ’23*	*P* = 0.751
**Body-wall contractions (motor neuron driven)**		
No difference in contraction amplitude (400 2-s cycles)	Fig. 5L	*P* = 0.335
**Neurotransmitter release**		
Synaptic vesicle pH is less acidic	Fig. 8F	*P*<0.001
Net exocytosis is reduced at 80 Hz nerve stimulation	Fig. 8H	*P*<0.001

* Justs ’23 refers to data in Justs et al., 2023, J Physiol. 601, p. 5705.

Abbreviations: AP, action potential; ArgK1, arginine kinase 1; KD, knock-down.

## Data Availability

All data supporting the results of the present study are included within the published paper. Raw data are available from the corresponding author upon reasonable request.

## Key points

- Phosphagen systems are well characterized in muscle, but their role in neurons remains largely unexplored, despite evidence linking phosphagen deficits to behavioural and learning impairments. Knockdown of the primary phosphagen kinase in *Drosophila* larval motor neurons (MNs) caused deficits in exocytosis at the neuromuscular junction within 50 ms of burst firing.
- Endurance activities such as prolonged cycles of presynaptic Ca^2+^ pumping or body-wall contractions were unaffected by phosphagen kinase knockdown.
- Deficits in neurotransmission were consistent with our computational model, which predicts deficits in ATP levels and the ATP-to-ADP ratio only at high firing frequencies.
- The performance deficits we observe in phosphagen kinase-deficient *Drosophila* MNs phenocopy deficits in phosphagen kinase-deficient mouse muscle fibres, where tetanus contraction force is reduced but endurance remains intact.

## Appendix 1: Optimal neuromuscular performance requires MN phosphagen kinases

Justs K, Latner DV, Oliva CD, Arab Y, Bonassi GG, Mahneva O, Crill S, Sempertegui S, Kirchman PA, Fily Y, Macleod GT.

### Confirmation of ArgK1 KD using RNAi

**A**. Confocal images of type-Ib terminals on muscle fibre 6 (MN6/7-Ib). Motor neuron driver OK6-Gal4 expressed myristoylated-tdTomato (red) along with ArgK1 RNAi (41697, bottom panel) or no RNAi (top panel). ArgK1-GFP was endogenously expressed (green). Scale bar: 5 μm.

**B**. Box plots of the fluorescence ratio of GFP to tdTomato to determine RNAi efficacy. Box plots show mean (dotted line), median, 25%–75% boxes, 5%–95% whiskers and individual values (*N* = 5 each); for control, ArgK1 RNAi (35221) and ArgK1 RNAi (41697), left to right. A Kruskal–Wallis one-way ANOVA on ranks shows significant differences between groups (*P* = 0.00919), with Tukey’s pairwise *post hoc* tests indicating that both RNAi KDs were significantly different from control (asterisks). Part of panel B reproduced from Fig. 5*L* of Justs et al. (2023).

Justs, K. A., Sempertegui, S., Riboul, D. V., Oliva, C. D., Durbin, R. J., Crill, S., Stawarski, M., Su, C., Renden, R. B., Fily, Y., & Macleod, G. T. (2023). Mitochondrial phosphagen kinases support the volatile power demands of motor nerve terminals. *J Physiol*, *601*(24), 5705–5732. https://doi.org/10.1113/JP284872

Figures A1-A3
