# Supplementary material for: Optimal neuromuscular performance requires motor neuron phosphagen kinases
Source: J Physiol. Author manuscript; Available in PMC 2026 May 27. (PMC13215054; doi:10.1113/JP288916)
Supplement: Supporting Information [file NIHMS2171522-supplement-Supporting_Information.pdf]

The Journal of Physiology

Optimal Neuromuscular Performance Requires Motor Neuron Phosphagen Kinases

Karlis A Justs, Danielle V Latner, Carlos D Oliva, Yosuf Arab, Gabriel G Bonassi, Olena Mahneva, Sarah Crill, Sergio Sempertegui, Paul A Kirchman, Yaouen Fily, and Gregory T Macleod
DOI: 10.1113/JP288916

Corresponding author(s): Gregory Macleod (gmacleod@tulane.edu)

The following individual(s) involved in review of this submission have agreed to reveal their identity: Peter Gerrard Noakes (Referee #2)

Review Timeline:

Submission Date:	19-Mar-2025
Editorial Decision:	04-Jun-2025
Revision Received:	26-Aug-2025
Accepted:	03-Oct-2025

Senior Editor: Katalin Toth

Reviewing Editor: Matthew Fogarty

Transaction Report:

(Note: With the exception of the correction of typographical or spelling errors that could be a source of ambiguity, letters and reports are not edited. Depending on transfer agreements, referee reports obtained elsewhere may or may not be included in this compilation. Referee reports are anonymous unless the Referee chooses to sign their reports.)

Dear Dr Macleod,

Re: JP-RP-2025-288916 "Optimal Neuromuscular Performance Requires Motor Neuron Phosphagen Kinases" by Karlis A Justs, Danielle V Latner, Carlos D Oliva, Yosuf Arab, Gabriel G Bonassi, Olena Mahneva, Sarah Crill, Sergio Sempertegui, Paul A Kirchman, Yaouen Fily, and Gregory T Macleod

Thank you for submitting your manuscript to The Journal of Physiology. It has been assessed by a Reviewing Editor and by 2 expert referees and we are pleased to tell you that it is acceptable for publication following satisfactory revision.

The referee reports are copied at the end of this email.

Please address all the points raised and incorporate all requested revisions or explain in your Response to Referees why a change has not been made. We hope you will find the comments helpful and that you will be able to return your revised manuscript within 4 weeks. If you require longer than this, please contact journal staff: jp@physoc.org.

Your revised manuscript should be submitted online using the link in your Author Tasks Link Not Available. This link is accessible via your account as Corresponding Author; it is not available to your co-authors. If this presents a problem, please contact journal staff (jp@physoc.org). Image files from the previous version are retained on the system. Please ensure you replace or remove any files that are being revised.

If you do not wish to submit a revised version of your manuscript, you must inform our journal staff (jp@physoc.org) or reply to this email to request withdrawal. Please note that a manuscript must be formally withdrawn from the peer review process at one journal before it may be submitted to another journal.

TRANSPARENT PEER REVIEW POLICY: To improve the transparency of its peer review process The Journal of Physiology publishes online, as supporting information, the peer review history of all articles accepted for publication. Readers will have access to decision letters, including Editors' comments and referee reports, for each version of the manuscript, as well as any author responses to peer review comments. Referees can decide whether or not they wish to be named on the peer review history document.

ABSTRACT FIGURES: Authors are expected to use The Journal's premium BioRender account to create/redraw their Abstract Figures. Information on how to access this account is here: https://physoc.onlinelibrary.wiley.com/journal/14697793/biorender-access.

This will enable Authors to create and download high-resolution figures. If authors have used the free BioRender service, they can use the instructions provided in the link above to download a high-resolution version suitable for publication.
The link provided should only be used for the purposes of this submission. Authors will be charged for figures created on this account if they are not related to this manuscript submission.

LANGUAGE EDITING AND SUPPORT FOR PUBLICATION: If you would like help with English language editing, or other article preparation support, Wiley Editing Services offers expert help, including English Language Editing, as well as translation, manuscript formatting, and figure formatting at www.wileyauthors.com/eeo/preparation. You can also find resources for Preparing Your Article for general guidance about writing and preparing your manuscript at www.wileyauthors.com/eeo/prepresources.

RIGOUR AND REPRODUCIBILITY: We recommend authors consult The Journal's Rigour and Reproducibility requirements to ensure that their manuscript is suitable for publication: https://physoc.onlinelibrary.wiley.com/pb-assets/hub-assets/physoc/documents/TJP-Rigour-and-Reproducibility-Requirements.pdf

REVISION CHECKLIST:
Check that your Methods section conforms to journal policy: https://jp.msubmit.net/cgi-bin/main.plex?form_type=display_requirements#methods

Check that data presented conforms to the statistics policy: https://jp.msubmit.net/cgi-bin/main.plex?form_type=display_requirements#statistics

Upload a full Response to Referees file. To create your 'Response to Referees' copy all the reports, including any comments from the Senior and Reviewing Editors, into a Microsoft Word, or similar, file and respond to each point, using font or background colour to distinguish comments and responses and upload as the required file type.

Please upload two versions of your manuscript text: one with all relevant changes highlighted and one clean version with no changes tracked. The manuscript file should include all tables and figure legends, but each figure/graph should be uploaded as separate, high-resolution files. The journal is now integrated with Wiley's Image Checking service. For further details, see: https://www.wiley.com/en-us/network/publishing/research-publishing/trending-stories/upholding-image-integrity-wileys-image-screening-service

You may also upload:
- 'Potential Cover Art' for consideration as the issue's cover image
- Appropriate Supporting Information (Video, audio or data set: see https://jp.msubmit.net/cgi-bin/main.plex?form_type=display_requirements#supp).

We look forward to receiving your revised submission.

If you have any queries, please reply to this email and we will be pleased to advise.

Yours sincerely,

Katalin Toth
Senior Editor
The Journal of Physiology

REQUIRED ITEMS

- Include a Key Points list in the article itself, before the Abstract.

- Author photo and profile. First or joint first authors are asked to provide a short biography (no more than 100 words for one author or 150 words in total for joint first authors) and a portrait photograph. These should be uploaded and clearly labelled together in a Word document with the revised version of the manuscript. See Information for Authors for further details.

- You must start the Methods section with a paragraph headed Ethical Approval. If experiments were conducted on humans, confirmation that informed consent was obtained, preferably in writing, that the studies conformed to the standards set by the latest revision of the Declaration of Helsinki and that the procedures were approved by a properly constituted ethics committee, which should be named, must be included in the article file. If the research study was registered (clause 35 of the Declaration of Helsinki), the registration database should be indicated, otherwise the lack of registration should be noted as an exception (e.g. The study conformed to the standards set by the Declaration of Helsinki, except for registration in a database). For further information see: https://physoc.onlinelibrary.wiley.com/hub/human-experiments.

- Your manuscript must include a complete Additional Information section, including competing interests; funding; author contributions and acknowledgements.

- Please upload separate high-quality figure files via the submission form.

- You must upload original, uncropped western blot/gel images (including controls) if they are not included in the manuscript. This is to confirm that no inappropriate, unethical or misleading image manipulation has occurred. These should be uploaded as 'Supporting information for review process only'. Please label/highlight the original gels so that we can clearly see which sections/lanes have been used in the manuscript figures. For more information, see: https://physoc.onlinelibrary.wiley.com/hub/journal-policies#imagmanip.

- Please ensure that any tables are editable and in Word format, and wherever possible, embedded in the article file itself.

- Please ensure that the Article File you upload is a Word file.

- A Data Availability Statement is required for all papers reporting original data. This must be in the Additional Information section of the manuscript itself. It must have the paragraph heading 'Data Availability Statement'. All data supporting the

results in the paper must be either: in the paper itself; uploaded as Supporting Information for Online Publication; or archived in an appropriate public repository. The statement needs to describe the availability or the absence of shared data. Authors must include in their statement: a link to the repository they have used, or a statement that it is available as Supporting Information; reference the data in the appropriate sections(s) of their manuscript; and cite the data they have shared in the References section. Whenever possible, the scripts and other artefacts used to generate the analyses presented in the paper should also be publicly archived. If sharing data compromises ethical standards or legal requirements then authors are not expected to share it, but must note this in their statement. For more information, see our Statistics Policy.

- Please include an Abstract Figure file, as well as the Figure Legend text within the main article file. The Abstract Figure is a piece of artwork designed to give readers an immediate understanding of the research and should summarise the main conclusions. If possible, the image should be easily 'readable' from left to right or top to bottom. It should show the physiological relevance of the manuscript so readers can assess the importance and content of its findings. Abstract Figures should not merely recapitulate other figures in the manuscript. Please try to keep the diagram as simple as possible and without superfluous information that may distract from the main conclusion(s). Abstract Figures must be provided by authors no later than the revised manuscript stage and should be uploaded as a separate file during online submission labelled as File Type 'Abstract Figure'. Please also ensure that you include the figure legend in the main article file. All Abstract Figures should be created using BioRender. Authors should use The Journal's premium BioRender account to export high-resolution images. Details on how to use and access the premium account are included as part of this email.

- Please ensure that all figures and tables have a title and legend, and that they have been cited within the main article text.

- Include a Key Points list in the article itself, before the Abstract.

- Author photo and profile. First or joint first authors are asked to provide a short biography (no more than 100 words for one author or 150 words in total for joint first authors) and a portrait photograph. These should be uploaded and clearly labelled together in a Word document with the revised version of the manuscript. See Information for Authors for further details.

- You must start the Methods section with a paragraph headed Ethical Approval. If experiments were conducted on humans, confirmation that informed consent was obtained, preferably in writing, that the studies conformed to the standards set by the latest revision of the Declaration of Helsinki and that the procedures were approved by a properly constituted ethics committee, which should be named, must be included in the article file. If the research study was registered (clause 35 of the Declaration of Helsinki), the registration database should be indicated, otherwise the lack of registration should be noted as an exception (e.g. The study conformed to the standards set by the Declaration of Helsinki, except for registration in a database). For further information see: https://physoc.onlinelibrary.wiley.com/hub/human-experiments.

- Your manuscript must include a complete Additional Information section, including competing interests; funding; author contributions and acknowledgements.

- Please upload separate high-quality figure files via the submission form.

- You must upload original, uncropped western blot/gel images (including controls) if they are not included in the manuscript. This is to confirm that no inappropriate, unethical or misleading image manipulation has occurred. These should be uploaded as 'Supporting information for review process only'. Please label/highlight the original gels so that we can clearly see which sections/lanes have been used in the manuscript figures. For more information, see: https://physoc.onlinelibrary.wiley.com/hub/journal-policies#imagmanip.

- Please ensure that any tables are editable and in Word format, and wherever possible, embedded in the article file itself.

- Please ensure that the Article File you upload is a Word file.

- Papers must comply with the Statistics Policy: https://jp.msubmit.net/cgi-bin/main.plex?form_type=display_requirements#statistics.

In summary:

- If n â‰¤ 30, all data points must be plotted in the figure in a way that reveals their range and distribution. A bar graph with data points overlaid, a box and whisker plot or a violin plot (preferably with data points included) are acceptable formats.

- If n > 30, then the entire raw dataset must be made available either as supporting information, or hosted on a not-for-profit repository, e.g. FigShare, with access details provided in the manuscript.

- 'n' clearly defined (e.g. x cells from y slices in z animals) in the Methods. Authors should be mindful of pseudoreplication.

- All relevant 'n' values must be clearly stated in the main text, figures and tables.

- The most appropriate summary statistic (e.g. mean or median and standard deviation) must be used. Standard Error of the Mean (SEM) alone is not permitted.

- Exact p values must be stated. Authors must not use 'greater than' or 'less than'. Exact p values must be stated to three significant figures even when 'no statistical significance' is claimed.

- A Data Availability Statement is required for all papers reporting original data. This must be in the Additional Information section of the manuscript itself. It must have the paragraph heading 'Data Availability Statement'. All data supporting the results in the paper must be either: in the paper itself; uploaded as Supporting Information for Online Publication; or archived in an appropriate public repository. The statement needs to describe the availability or the absence of shared data. Authors must include in their statement: a link to the repository they have used, or a statement that it is available as Supporting Information; reference the data in the appropriate sections(s) of their manuscript; and cite the data they have shared in the References section. Whenever possible, the scripts and other artefacts used to generate the analyses presented in the paper should also be publicly archived. If sharing data compromises ethical standards or legal requirements then authors are not expected to share it, but must note this in their statement. For more information, see our Statistics Policy.

- Please include an Abstract Figure file, as well as the Figure Legend text within the main article file. The Abstract Figure is a piece of artwork designed to give readers an immediate understanding of the research and should summarise the main conclusions. If possible, the image should be easily 'readable' from left to right or top to bottom. It should show the physiological relevance of the manuscript so readers can assess the importance and content of its findings. Abstract Figures should not merely recapitulate other figures in the manuscript. Please try to keep the diagram as simple as possible and without superfluous information that may distract from the main conclusion(s). Abstract Figures must be provided by authors no later than the revised manuscript stage and should be uploaded as a separate file during online submission labelled as File Type 'Abstract Figure'. Please also ensure that you include the figure legend in the main article file. All Abstract Figures should be created using BioRender. Authors should use The Journal's premium BioRender account to export high-resolution images. Details on how to use and access the premium account are included as part of this email.

- Please ensure that all figures and tables have a title and legend, and that they have been cited within the main article text.

EDITOR COMMENTS

Reviewing Editor:

This is a well-presented paper, with a few core points of concern centering around glycolytic ATP production and clarification of some of the results. Most of these changes are in the presentation of the methods, results and discussion.

If data on different ATP generating substrates are on hand, this would elevate the study. At the very least, some discussion as to the effects of different substrates in the current experimental setup could have affected results is warranted. R2 had some relatively straightforward requests for further specific details.

Discussion about activity/firing rayed of Motor neurons and muscles could be inserted into the discussion.

The equations in particular could be referred to in the figure legend/figure

Approval for experiments and methods of euthanasia need to be reported.

REFEREE COMMENTS

Referee #1:

This manuscript examines the roles of the phosphagen system in the regulation of cellular bioenergetics in Drosophila larval motor neurons. The authors contend that the knockdown of the predominant phosphagen kinase in Drosophila larval MNs, ArgK1, resulted in deficits in presynaptic energy metabolism, which is somewhat mitigated by compensatory glycolysis. Interestingly, the authors did not observe performance deficits in animals with ArgK1 KD in MNs, except for when they stimulated those neurons at frequencies approx. twice that recorded during fictive locomotion. Additionally, computational modeling suggests the possibility that the phosphagen system is more likely to participate in the removal of ADP from the sites of ATP hydrolysis rather than serve as a source of ATP in response to varying bioenergetic burden of those neurons.

Taken together, these studies reveal a nuanced view of the roles played by the phosphagen system in maintaining neuronal function during periods of intense bioenergetic burden. The manuscript is well-written, the experiments are appropriately controlled, and utilizes a host of methodologies, including sophisticated gene editing, live imaging of metabolic sensors, electrophysiology and computation modeling. As such, this manuscript certainly reaches the threshold for publication in its present form.

There are, however, a few minor revisions that would strengthen the overall message of this paper. These revolve around the approach taken to block glycolytic compensation in the ArgK1 KD neurons. The extent to which 20 mM glucose in the HL7A buffer is used by MNs for glycolytic ATP production is not clear as the preferred sugar in Drosophila is trehalose. It is recommended that the authors supplement their recordings using HL7A and HL7C with those that include direct inhibitors of glycolysis added to HL3. For this purpose, the authors could use iodoacetate for blocking GAPDH, rather than 2DG, which enters through glucose transporters could face the same constraints as glucose. Another minor concern relates to the data shown in Fig. 1N. F-Red-tagged variant appears all over the cytosol rather than mitochondria per se. Therefore, apparent colocalization with the GFP-tagged variant under these circumstances is not meaningful.

Referee #2:

This manuscript is a systematic study combined with some novel and elegant assays. However, there is lots of negative data - suggesting that ArgK1 in the assays failed to reveal a role for this phosphagen. Its suggested role was only revealed when the investigators pushed MN stimulation frequencies well beyond the physiological range. This finding may not be surprising given that the Fly's Larva NMJ is an embryonic one - before metamorphosis to adult fly NMJs (i.e., less plastic NMJs). The investigators then embarked on elegant ATP modelling to reveal the physiological role of ArgK1 in the energy dynamics of pre-synaptic nerve terminal function. This was novel idea was to get around the issues of high frequency stimulations, and difficulties in dampening muscle action potential generation, all of which could mask the physiological role of ArgK1 in presynaptic function; a claim that they have substantiated with the negative data shown in figures 3 to 5.

The Extent of ArgK1 knockdown is only detailed in the methods section (lines 534-536), the extent of knockdown is claimed to be 98% with reference to their previous publication. I do think the level of knockdown should be shown in the suppl data and referenced as this mutant is a key to all the data sets.

Numerous points that will enhance reader accessibility are listed below for consideration by the investigators.

POINT 1: Lines 62-63 and 73-80 General comment observation:

Are these buffering capacities different for the types of pre-synaptic inputs that a muscle receives? It seems so - per lines 173-`174.

In the fly some muscle fibres received dual innervation with each MN input having a different level of activity (firing) for example Muscle 6 is innervated by highly an active 1b MN and the less active 1s motor neurons. This issue comes up at various points below (e.g., See points 24,26 and 30).

Line 79-80 does the location and expression levels of these phosphagen kinases within motor nerve terminals differ across highly active Vs low active terminal endings - spatial expression differences?

Novelty and innovation Line 101-111

The investigators have brought to this work novel imaging techniques to monitor Ca2+ pumping during high frequency firing (transmitter release), combined with gross motor performance output and elegant modelling of ATP dynamics - all of which combine to make for an excellent paper, despite some negative results.

POINT 2 RESULTS Figure 1

When 1st mentioning abbreviations even if such abbreviations are assumed to be common knowledge, it is a courtesy to spell them out as it helps make for a manuscript to be understood by a wider audience.

Line 117 - "Green Fluorescent Protein (GFP) DNA" .

Line 121 - oxBFP = ? "ox-Blue Fluorescent Protein (oxBFP)".

POINT 3 Figure 1. Line 119 "1 out of 7" - which one? ArgK1-RA or ArgK1-RE? - this needs to be identified in the text and in Figure 1C' legend and in the panel labels in Fig 1 C (left).

Lines 128-136 A similar comment can be made for the 7 Fusion-Red Peptides shown in Fig 1H, which one(s) are shown in the Fig 1 I to N. Placing the peptide code at the end of ArgK1-GFP- ?? and Fusion-Red '3-?? might help link panels B, and H with their respective stained images.

POINT 4 - registration arrows Figure 1. The image areas that have imaged for the different reporter proteins (GFP, BFP and Fusion-Red) could do well with a registration arrow that allow for tracking of the objects within the panels from left to right. This suggestion could well apply to subsequent figures where one has the same image field.

POINT 5 - Figure 1 legend - explain out what is meant by RH, RB, RC, RA, RE, RD, RG. Either at the beginning of the legend, or perhaps at the end.

POINT 6 Figure 2

Figure 2 A, F and K Lower panels need the same registration arrow as seen in their partner panels (See Point 4 above). Same goes for panel "P".

POINT 7 - line 138 "(PercevalHR)", what dies HR stand for?

POINT 8 Lines 149-150. "trend was found not to be significant" Significant from what? This statement needs clarification. From the legend and the Fig 2A - E, I assume to be Argk1 Knockdown (blue data points compared to white data points). I

also think you need to make this clear in the figure 2's legend that AngK1-RNAi = Knockdown of AngK1 - even though it does appear in the figures. At the end of the figure legend the "t" in student t test should be in ilatics and in lower case, also State that "N = 9-14". See also other figure legends.

POINT 9 - further to point 8. The level of AngK1 know down by RNAi is not given? Unless I have missed this information, perhaps this information can be provided in the supplementary data and cited in this section. See opening comment.

POINT 10; lines 177-178: Make this division of fluorophores clear - namely insert " (division of mito-GFP BY cyto-BFP; Fig. 2P-Q)".

POINT 11 Minor e.g., lines 179-180- P for probability is in italics and should be either kept in upper case "P" or lower case throughout the entire manuscript "p".

POINT 12 Minor - Biological number is usually expressed in italics and in lower case (i.e., 'n"). However, this term can be confused with "n" being used by neuro-electrophysiologists for the denoting the number of release sites. Hence suggest just converting N to "N". This suggestion applies to all figure legends.

POINT 13 Figure 3A - assign the colour code next to the terminal, "innervated by MN6/7-Ib (green) and by MNSNb/d-Is (red)........" and subjected to two electrode voltage clamp (I =?' V =?)." Define what I and V mean.

POINT 14 Ca2+ pumping section Lines 224-252

From the figure4's legend there is now a switch of experiments from muscle 6 to muscle 13, but there is no rationale provided for why the switch - nor does the reader know if this muscle has the same types of motor neuron inputs (fast and slow) as muscle 6. It seems to be dually innervated from Fig. 4 A- one large and one small. What are their respective physiological properties? Are they similar or different to the MNs that innervate Muscle 6? I see from Fig 6 A this may be that muscle 13 is similar to muscle 6, which makes the case for marking the above changes and clarifications.

POINT 15 line 232 and Figure 4. The construct used in Figure 4 needs explanation, either in the text or legend. If is detailed in the methods, then refer to the methods, as the methods come after the results. Perhaps like in figure 1 it might help to orientate the reader to have a schematic of the fused construct and how it works to produce a green and red reporter protein product (described in the legend). One should not assume that one knows how construct was used and works in this experiment by a simple reference to the literature.

POINT 16 line 245 - Spell out "voltage gated Ca2+ channels (VGCCs)'.

POINT 17 lines 246-252 - make clear the rationale of using Sr2+

This for me is unclear. Sr2+ does it or does it not perform the same function as Ca2+ in binding to the Ca2+ sensor Synaptotagmin to regulate release, or in this experiment it just is used as an inhibitor, and thus a way of inducing metabolic stress - I am not clear on.

POINT 18 Figure 4's legend. As noted above "N" in italics the t in student t test lower case and in italics, and cite biological N =? , as the N is not given in the figure.

POINT 19 Figure 5A Schematic should be in the same orientation as Figure 3A.

See my note above - From Fig 5 A it is not clear if muscle 13 singly or dually innervated - it is also frustrating not to have the same transverse view for MN to muscle contacts as shown in Figure 3A, which was informative. In Fig 5A it is not - I am not sure if muscle 13 each receives a single MN input (MN13-Ib) and a partial MN input form a sub-branch of MN13-Ib or a different MN as is the case for muscle 6. I see this has been corrected in figures 6 and 7 so a good idea to be consistent in figure 5.

POINT 20 line 269 "OK317-GAL4 "perhaps needs a reference.

POINT 21 line 269-272- the position and distance of the light source from the larvae during the stimulation period I assume is detailed in methods. If not, then some additional details would be good to have.

POINT 22 ATP modelling Lines 283-284 and Fig 6 legend.

This data set is a centre piece of this manuscript. However, the modelling was only done for the fast-firing MNs - MND6/7-Ib and MN 13-Ib. It would be interesting to look used the modelling for presynaptic energy dynamics for slow firing MNs such as MNSNb/d-1s, as it may inform how they might differ. It could provide some physiological insights into how presynaptic energy is managed across two converging synaptic inputs onto the one muscle. I do see some attempt at comparing slow to fast firing MNs in figure 7, but little is made of this (see comment 26).

In trying to relate this modeling to preceding experimental data, I am not clear how individual action potential (AP) responses fit/relate to the MN stimulation rates used in the experimental settings. The stimulation frequencies are supramaximal, so how does to model work when you increase individual AP frequencies to supramaximal levels. Perhaps I have missed something here.

POINT 23 Figure 6

Figure 6 is complex and hard to understand and is not helped by its figure legend.

A) which are the individual APs? I presume they are the green line deviations as time progresses for NT, Ca^{2+} and Na^{+}. This issue needs to be made clear. The "terminal at rest" is seen the Ca^{2+} - is there a "rest line for the NT and the Na^{+} graphs (not explained out).

B) Muscle 6 and 13 receive two MN inputs - I am assuming green = MN6/7-Ib and Blue is MN13-Ib, both fast firing MN inputs. However, the identity of the grey terminal endings in both muscle needs to mentions in the B's legend. Also what is type of MN input (grey) for muscle 13? Is not mentioned - i.e., is it a fast or slow MN?

B and C) graphs - it looks as if the Blue ATP consumption is superimposed over the top of the green ATP consumption, if so these are merged images - while this is fine, it would be ideal in Suppl Data to see the non-overlapping ATP consumption rates (e.g., a bit like only showing a merged fluorescent image without the separate channels shown)

E-I) as noted above, it would be interesting to model the ATP kinetics of the slow MN - MNSNd/d-Is for muscle 6.

J-K) Similar comments to E-I can also be applied to J-K.

POINT24 Figure 7 A to N.

As noted in my comments about comparing fast and slow MN inputs and ATP kinetics, I see in Fig 7A this has been done for muscle 6 and reveals different responses for fast (green) and slow (red) MN inputs, but little comments is made above these differentness in the text (line 315-316) or on the Fig7's legend. Yes, I note that for most there is no difference - but A and C do show some difference between these two MN inputs, and so worthy of comment.

POINT 25 Figure 7O to S

I note that the analyses were done on the one MN input to Muscle 13 (MN13-Ib), but the second MN input (presumable the grey input in Panel H) is also seen, but there are no analyses. Further there are two genotypes (normal ArgK1 (blue) and ArgK1 RNAi (knockdown) tested under two conditions (rest and stimulated). In panel "O" one needs to see the ArgK1 RNAi images under these two conditions to complement the changes shown in P-S for these two genotypes (i.e., the ArgK1 RNAi at rest and stimulated are not shown).

POINT 26 Figure 7 O-S, needs to include the Biological 'N' numbers for this data set.

POINT 27 Table 1 Line 336 could be also cited at key points in the results sections to help complement observations.

POINT 28 line 343 - Remove hyphens - "Nevertheless".

POINT 29 Discussion A well-considered discussion. Perhaps as noted above, some comment about the roles of phosphagen kinases with respect to fast and slow firing MN terminals might be of interest. In mammalian systems, MN firing patterns help to determine fiber type. How this determination is related to the drosophila neuromuscular system I am not sure. I raise this point as parts of this discussion works in comparing studies in the mouse versus the drosophila.

POINT 30 Role in endocytosis

Lines 428-433 While the evidence points to roles for ArgK1 in exocytosis - it would be good to see if there is an effect on endocytosis in these MNs (ArgK1 Vs ArgK1 RNAi), as suggested for hippocampal neurons. One could test for this idea using FM-143. Perhaps this can be noted as a future.

POINT 31 Method Knockdown of ArgK1 - lines 533-536. Details of the % of knockdown was pleasing to see. Nevertheless, I think the investigators would be well served to include this data, despite being published - shown in Suppl Data (See opening comments).

POINT 32 Methods Section 7 line 657

Perhaps a brief note on study design - namely was the study conducted blind to genotype (ArgK1 Vs ArgK1 RNAI) as per ARRIVE guidelines.

POINT 33 Methods Section 8 ATP equations.

I think it would be helpful either in the main text or on the figure legends to make cross references to these equations used to generate the various graphs shown. I think will help reinforce the high quality and rigour of the data presented.

END OF COMMENTS

This manuscript is a systematic study combined with some novel and elegant assays. However, there is lots of negative data – suggesting that ArgK1 in the assays failed to reveal a role for this phosphagen. Its suggested role was only revealed when the investigators pushed MN stimulation frequencies well beyond the physiological range. This finding may not be surprising given that the Fly's Larva NMJ is an embryonic one – before metamorphosis to adult fly NMJs (i.e., less plastic NMJs). The investigators then embarked on elegant ATP modelling to reveal the physiological role of ArgK1 in the energy dynamics of pre-synaptic nerve terminal function. This was novel idea was to get around the issues of high frequency stimulations, and difficulties in dampening muscle action potential generation, all of which could mask the physiological role of ArgK1 in presynaptic function; a claim that they have substantiated with the negative data shown in figures 3 to 5.

The Extent of ArgK1 knockdown is only detailed in the methods section (lines 534-536), the extent of knockdown is claimed to be 98% with reference to their previous publication. I do think the level of knockdown should be shown in the suppl data and referenced as this mutant is a key to all the data sets.

Numerous points that will enhance reader accessibility are listed below for consideration by the investigators.

POINT 1: Lines 62-63 and 73-80 General comment observation:
Are these buffering capacities different for the types of pre-synaptic inputs that a muscle receives?
It seems so - per lines 173-`174.

In the fly some muscle fibres received dual innervation with each MN input having a different level of activity (firing) for example Muscle 6 is innervated by highly an active 1b MN and the less active 1s motor neurons. This issue comes up at various points below (e.g., See points 24,26 and 30).

Line 79-80 does the location and expression levels of these phosphagen kinases within motor nerve terminals differ across highly active Vs low active terminal endings – spatial expression differences?

Novelty and innovation Line 101-111
The investigators have brought to this work novel imaging techniques to monitor Ca2+ pumping during high frequency firing (transmitter release), combined with gross motor performance output and elegant modelling of ATP dynamics – all of which combine to make for an excellent paper, despite some negative results.

POINT 2 RESULTS Figure 1
When 1st mentioning abbreviations even if such abbreviations are assumed to be common knowledge, it is a courtesy to spell them out as it helps make for a manuscript to be understood by a wider audience.
Line 117 – "Green Fluorescent Protein (GFP) DNA" .
Line 121 – oxBFP = ? "ox-Blue Fluorescent Protein (oxBFP)".

POINT 3 Figure 1. Line 119 "1 out of 7" - which one? ArgK1-RA or ArgK1-RE? - this needs to be identified in the text and in Figure 1C' legend and in the panel labels in Fig 1 C (left).
Lines 128-136 A similar comment can be made for the 7 Fusion-Red Peptides shown in Fig 1H, which one(s) are shown in the Fig 1 I to N. Placing the peptide code at the end of ArgK1-GFP- ?? and Fusion-Red '3-?? might help link panels B, and H with their respective stained images.

POINT 4 – **registration arrows Figure 1**. The image areas that have imaged for the different reporter proteins (GFP, BFP and Fusion-Red) could do well with a registration arrow that allow for tracking of

the objects within the panels from left to right. This suggestion could well apply to subsequent figures where one has the same image field.

POINT 5 – Figure 1 legend – explain out what is meant by RH, RB, RC, RA, RE, RD, RG. Either at the beginning of the legend, or perhaps at the end.

POINT 6 Figure 2
Figure 2 A, F and K Lower panels need the same registration arrow as seen in their partner panels (See Point 4 above). Same goes for panel "P".

POINT 7 – line 138 *"(PercevalHR)", what dies HR stand for?*

POINT 8 Lines 149-150. *"trend was found not to be significant"* Significant from what*?* This statement needs clarification. From the legend and the Fig 2A – E, I assume to be Argk1 Knockdown (blue data points compared to white data points). I also think you need to make this clear in the figure 2's legend that AngK1-RNAi = Knockdown of AngK1 – even though it does appear in the figures. At the end of the figure legend the *"t"* in student t test should be in ilatics and in lower case, also State that *"N = 9-14".* See also other figure legends.

POINT 9 – further to point 8. The level of AngK1 know down by RNAi is not given? Unless I have missed this information, perhaps this information can be provided in the supplementary data and cited in this section. See opening comment.

POINT 10; lines 177-178: Make this division of fluorophores clear – namely insert " *(division of mito-GFP BY cyto-BFP; Fig. 2P-Q*)".

POINT 11 Minor e.g., lines 179-180– *P* for probability is in italics and should be either kept in upper case "*P*" or lower case throughout the <u>entire</u> manuscript "*p*".

POINT 12 Minor – Biological number is usually expressed in italics and in lower case (i.e., ''*n*"). However, this term can be confused with "n" being used by neuro-electrophysiologists for the denoting the number of release sites. Hence suggest just converting N to "*N*". This suggestion applies to all figure legends.

POINT 13 Figure 3A – assign the colour code next to the terminal, "*innervated by MN6/7-Ib (**green**) and by MNSNb/d-Is (**red**)…….." and subjected to two electrode voltage clamp (I =?' V =?)."* Define what I and V mean.

POINT 14 Ca2+ pumping section Lines 224-252
From the figure4's legend there is now a switch of experiments from muscle 6 to muscle 13, but there is no rationale provided for why the switch - nor does the reader know if this muscle has the same types of motor neuron inputs (fast and slow) as muscle 6. It seems to be dually innervated from Fig. 4 A- one large and one small. What are their respective physiological properties? Are they similar or different to the MNs that innervate Muscle 6? I see from Fig 6 A this may be that muscle 13 is similar to muscle 6, which makes the case for marking the above changes and clarifications.

POINT 15 line 232 and Figure 4. The construct used in Figure 4 needs explanation, either in the text or legend. If is detailed in the methods, then refer to the methods, as the methods come after the results. Perhaps like in figure 1 it might help to orientate the reader to have a schematic of the fused construct and how it works to produce a green and red reporter protein product (described in the

legend). One should not assume that one knows how construct was used and works in this experiment by a simple reference to the literature.

POINT 16 line 245 – Spell out "*voltage gated Ca2+ channels (VGCCs)*'.

POINT 17 lines 246-252 – make clear the rationale of using Sr2+
This for me is unclear. Sr2+ does it or does it not perform the same function as Ca2+ in binding to the Ca2+ sensor Synaptotagmin to regulate release, or in this experiment it just is used as an inhibitor, and thus a way of inducing metabolic stress - I am not clear on.

POINT 18 Figure 4's legend. As noted above "*N*" in italics the *t* in student *t* test lower case and in italics, and cite biological *N* =? , as the *N* is not given in the figure.

POINT 19 Figure 5A Schematic should be in the same orientation as Figure 3A.
See my note above - From Fig 5 A it is not clear if muscle 13 singly or dually innervated - it is also frustrating not to have the same transverse view for MN to muscle contacts as shown in Figure 3A, which was informative. In Fig 5A it is not - I am not sure if muscle 13 each receives a single MN input (MN13-Ib) and a partial MN input form a sub-branch of MN13-Ib or a different MN as is the case for muscle 6. I see this has been corrected in figures 6 and 7 so a good idea to be consistent in figure 5.

POINT 20 line 269 "OK317-GAL4 "perhaps needs a reference.

POINT 21 line 269-272- the position and distance of the light source from the larvae during the stimulation period I assume is detailed in methods. If not, then some additional details would be good to have.

POINT 22 ATP modelling Lines 283-284 and Fig 6 legend.
This data set is a centre piece of this manuscript. However, the modelling was only done for the fast-firing MNs - MND6/7-Ib and MN 13-Ib. It would be interesting to look used the modelling for presynaptic energy dynamics for slow firing MNs such as MNSNb/d-1s, as it may inform how they might differ. It could provide some physiological insights into how presynaptic energy is managed across two converging synaptic inputs onto the one muscle. I do see some attempt at comparing slow to fast firing MNs in figure 7, but little is made of this (see comment 26).

In trying to relate this modeling to preceding experimental data, I am not clear how individual action potential (AP) responses fit/relate to the MN stimulation rates used in the experimental settings. The stimulation frequencies are supramaximal, so how does to model work when you increase individual AP frequencies to supramaximal levels. Perhaps I have missed something here.

POINT 23 Figure 6
Figure 6 is complex and hard to understand and is not helped by its figure legend.
A) which are the individual APs? I presume they are the green line deviations as time progresses for NT, Ca2+ and Na+. This issue needs to be made clear. The "terminal at rest" is seen the Ca2+ - is there a "rest line for the NT and the Na+ graphs (not explained out).
B) Muscle 6 and 13 receive two MN inputs – I am assuming green = MN6/7-Ib and Blue is MN13-Ib, both fast firing MN inputs. However, the identity of the grey terminal endings in both muscle needs to mentions in the B's legend. Also what is type of MN input (grey) for muscle 13? Is not mentioned – i.e., is it a fast or slow MN?
B and C) graphs - it looks as if the Blue ATP consumption is superimposed over the top of the green ATP consumption, if so these are merged images - while this is fine, it would be ideal in Suppl Data to

see the non-overlapping ATP consumption rates (e.g., a bit like only showing a merged fluorescent image without the separate channels shown)
E-I) as noted above, it would be interesting to model the ATP kinetics of the slow MN - MNSNd/d-Is for muscle 6.
J-K) Similar comments to E-I can also be applied to J-K.

POINT24 Figure 7 A to N.
As noted in my comments about comparing fast and slow MN inputs and ATP kinetics, I see in Fig 7A this has been done for muscle 6 and reveals different responses for fast (green) and slow (red) MN inputs, but little comments is made above these differentness in the text (line 315-316) or on the Fig7's legend. Yes, I note that for most there is no difference - but A and C do show some difference between these two MN inputs, and so worthy of comment.

POINT 25 Figure 7O to S
I note that the analyses were done on the one MN input to Muscle 13 (MN13-Ib), but the second MN input (presumable the grey input in Panel H) is also seen, but there are no analyses. Further there are two genotypes (normal ArgK1 (blue) and ArgK1 RNAi (knockdown) tested under two conditions (rest and stimulated). In panel "O" one needs to see the ArgK1 RNAi images under these two conditions to complement the changes shown in P-S for these two genotypes (i.e., the ArgK1 RNAi at rest and stimulated are not shown).

POINT 26 Figure 7 O-S, needs to include the Biological '*N*' numbers for this data set.

POINT 27 Table 1 Line 336 could be also cited at key points in the results sections to help complement observations.

POINT 28 line 343 – Remove hyphens – "*Nevertheless*".

POINT 29 Discussion A well-considered discussion. Perhaps as noted above, some comment about the roles of phosphagen kinases with respect to fast and slow firing MN terminals might be of interest. In mammalian systems, MN firing patterns help to determine fiber type. How this determination is related to the drosophila neuromuscular system I am not sure. I raise this point as parts of this discussion works in comparing studies in the mouse versus the drosophila.

POINT 30 Role in endocytosis
Lines 428-433 While the evidence points to roles for ArgK1 in exocytosis - it would be good to see if there is an effect on endocytosis in these MNs (ArgK1 Vs ArgK1 RNAi), as suggested for hippocampal neurons. One could test for this idea using FM-143. Perhaps this can be noted as a future.

POINT 31 Method Knockdown of ArgK1 – lines 533-536. Details of the % of knockdown was pleasing to see. Nevertheless, I think the investigators would be well served to include this data, despite being published - shown in Suppl Data (See opening comments).

POINT 32 Methods Section 7 line 657
Perhaps a brief note on study design - namely was the study conducted blind to genotype (ArgK1 Vs ArgK1 RNAI) as per ARRIVE guidelines.

POINT 33 Methods Section 8 ATP equations.
I think it would be helpful either in the main text or on the figure legends to make cross references to these equations used to generate the various graphs shown. I think will help reinforce the high quality and rigour of the data presented.

Dear Dr Macleod,

Re: JP-RP-2025-288916 "Optimal Neuromuscular Performance Requires Motor Neuron Phosphagen Kinases" by Karlis A Justs, Danielle V Latner, Carlos D Oliva, Yosuf Arab, Gabriel G Bonassi, Olena Mahneva, Sarah Crill, Sergio Sempertegui, Paul A Kirchman, Yaouen Fily, and Gregory T Macleod

Thank you for submitting your manuscript to The Journal of Physiology. It has been assessed by a Reviewing Editor and by 2 expert referees and we are pleased to tell you that it is acceptable for publication following satisfactory revision.

The referee reports are copied at the end of this email.

Katalin Toth
Senior Editor
The Journal of Physiology

REQUIRED ITEMS

- Include a Key Points list in the article itself, before the Abstract.

A Key Points list has now been included.

- Author photo and profile. First or joint first authors are asked to provide a short biography (no more than 100 words for one author or 150 words in total for joint first authors) and a portrait photograph. These should be uploaded and clearly labelled together in a Word document with the revised version of the manuscript. See Information for Authors for further details.

A photo and profile was published for the first author Karlis A. Justs in J Physiol (Lond) as recently as December of 2023. All of the information (pasted below) is still up-to-date.

*“**Karlis A. Justs** completed his PhD in Integrated Biology and Neuroscience at Florida Atlantic University (Florida, USA). He investigated energy metabolism of the nervous system utilizing the Drosophila melanogaster neuromuscular junction (NMJ). He sought to understand how mitochondrial volume and morphology adapt to the energy demands of motor nerve terminals, and the role of the phosphagen system in neurotransmission. He continued his research career as a Post Doctoral Associate at the Florida Scripps Research Institute where he worked on a high-throughput screen to identify small molecules that enhance mitochondrial function to treat neurodegenerative diseases. Currently, he is an Imaging Field Application Scientist at Agilent Technologies.”*

- You must start the Methods section with a paragraph headed Ethical Approval. If experiments were conducted on humans, confirmation that informed consent was obtained, preferably in writing, that the studies conformed to the standards set by the latest revision of the Declaration of Helsinki and that the procedures were approved by a properly constituted ethics committee, which should be named, must be included in the article file. If the research study was registered (clause 35 of the Declaration of Helsinki), the registration database should be indicated, otherwise the lack of registration should be noted as an exception (e.g. The study conformed to the standards set by the Declaration of Helsinki, except for registration in a database). For further information see: https://physoc.onlinelibrary.wiley.com/hub/human-experiments.

Experiments were conducted on arthropods (Drosophila) and not on vertebrates. Neither Institutional Animal Care and Use Committee (IACUC) approval nor ethical approval is required for such experiments. Consistently, looking at recent (2025) articles in J Physiol (Lond) that published *Drosophila experiments*, no paragraph headed “Ethical Approval” was included.

- Your manuscript must include a complete Additional Information section, including competing interests; funding; author contributions and acknowledgements.

An Additional Information section with all components has been added at the end of the manuscript.

- Please upload separate high-quality figure files via the submission form.

Separate high-quality figure files have been uploaded.

- You must upload original, uncropped western blot/gel images (including controls) if they are not included in the manuscript. This is to confirm that no inappropriate, unethical or misleading image manipulation has occurred. These should be uploaded as 'Supporting information for review process only'. Please label/highlight the original gels so that we can clearly see which sections/lanes have been used in the manuscript figures. For more information, see: https://physoc.onlinelibrary.wiley.com/hub/journal-policies#imagmanip.

Western blots and gels were not part of this study.

- Please ensure that any tables are editable and in Word format, and wherever possible, embedded in the article file itself.

Table 1 has now been rendered in Word format.

- Please ensure that the Article File you upload is a Word file.

The uploaded Article file is a Word file.

- A Data Availability Statement is required for all papers reporting original data. This must be in the Additional Information section of the manuscript itself. It must have the paragraph heading 'Data Availability Statement'. All data supporting the results in the paper must be either: in the paper itself; uploaded as Supporting Information for Online Publication; or archived in an appropriate public repository. The statement needs to describe the availability or the absence of shared data. Authors must include in their statement: a link to the repository they have used, or a statement that it is available as Supporting Information; reference the data in the appropriate sections(s) of their manuscript; and cite the data they have shared in the References section. Whenever possible, the scripts and other artefacts used to generate the analyses presented in the paper should also be publicly archived. If sharing data compromises ethical standards or legal requirements then authors are not expected to share it, but must note this in their statement. For more information, see our Statistics Policy.

A Data Availability Statement has been added.

- Please include an Abstract Figure file, as well as the Figure Legend text within the main article file. The Abstract Figure is a piece of artwork designed to give readers an immediate understanding of the research and should summarise the main conclusions. If possible, the image should be easily 'readable' from left to right or top to bottom. It should show the physiological relevance of the manuscript so readers can assess the importance and content of its findings. Abstract Figures should not merely recapitulate other figures in the manuscript. Please try to keep the diagram as simple as possible and without superfluous information that may distract from the main conclusion(s). Abstract Figures must be provided by authors no later than the revised manuscript stage and should be uploaded as a separate file during online submission labelled as File Type 'Abstract Figure'. Please also ensure that you include the figure legend in the main article file. All Abstract Figures should be created using BioRender. Authors should use The Journal's premium BioRender account to export high-resolution images. Details on how to use and access the premium account are included as part of this email.

An Abstract Figure file and legend has now been included.

- Please ensure that all figures and tables have a title and legend, and that they have been cited within the main article text.

All figures and tables have a title and legend, and have been cited within the main article text.

- Papers must comply with the Statistics Policy: https://jp.msubmit.net/cgi-bin/main.plex?form_type=display_requirements#statistics.

In summary:

- If n < 30, all data points must be plotted in the figure in a way that reveals their range and distribution. A bar graph with data points overlaid, a box and whisker plot or a violin plot (preferably with data points included) are acceptable formats.

- If n > 30, then the entire raw dataset must be made available either as supporting information, or hosted on a not-for-profit repository, e.g. FigShare, with access details provided in the manuscript.

- 'n' clearly defined (e.g. x cells from y slices in z animals) in the Methods. Authors should be mindful of pseudoreplication.

- All relevant 'n' values must be clearly stated in the main text, figures and tables.

- The most appropriate summary statistic (e.g. mean or median and standard deviation) must be used. Standard Error of the Mean (SEM) alone is not permitted.

- Exact p values must be stated. Authors must not use 'greater than' or 'less than'. Exact p values must be stated to three significant figures even when 'no statistical significance' is claimed.

The manuscript complies with the Statistics policy.

EDITOR COMMENTS

Reviewing Editor:

This is a well-presented paper, with a few core points of concern centering around glycolytic ATP production and clarification of some of the results. Most of these changes are in the presentation of the methods, results and discussion.

In response to the Reviewing Editor's comments, and the Reviewers comments, we revised figures 1, 2, 4, 5, 6, 7 and 8, added a supplementary figure (S3), and made many revisions to the text.

If data on different ATP generating substrates are on hand, this would elevate the study. At the very least, some discussion as to the effects of different substrates in the current experimental setup could have affected results is warranted. R2 had some relatively straightforward requests for further specific details.

We do not have data on different ATP generating substrates on hand, and, as our laboratory has not retooled to generate electrophysiological data since moving from FAU to Tulane University the prospects of collecting such data are not good. We have now added a discussion section on our evidence for glycolytic compensation and addressed (below) some of specific comments from Reviewer 1 about the use of alternative substrates for ATP generation. However, we felt that our very limited foray into the use of alternative substrates for ATP generation (1/3 of a single figure in an 8 figure manuscript - without detecting an effect) did not give us license to create a discussion section on the topic of the benefits or limitations of alternative substrates.

Discussion about activity/firing rates of Motor neurons and muscles could be inserted into the discussion.

We have added more information about activity/firing rates of motor neurons at the beginning of the functional imaging section of the Results. The issue of functional differentiation between terminal types was not taken up in this manuscript (having been addressed in our 2023 manuscript in J.Physiol. (Lond)), and as this manuscript does not provide <u>any</u> data that can be identified with type-Is terminals of the slower firing MNs we feel that we have little license to open such a discussion. The issue of using two different stimulus frequencies when examining two different type-Ib motor neurons (and their degree of functional differentiation) is now introduced in the Results.

The equations in particular could be referred to in the figure legend/figure.

As suggested, we have now referred to specific equations in the figure legends.

Approval for experiments and methods of euthanasia need to be reported.

All experiments were conducted on arthropods (larvae of fruit flies) and we could find no precedent for approval for such experiments, or method of euthanasia, being reported in J.Physiol. (Lond.).

REFEREE COMMENTS

We thank Reviewer 1 for their time and critical reading of the manuscript.

We have responded in blue text below.

Referee #1:

This manuscript examines the roles of the phosphagen system in the regulation of cellular bioenergetics in Drosophila larval motor neurons. The authors contend that the knockdown of

the predominant phosphagen kinase in Drosophila larval MNs, ArgK1, resulted in deficits in presynaptic energy metabolism, which is somewhat mitigated by compensatory glycolysis. Interestingly, the authors did not observe performance deficits in animals with ArgK1 KD in MNs, except for when they stimulated those neurons at frequencies approx. twice that recorded during fictive locomotion. Additionally, computational modeling suggests the possibility that the phosphagen system is more likely to participate in the removal of ADP from the sites of ATP hydrolysis rather than serve as a source of ATP in response to varying bioenergetic burden of those neurons.

Taken together, these studies reveal a nuanced view of the roles played by the phosphagen system in maintaining neuronal function during periods of intense bioenergetic burden. The manuscript is well-written, the experiments are appropriately controlled, and utilizes a host of methodologies, including sophisticated gene editing, live imaging of metabolic sensors, electrophysiology and computation modeling. As such, this manuscript certainly reaches the threshold for publication in its present form.

We appreciate the reviewer's overall assessment.

There are, however, a few minor revisions that would strengthen the overall message of this paper. These revolve around the approach taken to block glycolytic compensation in the ArgK1 KD neurons. The extent to which 20 mM glucose in the HL7A buffer is used by MNs for glycolytic ATP production is not clear as the preferred sugar in Drosophila is trehalose. It is recommended that the authors supplement their recordings using HL7A and HL7C with those that include direct inhibitors of glycolysis added to HL3. For this purpose, the authors could use iodoacetate for blocking GAPDH, rather than 2DG, which enters through glucose transporters could face the same constraints as glucose.

We appreciate the reviewer's points, both regarding our assumption that glucose could serve as a glycolytic energy substrate in these neurons, and regarding iodoacetate as a superior glycolytic inhibitor given its action independent of transporter properties. While we agree that experiments incorporating iodoacetate (with or without trehalose) would be informative, such studies would require multiple additional control conditions and are beyond our current experimental capacity. More broadly, we acknowledge that our work has only begun to address the extent and mechanisms of glycolytic compensation. We have therefore expanded our discussion of the evidence we provide for glycolytic compensation, while noting that the question of alternative substrates for ATP generation remains to be fully resolved.

Another minor concern relates to the data shown in Fig. 1N. F-Red-tagged variant appears all over the cytosol rather than mitochondria per se. Therefore, apparent colocalization with the GFP-tagged variant under these circumstances is not meaningful.

We thank the reviewer for highlighting this point and agree that our rationale for including Fig. 1N was not made sufficiently clear. The value of this panel is that it allows us to distinguish between simple cytosolic distribution and mitochondrial localization. As mitochondria typically occlude cytosolic fluorophores, cytosolic markers alone produce the appearance of “holes” corresponding to mitochondrial volumes. In contrast, the RFP-tagged ArgK signal is visible throughout the cytosol and within volumes occupied by mitochondria, supporting the conclusion that ArgK is present in both compartments. We have now revised the Results section to make this rationale explicit.

Referee #2:

This manuscript is a systematic study combined with some novel and elegant assays. However, there is lots of negative data - suggesting that ArgK1 in the assays failed to reveal a role for this phosphagen. Its suggested role was only revealed when the investigators pushed MN stimulation frequencies well beyond the physiological range. This finding may not be surprising given that the Fly's Larva NMJ is an embryonic one - before metamorphosis to adult fly NMJs (i.e., less plastic NMJs). The investigators then embarked on elegant ATP modelling to reveal the physiological role of ArgK1 in the energy dynamics of pre-synaptic nerve terminal function. This was novel idea was to get around the issues of high frequency stimulations, and difficulties in dampening muscle action potential generation, all of which could mask the physiological role of ArgK1 in presynaptic function; a claim that they have substantiated with the negative data shown in figures 3 to 5.

The Extent of ArgK1 knockdown is only detailed in the methods section (lines 534-536), the extent of knockdown is claimed to be 98% with reference to their previous publication. I do think the level of knockdown should be shown in the suppl data and referenced as this mutant is a key to all the data sets.

As requested, we have now reproduced the knockdown data in a Supplementary figure (Fig. S3).

Numerous points that will enhance reader accessibility are listed below for consideration by the investigators.

POINT 1: Lines 62-63 and 73-80 General comment observation:

Are these buffering capacities different for the types of pre-synaptic inputs that a muscle receives? It seems so - per lines 173-`174.

Throughout the manuscript we have systematically reported the identity of the inputs being examined in each assay as the two terminal types (Ib and Is) are functionally differentiated, i.e.

type-Ib terminals have a higher firing rate, energy demand and mitochondrial volume density. However, we have no data on the relative ATP buffering capacities of these terminals.

In the fly some muscle fibres received dual innervation with each MN input having a different level of activity (firing) for example Muscle 6 is innervated by highly an active 1b MN and the less active 1s motor neurons. This issue comes up at various points below (e.g., See points 24,26 and 30).

Indeed, these terminals are functionally differentiated, and we consider this information when designing our experiments and attempt to always focus on the more active MNs with type-Ib terminals. We now emphasize this point.

Line 79-80 does the location and expression levels of these phosphagen kinases within motor nerve terminals differ across highly active Vs low active terminal endings - spatial expression differences?

The reviewer asks a question that preoccupies our laboratory and we are exploring techniques for identifying the ATP/ArgP "sinks" served by the phosphagen system, presumably corresponding to an accumulation of ArgK colocalizing with various SVs, ATPases, etc. Whether this differs between terminal types remains to be determined.

Novelty and innovation Line 101-111

The investigators have brought to this work novel imaging techniques to monitor Ca2+ pumping during high frequency firing (transmitter release), combined with gross motor performance output and elegant modelling of ATP dynamics - all of which combine to make for an excellent paper, despite some negative results.

We appreciate this endorsement. One graduate student in particular, Karlis Justs, performed a lot of clever and meticulous experiments only to be met with mostly negative results.

POINT 2 RESULTS Figure 1

When 1st mentioning abbreviations even if such abbreviations are assumed to be common knowledge, it is a courtesy to spell them out as it helps make for a manuscript to be understood by a wider audience.

Line 117 - "Green Fluorescent Protein (GFP) DNA".

We have now addressed this oversight in the rearranged manuscript, i.e. Methods now precede the Results.

Line 121 - oxBFP = ? "ox-Blue Fluorescent Protein (oxBFP)".

We have now addressed this oversight in the rearranged manuscript.

POINT 3 Figure 1. Line 119 "1 out of 7" - which one? ArgK1-RA or ArgK1-RE? - this needs to be identified in the text and in Figure 1C' legend and in the panel labels in Fig 1 C (left).

We have now clarified which isoforms we are referring to in the text of the Results and in the legend of Figure 1.

Lines 128-136 A similar comment can be made for the 7 Fusion-Red Peptides shown in Fig 1H, which one(s) are shown in the Fig 1 I to N. Placing the peptide code at the end of ArgK1-GFP- ?? and Fusion-Red '3-?? might help link panels B, and H with their respective stained images.

We agree that figure 1 can be hard to follow with regard to which isoforms are tagged with FusionRed, and where. They are correct as labelled, but we have revised the panel labels, as suggested, and added the following summary text to the legend: "I-M. *In panels I, L and N, FusionRed was inserted 5' of the catalytic subunit in all isoforms (corresponding to the 1st column in panel H), while in panels J and M, FusionRed was inserted in the 3' position (corresponding to 2nd column)*".

A minor point, but important for appreciating the value of these data - all images represent fluorophores in live cells and tissues, and the only stained images in the manuscript appear in Figure 1 E and F where a fluorophore-conjugated antibody (HRP-CY3) was added to the live preparation, rinsed off, and the tissue was viewed while still alive.

POINT 4 - registration arrows Figure 1. The image areas that have imaged for the different reporter proteins (GFP, BFP and Fusion-Red) could do well with a registration arrow that allow for tracking of the objects within the panels from left to right. This suggestion could well apply to subsequent figures where one has the same image field.

As suggested, we have now added registration arrows in various panels (Fig. 1C, E, F, L, M N; Fig. 2A, F, K, P and Fig. 4C)

POINT 5 - Figure 1 legend - explain out what is meant by RH, RB, RC, RA, RE, RD, RG. Either at the beginning of the legend, or perhaps at the end.

As suggested, we now spell out what the listed isoforms represent, at the start of the figure 1 legend: "*Seven unique mRNA transcripts have been identified and annotated, E.g. Argk1-RH, refers to splice isoform RH.*"

POINT 6 Figure 2

Figure 2 A, F and K Lower panels need the same registration arrow as seen in their partner panels (See Point 4 above). Same goes for panel "P".

As suggested, we have now added registration arrows in image panels of Fig. 2A, F, K and P.

POINT 7 - line 138 "(PercevalHR)", what dies HR stand for?

The "HR" of PercevalHR stands for High Range, and this has now been defined in the text.

POINT 8 Lines 149-150. "trend was found not to be significant" Significant from what? This statement needs clarification. From the legend and the Fig 2A - E, I assume to be Argk1 Knockdown (blue data points compared to white data points). I also think you need to make this clear in the figure 2's legend that AngK1-RNAi = Knockdown of AngK1 - even though it does appear in the figures. At the end of the figure legend the "t" in student t test should be in ilatics and in lower case, also State that "N = 9-14". See also other figure legends.

Covering the comments above:

- Our estimate of the evoked change in the ATeam ratio was greater in ArgK1 knockdown larvae compared to controls. However, this difference did not reach statistical significance at an alpha of 0.05 (P = 0.052). We have therefore removed the term "trend" and clarified this point in the Results text.
- To improve consistency, we now use "ArgK1 RNAi" in the figures and explicitly note that this term is synonymous with ArgK1 KD and ArgK1 dsRNA.
- In Figure 2, we have clarified that ArgK1-RNAi = knockdown of ArgK1, italicized the "t" in Student's *t*-test, and added "N = 9–14" to the legend.

POINT 9 - further to point 8. The level of AngK1 knock down by RNAi is not given? Unless I have missed this information, perhaps this information can be provided in the supplementary data and cited in this section. See opening comment.

The level of knockdown was reported in the Methods section (~98%), and we have now reproduced the knockdown data in a Supplementary figure (Fig. S3), as requested.

POINT 10; lines 177-178: Make this division of fluorophores clear - namely insert " (division of mito-GFP BY cyto-BFP; Fig. 2P-Q)".

We have now made the requested changes.

POINT 11 Minor e.g., lines 179-180- P for probability is in italics and should be either kept in upper case "P" or lower case throughout the entire manuscript "p".

“P” for probability is now consistently presented as upper-case “P” without italics.

POINT 12 Minor - Biological number is usually expressed in italics and in lower case (i.e., 'n"). However, this term can be confused with "n" being used by neuro-electrophysiologists for the denoting the number of release sites. Hence suggest just converting N to "N". This suggestion applies to all figure legends.

The number of preparations is now consistently presented as upper-case “N” without italics, and lower case “n” is not used.

POINT 13 Figure 3A - assign the colour code next to the terminal, "innervated by MN6/7-Ib (green) and by MNSNb/d-Is (red)........" and subjected to two electrode voltage clamp (I =?' V =?)." Define what I and V mean.

The text has been amended as suggested, and “V” is explained as the voltage recording electrode and “I” as the current-passing electrode.

POINT 14 Ca2+ pumping section Lines 224-252

From the figure4's legend there is now a switch of experiments from muscle 6 to muscle 13, but there is no rationale provided for why the switch - nor does the reader know if this muscle has the same types of motor neuron inputs (fast and slow) as muscle 6. It seems to be dually innervated from Fig. 4 A- one large and one small. What are their respective physiological properties? Are they similar or different to the MNs that innervate Muscle 6? I see from Fig 6 A this may be that muscle 13 is similar to muscle 6, which makes the case for marking the above changes and clarifications.

The reviewer is correct that our experiments involve different motor neurons between muscle fiber #6 (Fig. 3) and muscle fiber #13 (Figs. 2, 4 & 8). Both fibers are innervated by type-Ib and type-Is motor neurons.

For electrophysiology, we analyzed the combined output on muscle fiber #6 (Fig. 3), as it is the most practical for voltage-clamp recordings. Neither the evoked nor spontaneous release from type-Is terminals can be discriminated in the combined output.

For imaging studies, we focused on a single type-Ib terminal on muscle fiber #13 (Figs. 2, 4 & 8), which provides the most robust and tractable imaging signal.

Our locomotion assay (Fig. 5) drives all type-Ib motor neurons (OK317-GAL4) but not type-Is neurons.

Importantly, both our electrophysiological and imaging experiments were performed at multiples of the endogenous firing rates that we previously established to be motor-neuron–specific (Chouhan et al., 2010; 2012).

Finally, our computational modeling (Figs. 6 & 7) incorporates these distinct firing rates and simulates each motor neuron type individually.

We have now added text clarifying the rationale for switching between preparations, emphasizing that our focus is on the faster-firing type-Ib motor neurons.

POINT 15 line 232 and Figure 4. The construct used in Figure 4 needs explanation, either in the text or legend. If is detailed in the methods, then refer to the methods, as the methods come after the results. Perhaps like in figure 1 it might help to orientate the reader to have a schematic of the fused construct and how it works to produce a green and red reporter protein product (described in the legend). One should not assume that one knows how construct was used and works in this experiment by a simple reference to the literature.

The mScar8f construct has been further described in the Methods section, and as the Methods have been moved before the Results section the reader is now better informed to interpret the mScar8f experiments.

As suggested, we have also placed a schematic of the fused construct at the beginning of figure 4.

POINT 16 line 245 - Spell out "voltage gated Ca2+ channels (VGCCs)'.

We have now spelled out "voltage gated Ca2+ channels (VGCCs)" as requested.

POINT 17 lines 246-252 - make clear the rationale of using Sr2+

This for me is unclear. Sr2+ does it or does it not perform the same function as Ca2+ in binding to the Ca2+ sensor Synaptotagmin to regulate release, or in this experiment it just is used as an inhibitor, and thus a way of inducing metabolic stress - I am not clear on.

Yes, Sr2+ does perform the same function as Ca2+ in binding to the Ca2+ sensor Synaptotagmin to trigger release, but it is 10-fold less effective at stimulating Ox Phos via activation of Ca2+ sensitive dehydrogenases within the Krebs cycle (McCormack & Osbaldeston, 1990; Denton, 2009). We have clarified the rationale for using Sr2+ through reference to the need for a positive control. Sr2+ induces metabolic stress as energy supply will fall behind demand whenever presynaptic activity increases in the presence of Sr2+.

POINT 18 Figure 4's legend. As noted above "N" in italics the t in student t test lower case and in italics, and cite biological N =? , as the N is not given in the figure.

The number of preparations is now consistently presented as upper-case “N” without italics, and lower case “n” is not used. Student’s *t*-test now consistently shows a lower case “*t*” in italics.

Values for N in the summary panels are the same as in the temporal plots that precede them and this is now made explicit in the figure legends.

POINT 19 Figure 5A Schematic should be in the same orientation as Figure 3A.

See my note above - From Fig 5 A it is not clear if muscle 13 singly or dually innervated - it is also frustrating not to have the same transverse view for MN to muscle contacts as shown in Figure 3A, which was informative. In Fig 5A it is not - I am not sure if muscle 13 each receives a single MN input (MN13-Ib) and a partial MN input form a sub-branch of MN13-Ib or a different MN as is the case for muscle 6. I see this has been corrected in figures 6 and 7 so a good idea to be consistent in figure 5.

Muscle #13 is dually innervated, but muscle #13 and its innervation is not entirely relevant to the experiment in figure 5. We understand that a schematic as suggested by the reviewer might have avoided this misunderstanding and we have now redrawn the schematic in figure 5A. OK371-GAL4 expression allows us to test fatigue in all type-Ib MN terminals on body wall muscles and muscle fibers #6 and #13 are just 2 of 31 muscle fibers in each hemisegment. OK371-GAL4 expresses very weakly in type-Is terminals, and so this assay corresponds to our assays of type-Ib terminals in figures 2, 4 and 8.

As suggested, we have now placed a schematic (transverse) of at least part of the pattern of innervation covered by the OK371-GAL4 driver.

POINT 20 line 269 "OK317-GAL4 "perhaps needs a reference.

We have now referenced the OK371-GAL4 driver (Mahr and Aberle, 2006).

POINT 21 line 269-272- the position and distance of the light source from the larvae during the stimulation period I assume is detailed in methods. If not, then some additional details would be good to have.

The position of the opsin-exciting light source (blue) is only 3 cm from the larva, directly beneath it. The position of the ambient/illuminating light source (red), on the inside surface of a cylinder surrounding the larvae, is approximately 10 cm distant. These details, and many others, are essential to setting up the “behavior” rig, but, just like the details for setting up an electrophysiology rig, these are too numerous to include in this manuscript. We have included details germane to understanding the logic of the experiment, but less germane detail can be found in a methods manuscript (Arab et al., 2025; bioRxiv; under review at J Neurosci Methods)

devoted to the methodology of this assay and the proof of principle in the form of a positive control. Figure 2 of that manuscript is reproduced below to show the overall arrangement of the “behavior” rig.

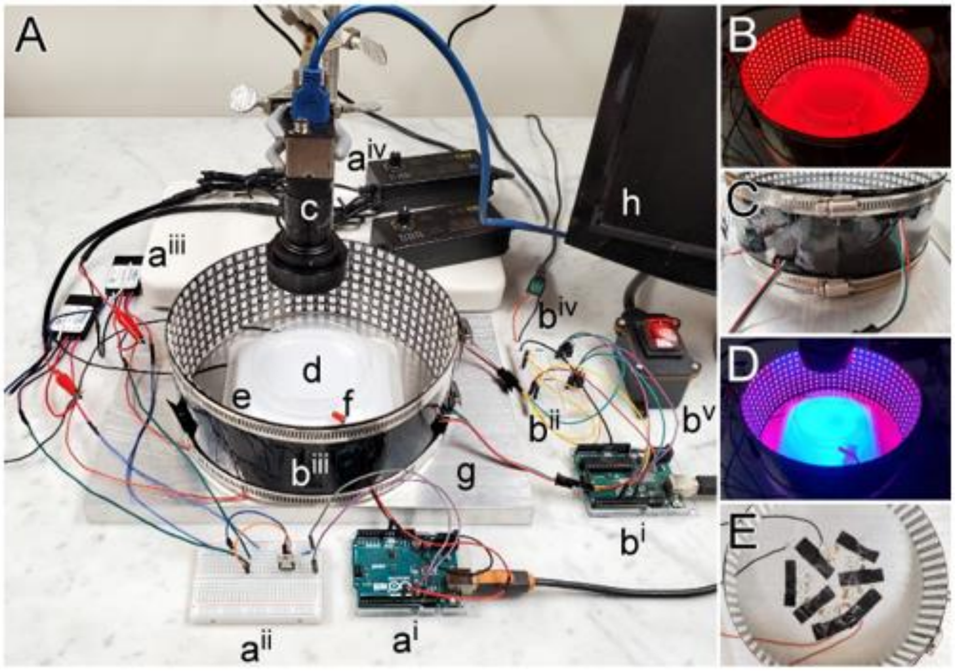

Figure 2. Motor neuron performance assay apparatus.
A, Apparatus photograph with wires teased apart to enable circuit connectivity verification; a - blue LED circuit with an Arduino board (a^{i}) and separate breadboard (a^{ii}), two LED drivers (a^{iii}), two 10V power supplies (a^{iv}); b - red LED circuit with an Arduino board (b^{i}) and separate breadboard (b^{ii}), two LED matrices joined in a cylinder (b^{iii}), beadboard connection to a 5V power supply (out of view) (b^{iv}), inline cord switch for on/off power control of LED matrices (b^{v}); c – camera with a blue light filter in front of the lens (out of view); d - agar-filled Petri dish; e - inverted weigh-boat obscuring the blue LEDs beneath; f - single red LED; g – 19 mm thick aluminum plate heat-sink; h - computer used to control the Arduinos through our open-source Bonsai program.
B, LED matrices programmed to emit red light.
C, Detail of the side of the RGB matrices joined in a cylinder by worm-gear duct-clamps.
D, Blue light emitting from LEDs beneath an inverted weigh-boat used to excite opsins within the larvae, programmed to switch on intermittently, while the surrounding LED matrices emit red line continuously so that larva can be observed without interruption.
E, Blue LEDs (two strings of 3 each) fixed to the aluminum plate in a hexagonal pattern with thermal paste and tape. Weigh-boat and insulation removed to reveal detail.

POINT 22 ATP modelling Lines 283-284 and Fig 6 legend.

This data set is a centre piece of this manuscript. However, the modelling was only done for the fast-firing MNs - MND6/7-Ib and MN 13-Ib. It would be interesting to look used the modelling for presynaptic energy dynamics for slow firing MNs such as MNSNb/d-1s, as it may inform how they might differ. It could provide some physiological insights into how presynaptic energy is managed across two converging synaptic inputs onto the one muscle. I do see some attempt at comparing slow to fast firing MNs in figure 7, but little is made of this (see comment 26).

We did in fact model ATP levels for slow firing MNs with type-Is terminals such as MNSNb/d-Is, and these data are shown in figure 7A-G; as subsequently recognized by the reviewer in point #24. However, we only modelled energy dynamics to complement our experimental protocols and empirical data was only collected from type-Is terminals during electrophysiological protocols (Fig. 3). We did compare slow-firing MNs with fast-firing MNs in our previous article (Justs et al., 2023, J. Physiol), but our empirical data here provide no further license to compare energy dynamics between terminals. It should be noted that our electrophysiological data provide limited insight into the differences in fatigue between terminal types as we made TEVC recordings of compound EJCs, i.e. release from both terminal types, and even the uni-quantal spontaneous EJCs arising from different terminals types could not be discriminated one from the other.

In trying to relate this modeling to preceding experimental data, I am not clear how individual action potential (AP) responses fit/relate to the MN stimulation rates used in the experimental settings. The stimulation frequencies are supramaximal, so how does to model work when you increase individual AP frequencies to supramaximal levels. Perhaps I have missed something here.

Each modeling exercise was performed using the same firing rates imposed in the experimental protocols that generated the empirical data. These rates are considered supramaximal in that they exceed the endogenous firing rates of the motor neurons, but they remain within the physiological capacity of type-Ib motor neurons to follow. To make this clearer, we have added a sentence to the Results text noting that each simulation corresponds directly to an experimental protocol, as indicated in the simulation panels (“Locomotion Assay,” “Ca^{2+} Pumping Assay,” “Electrophysiological Assay,” and “Metabolic Imaging”).

POINT 23 Figure 6

Figure 6 is complex and hard to understand and is not helped by its figure legend.

A) which are the individual APs? I presume they are the green line deviations as time progresses for NT, Ca2+ and Na+. This issue needs to be made clear. The "terminal at rest" is seen the Ca2+ - is there a "rest line for the NT and the Na+ graphs (not explained out).

We have made clarifying edits to the text of the legend.

B) Muscle 6 and 13 receive two MN inputs - I am assuming green = MN6/7-Ib and Blue is MN13-Ib, both fast firing MN inputs. However, the identity of the grey terminal endings in both muscle needs to mentions in the B's legend. Also what is type of MN input (grey) for muscle 13? Is not mentioned - i.e., is it a fast or slow MN?

We have made clarifying edits to the text of the legend.

B and C) graphs - it looks as if the Blue ATP consumption is superimposed over the top of the green ATP consumption, if so these are merged images - while this is fine, it would be ideal in Suppl Data to see the non-overlapping ATP consumption rates (e.g., a bit like only showing a merged fluorescent image without the separate channels shown)

We have added a line to register the upper extent of the otherwise obscured green trace (MN6/7-Ib).

E-I) as noted above, it would be interesting to model the ATP kinetics of the slow MN - MNSNd/d-Is for muscle 6.

As the slow firing MNs (MNSNb/d-Is) is not activated during the locomotion assay we feel that we do not have license to add this simulation. However, plots containing slow firing MNSNb/d-Is for muscle 6 are shown in figure 7A-G, as we did stimulate MNSNb/d-Is for the electrophysiology experiments and collected data from the combined input (includes MNSNb/d-Is).

J-K) Similar comments to E-I can also be applied to J-K.

Similarly, we did not collect data from MNSNb/d-Is terminals during our Ca^{2+} pumping assay and we feel that we do not have license to add this simulation in the absence of empirical data.

POINT24 Figure 7 A to N.

As noted in my comments about comparing fast and slow MN inputs and ATP kinetics, I see in Fig 7A this has been done for muscle 6 and reveals different responses for fast (green) and slow (red) MN inputs, but little comments is made above these differentness in the text (line 315-316) or on the Fig7's legend. Yes, I note that for most there is no difference - but A and C do show some difference between these two MN inputs, and so worthy of comment.

We thank the reviewer for noting the differences between fast- and slow-firing motor neurons in Fig. 7. As both axons are contained within the same hemisegment nerve it is technically very difficult to stimulate one axon at a supramaximal rate without also recruiting the other. Consequently, most of our electrophysiological experiments are designed to stimulate both axons at supramaximal rates and quantify the compound EJCs. For example, in Fig. 7A we used a frequency (60 Hz) that corresponds to ~7.7-fold above the endogenous firing rate of MNSNb/d-Is and ~2.8-fold above that of MN6/7-Ib. Because of this asymmetry, we chose not to extend the discussion into differences between the two MN types here, as this would become an “apples versus oranges” comparison. We have previously addressed these issues in detail under conditions where firing rates were controlled relative to each MN’s endogenous rate (Justs et al., 2023). In the present manuscript, which already integrates multiple experimental

approaches across eight figures, we feel that expanding on this point would detract from the main message.

We have now clarified our rationale in the text and legend of Fig. 7, give some discussion to Fig.7A-D as suggested, and also refer the reader to our prior analyses of differences between functionally differentiated terminal types -Ib and -Is.

POINT 25 Figure 7O to S

I note that the analyses were done on the one MN input to Muscle 13 (MN13-Ib), but the second MN input (presumable the grey input in Panel H) is also seen, but there are no analyses. Further there are two genotypes (normal ArgK1 (blue) and ArgK1 RNAi (knockdown) tested under two conditions (rest and stimulated). In panel "O" one needs to see the ArgK1 RNAi images under these two conditions to complement the changes shown in P-S for these two genotypes (i.e., the ArgK1 RNAi at rest and stimulated are not shown).

Consistent with each of our other imaging experiments, and the “behavioral” assay, we maintain our focus on type-Ib terminals.

We appreciate the point being made by the reviewer regarding the need to show the ArgK1 RNAi terminals at rest, and have now broken the SynaptopHluorin data out into a separate figure (#8), especially as these data represented empirical data tacked onto a simulation data figure.

We have also added representative images of SynaptopHluorin in terminals where ArgK1 has been knocked down.

POINT 26 Figure 7 O-S, needs to include the Biological 'N' numbers for this data set.

Biological “N” numbers for this data set are cited in the figure itself, but now also appear in the legend text.

POINT 27 Table 1 Line 336 could be also cited at key points in the results sections to help complement observations.

We have now cited the Table at each appropriate location throughout the narrative.

POINT 28 line 343 - Remove hyphens - "Nevertheless".

“Never-the-less” has now been edited to “Nevertheless”

POINT 29 Discussion A well-considered discussion. Perhaps as noted above, some comment about the roles of phosphagen kinases with respect to fast and slow firing MN terminals might

be of interest. In mammalian systems, MN firing patterns help to determine fiber type. How this determination is related to the drosophila neuromuscular system I am not sure. I raise this point as parts of this discussion works in comparing studies in the mouse versus the drosophila.

In the Discussion we have now clarified our interpretation of our electrophysiology data with respect to the separate inputs on muscle fiber #6, but in the absence of data we have not speculated further on differences between the two terminal types.

POINT 30 Role in endocytosis

Lines 428-433 While the evidence points to roles for ArgK1 in exocytosis - it would be good to see if there is an effect on endocytosis in these MNs (ArgK1 Vs ArgK1 RNAi), as suggested for hippocampal neurons. One could test for this idea using FM-143. Perhaps this can be noted as a future.

As suggested, we have noted that a deficit in endocytosis might be investigated through the application of styryl dyes such as FM1-43.

POINT 31 Method Knockdown of ArgK1 - lines 533-536. Details of the % of knockdown was pleasing to see. Nevertheless, I think the investigators would be well served to include this data, despite being published - shown in Suppl Data (See opening comments).

As suggested, previously published data showing quantification of ArgK1 knockdown has now been duplicated in the Supplementary Data (Fig. S3) and referred to from the text.

POINT 32 Methods Section 7 line 657

Perhaps a brief note on study design - namely was the study conducted blind to genotype (ArgK1 Vs ArgK1 RNAI) as per ARRIVE guidelines.

We agree with the reviewer that blinded experimental design represents the gold standard for minimizing bias. In this study, experiments were not conducted blind to genotype. It is worth noting that the only in vivo experiments are those shown in Fig. 5; the remainder of the data were obtained from ex vivo or in situ preparations. With respect to the ARRIVE guidelines, our understanding is that while these are recommended for vertebrate and cephalopod research funded by the NIH, they are not mandated for work in arthropods such as Drosophila. Consistent with this, IACUC approval is not required for studies in Drosophila.

POINT 33 Methods Section 8 ATP equations.

I think it would be helpful either in the main text or on the figure legends to make cross references to these equations used to generate the various graphs shown. I think will help reinforce the high quality and rigor of the data presented.

As suggested, the ATP consumption and production plots in figures 6 and 7 now link to the relevant formulas in the methods. The concentration plots in those same figures, however, are not generated by any one equation. Rather, we numerically integrate ATP production and consumption, each of which is represented by a number of equations. New captions in figures 6 and 7 now clarify this point. Additionally, the methods now contain a link to the full simulation code.

Dear Professor Macleod,

Re: JP-RP-2025-288916R1 "**Optimal Neuromuscular Performance Requires Motor Neuron Phosphagen Kinases**" by Karlis A Justs, Danielle V Latner, Carlos D Oliva, Yosuf Arab, Gabriel G Bonassi, Olena Mahneva, Sarah Crill, Sergio Sempertegui, Paul A Kirchman, Yaouen Fily, and Gregory T Macleod

We are pleased to tell you that your paper has been accepted for publication in The Journal of Physiology.

The last Word (or similar) version of the manuscript provided will be used by the Production Editor to prepare your proof. When this is ready you will receive an email containing a link to Wiley's Online Proofing System. The proof should be thoroughly checked and corrected as promptly as possible.

Authors should note that it is too late at this point to offer corrections prior to proofreading. Major corrections at proof stage, such as changes to figures, will be referred to the Editors for approval before they can be incorporated. Only minor changes, such as to style and consistency, should be made at proof stage. Changes that need to be made after proof stage will usually require a formal correction notice.

All queries at proof stage should be sent to: TJP@wiley.com.

Following copyediting and typesetting the accepted version will be published online.

Yours sincerely,

Katalin Toth
Senior Editor
The Journal of Physiology

IMPORTANT POINTS TO NOTE FOLLOWING ACCEPTANCE OF YOUR PAPER:

• IMPORTANT NOTICE ABOUT OPEN ACCESS: To assist authors whose funding agencies mandate public access to published research findings sooner than 12 months after publication, The Journal of Physiology allows authors to pay an Open Access (OA) fee to have their papers made freely available immediately on publication.

The Corresponding Author will receive an email from Wiley with details on how to register or log-in to Wiley Authors Services where you will be able to place an order.

• You can check if your funder or institution has a Wiley Open Access Account here: https://authorservices.wiley.com/author-resources/Journal-Authors/licensing-and-open-access/open-access/author-compliance-tool.html.

• TRANSPARENT PEER REVIEW POLICY: To improve the transparency of its peer review process, The Journal of Physiology publishes online as supporting information the peer review history of all articles accepted for publication. Readers will have access to decision letters, including Editors' comments and referee reports, for each version of the manuscript as well as any author responses to peer review comments. Referees can decide whether or not they wish to be named on the peer review history document.

• You can help your research get the attention it deserves! Check out Wiley's free Promotion Guide for best-practice recommendations for promoting your work at: www.wileyauthors.com/eeo/guide. You can learn more about Wiley Editing Services which offers professional video, design, and writing services to create shareable video abstracts, infographics, conference posters, lay summaries, and research news stories for your research at: www.wileyauthors.com/eeo/promotion.

• If you would like to receive our 'Research Roundup', a monthly newsletter highlighting the cutting-edge research published in The Physiological Society's family of journals (The Journal of Physiology, Experimental Physiology, Physiological Reports, The Journal of Nutritional Physiology and The Journal of Precision Medicine: Health and Disease), please click this link, fill in your name and email address and select 'Research Roundup':
https://www.physoc.org/journals-and-media/membernews

EDITOR COMMENTS

Senior Editor:

No further comments

REFEREE COMMENTS

Referee #1:

The authors have adequately addressed the concerns I raised in the previous round of review with textual modifications. I have no further concerns regarding the manuscript.

Referee #2:

I am most pleased that the authors have taken the time and effort to address all my comments in a considered and thoughtful manner. I have no further concerns.
